# Identifying the known and unknown health hazard information for chemical disasters: a phased scoping review of the East Palestine, Ohio train derailment

**DOI:** 10.1038/s41370-025-00803-0

**Published:** 2025-09-12

**Authors:** Ruth M. Lunn, Meredith Clemons, Robyn Blain, Somdat Mahabir, Suril S. Mehta, Andrew A. Rooney, Anisha Singh, Stephanie Smith-Roe, Kyla W. Taylor, Wren Tracy, Maricruz Zarco, Suzanne E. Fenton

**Affiliations:** 1 Division of Translational Toxicology, National Institute of Environmental Health Sciences, Research Triangle Park, Triangle Park, NC USA; 2 ICF, Reston, VA USA; 3 Epidemiology and Genomics Research Program, Division of Cancer Control and Population Sciences National Cancer Institute, Rockville, MA USA; 4 Present Address: Institute of Epidemiology and Medical Biometry, Ulm University, Ulm, Germany; 5 Present Address: Center for Human Health and the Environment, NC State University, Raleigh, NC USA

**Keywords:** Environmental contamination, chemical emergencies, East Palestine derailment, human health hazards, scoping literature review, rapid review methods

## Abstract

**Introduction:**

In February 2023, people residing in the village of East Palestine (EP, Ohio, USA) and surrounding areas were exposed to toxic chemicals from a Norfolk Southern Railway train derailment and subsequent vent and burn.

**Objective:**

To identify known health hazards and evidence gaps from these chemicals to inform disaster-response research.

**Methods:**

We conducted a rapid phased literature scoping review. In Phase 1, we summarized major conclusions from eight authoritative sources across ~15 health hazard categories for 22 chemicals potentially related to the train derailment and response. In Phase 2, we conducted targeted literature searches in PubMed for higher-priority chemicals and outcomes with research gaps, considering the recency of authoritative reviews. Finally, we summarized findings from the retrieved studies and those from authoritative reviews to further characterize evidence gaps and the next steps.

**Results:**

Eight higher-priority chemicals were skin and eye irritants, seven of which were also respiratory irritants, consistent with symptoms reported by East Palestine residents and workers. Five chemicals were human or animal carcinogens; two may cause adverse immunological or neurological effects, and one may cause damage to reproductive organs or the developing fetus. Vinyl chloride had the most comprehensive data. After Phase 2 literature searches, we suggested the need for primary studies for 12 chemical outcome pairs and a systematic review for two pairs.

**Significance:**

Our rapid literature scoping approach can provide knowledge for researchers conducting community studies and public health officials who communicate with the affected community on the known and unknown health hazards of chemicals related to the East Palestine train derailment. It also informs global disaster-response-related research, as these chemicals are commercially important and have been detected in other chemical release incidents. Moreover, our rapid literature scoping phased approach can be leveraged for environmental emergencies when the need for health hazard information is urgent.

**Impact:**

Our rapid literature scoping approach can provide knowledge for researchers conducting community studies and public health officials who communicate with the affected community on the known and unknown health hazards of chemicals related to the East Palestine train derailment. It also informs global disaster-response-related research, as these chemicals are commercially important and have been detected in other chemical release incidents. Moreover, the phased approach used for our rapid literature scoping review can be leveraged for environmental emergencies when the need for health hazard information is urgent.

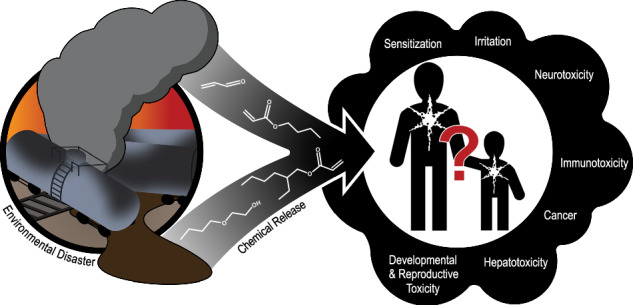

## Introduction

At least every two days in the United States, a serious chemical hazard incident occurs—including fires, explosions, and toxic releases—according to a database of 875 incidents reported in the media since January 1, 2021, compiled by the Coalition to Prevent Chemical Disasters [1]. Of the 323 incidents reported in the Media in 2023, 14 involved railway accidents releasing hazardous chemicals. One such incident occurred on February 3, 2023, when a Norfolk Southern Railway general merchandise freight train derailed, releasing vinyl chloride and other dangerous substances into the environment (e.g., air, water, soil) in East Palestine, Ohio, and the surrounding area. Three days later, first responders conducted a vent and burn of five cars containing vinyl chloride to prevent an explosion, releasing volatile organic compounds (e.g., acrolein, benzene) into the air and potentially releasing other residual chemicals in the soil and water. To aid site clean-up, Unified Command—which includes the U.S. Environmental Protection Agency (EPA) and local/state governments—has monitored, and continues to monitor, the air, soil, and water for known chemicals of interest. Available monitoring data are published for public access on EPA’s East Palestine, Ohio Train Derailment web page [2]. A few academic researchers, such as those participating in the National Institute of Environmental Health Sciences Disaster Research Response (DR2) Program Network of environmental health researchers, have also conducted environmental monitoring, such as mobile monitoring, and published [3] and shared this data with the DR2 network and others.

Following the derailment, additional efforts aimed to collect information on symptoms experienced by the community. The Agency for Toxic Substances and Disease Registry (ATSDR) collaborated with the Pennsylvania and Ohio Departments of Health to conduct an Assessment of Chemical Exposures (ACE) Survey on community health [4] and a First Responder Survey [5], both of which queried symptoms reported through March 31, 2023. First responders arrived on site the night of the derailment, and many contributed to the management of the vent and burn [6], and residents within a mile radius had been evacuated from the site between the derailment and vent and burn [6]. Community members, Centers for Disease Control and Prevention (CDC) survey workers [7], and first responders reported short-term adverse symptoms (e.g., headaches, respiratory effects) consistent with acute chemical irritant exposure, as well as adverse mental health symptoms (residents). Headaches, anxiety, and coughing were more common among residents [8], and nasal and lung effects [9] were more common among first responders.

Disasters releasing hazardous materials occur unexpectedly yet regularly, and decision-makers depend on timely access to high-quality, actionable information to protect public health. Rapid reviews and timely access to high-quality information [10]—to identify what we know and what we do not know about potential health effects—can aid in decision making, communication, and research planning.

### Objective

We conducted a rapid, phased literature scoping review to identify and summarize known health hazards and research needs. Information gleaned early can be used to inform communications with affected communities as well as early steps taken to prevent or minimize adverse health consequences while more comprehensive literature reviews are underway. The information can also identify the need for, or to support the development of, short or long-term research studies or health monitoring strategies.

**Fig. 1:**
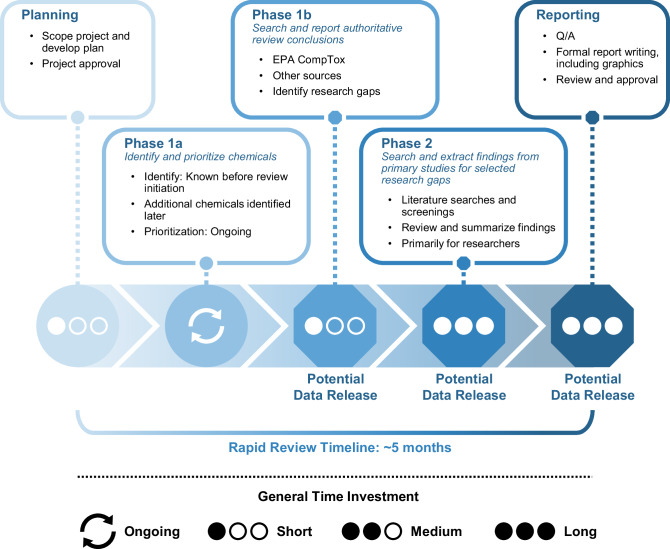
East Palestine rapid review project timeline. Overview of the timeline for the rapid review and phased approach, with information on potential points for early data release.

The review was developed to inform a forthcoming National Academy of Science, Engineering, and Medicine workshop [11] sponsored by the National Institute of Health and Centers for Disease Control and Prevention – Agency for Toxic Substances and Disease Registry. The date of the workshop was unknown when the review was initiated. Our phased approach allowed us to create products of varying complexity for potential delivery at different time points (e.g., depending on the timing of the workshop) (see Fig. 1). Collated findings from authoritative sources were expected to be the first potential product. Over time, additional chemicals and supplemental information expected to aid researchers were incorporated into the review. Ultimately, the workshop, “Public Health Research and Surveillance Priorities from the East Palestine, Ohio Train Derailment: A Workshop,” was held on November 6 and 7, 2023. This rapid literature scoping review is an adaptation of the publicly available NIEHS report and contributes to broader NIEHS research [10] efforts in response to the derailment.

## Materials and Methods

We developed a phased approach to conduct a rapid review of the literature on human health hazards of the chemicals released during and after the derailment. While this work was done for the aforementioned workshop and the timeline was not intended to guide immediate response to the East Palestine disaster, the process can be adapted for more timely real-time use in future disasters.

The literature scoping review process began in late May 2023 and included the following phases, which were iterative: (1) Literature scoping and evidence map: Identification of chemicals of interest (Phase 1a); (2) Literature scoping, evidence map, and summary: Identification and summary of authoritative review conclusions on selected health outcomes for chemicals of interest (Phase 1b); and (3) Targeted literature searching, screening, and summary: Summary of literature reviews and primary studies regarding key research gaps contributing to uncertainty on potential health effects for higher-priority (i.e., highest- and high-priority) chemicals (Phase 2). Fig. S1 provides an overview of the steps undertaken for Phase 1a and Phase 1b.

### Phase 1a: Identification and prioritization of chemicals of interest

Because of the emergent nature of the chemical releases, it was difficult to obtain definitive information on the range of chemicals of concern following the train derailment. For this first phase, chemicals of interest were those that were released or suspected to be released during the train derailment and vent and burn response, with the potential for human exposure. We identified 16 primary chemicals of interest to review for available health effects data. The list of chemicals (see Table S1) came from publicly available media sources [12] reporting on the train car contents that Norfolk Southern Railway reported to EPA [13] (consistent with the Texas A&M University [TAMU] DR2 unit list) and internal communications. Several chemicals were reported to have been carried on the derailed train (e.g., benzene residue, butyl acrylate, ethylene glycol monobutyl ether, ethylhexyl acrylate, several glycol chemicals, isobutylene, polyethylene, polyvinyl, petroleum lube oil, vinyl chloride) [14–16]. Other chemicals were suspected to be of concern because of their potential release during the vent and burn (e.g., acrolein, hydrogen chloride, phosgene gas) [15, 16].

Next, we conducted an initial prioritization (i.e., higher vs. lower) of the 16 primary chemicals to identify those with a higher likelihood of potential human exposure following the train derailment and/or subsequent fire. Monitoring data from the EPA Sample Monitoring Dashboards [2] or academic researchers [3] were available on select chemicals (acrolein, benzene, butyl acrylate, diethylene glycol, ethylene glycol monobutyl ether (“2-butoxyethanol”), 2-ethylhexyl acrylate, 1,2 propylene glycol, vinyl chloride). Although some chemicals present in the derailed cars or suspected to have been released in the subsequent vent and burn were not reported in the dashboard (e.g., dipropylene glycol, hydrogen chloride, isobutylene, petroleum lube oil, phosgene gas, polyethylene, polypropylene glycol, polyvinyl alcohol), these chemicals were included to gather information on the range of potential exposures experienced by residents; additionally, some were discussed in media reports after the accident [17]. Based on informal correspondence with experts involved in the derailment response, the eight higher-priority chemicals were acrolein, benzene, butyl acrylate, 2-butoxyethanol, 2-ethylhexyl acrylate, hydrogen chloride, phosgene gas, and vinyl chloride. However, all chemicals were reviewed for available health-related evidence.

During the initial literature scoping and review efforts, we identified additional chemicals—dioxins and per- and polyfluoroalkyl substances (PFAS)—that were potentially released during the vent and burn or its response. These are collectively referred to as “potentially related chemicals” because of greater uncertainty about the potential for exposure. Dioxins may have been formed as a chemical byproduct of the vent and burn, and firefighting foams used to retard the burn were suspected to contain PFAS. We included dioxins in our review because community members raised concerns [18] about the potential for exposures following media reports of elevated levels of dioxins in the soil near the derailment site (compared with towns in Ohio with industries somewhat similar to those in East Palestine) [18, 19]. EPA, in collaboration with Norfolk Southern, also conducted soil sampling for dioxins one to two months after the derailment and, in general, did not find that dioxin levels were higher than background levels [20]. Additionally, blood sampling of 18 East Palestine residents found that serum dioxin levels were consistent with those expected in the broader U.S. population based on comparisons with data from NHANES 2011-2012 by sex, age, and ethnicity [21].

The burn was extinguished using water containing approximately 40 gallons of T-STORM F-787A alcohol-resistant aqueous film-forming foam (AR-AFFF) known to contain PFAS [22]. Common, smaller chain breakdown products of these PFAS have been reported [23], and some terminal PFAS are estimated to persist in the environment for up to a century without remediation [23]. While much of the product’s formulation is confidential, this mixture is believed to contain PFAS typical of other ANSUL® products [24–26], among other substances such as 2-butoxyethanol and polyethylene glycol—two chemicals included in this review. The five PFAS suspected in the AR-AFFF based on the formulation of other ANSUL® products were 6:2 fluorotelomer sulfonamido amine oxide (6:2 FTNO), 6:2 fluorotelomer thiohydroxyammonium (6:2 FTSHA), 6:2 fluorotelomermercap-toalkylamido sulfonate (6:2 FTSAS), 6:2 fluorotelomer sulfonic acid (6:2 FTSA), and 6:2 fluorotelomer sulfonamide alkylbetaine (6:2 FTSA-PrB). Firefighters who applied the AR-AFFF would be expected to have a high potential of exposure to these PFAS [27, 28].

Because many of the chemicals of interest are commercially important and regularly used or transported throughout the United States [29] (see Table S1), we also searched EPA’s Chemical Data Reporting (CDR) database [29] under the Toxic Substances Control Act (TSCA) to identify recent nationally aggregated production volumes for the 22 chemicals of interest. All chemicals except 6:2 FTSAS, 6:2 FTNO, and dioxins were identified in EPA’s 2020 CDR files for industrial processing and use, consumer and commercial use, and manufacture-import.

### Phase 1b: Summary of authoritative review conclusions

**Fig. 2:**
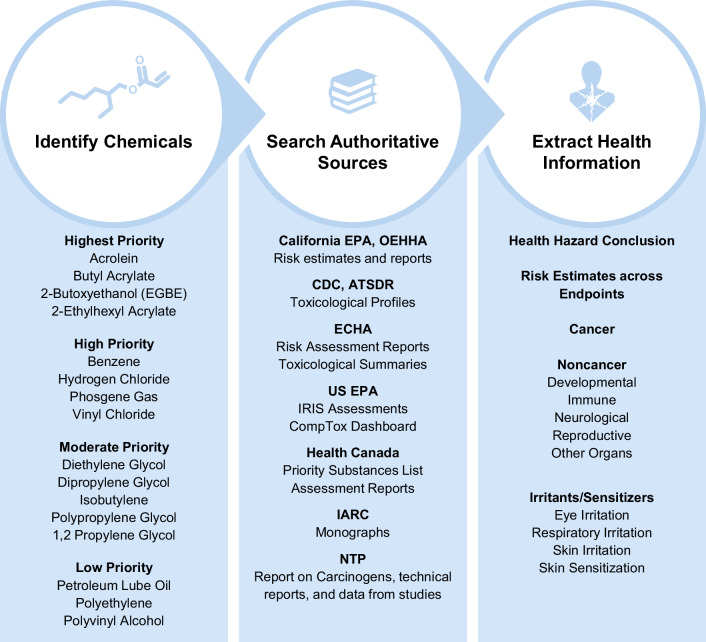
Phase 1a Chemical identification and Phase 1b authoritative sources and health outcome categories. Summary of Phases 1a and 1b to identify chemicals, search authoritative sources, and identify health hazard information. A list of the chemicals and their final priority structure is provided, along with a list of authoritative sources searched and categories of health hazards extracted in Phase 1b.

To inform our understanding of potential health effects and key data gaps for the 22 chemicals, including the 16 primary chemicals of interest and six potentially related chemicals, we searched for and extracted data on human health hazards available from selected authoritative sources (see Fig. 2). All these authoritative sources were searched using the CASRN (Chemical Abstracts Service Registry Number) available for each chemical. If no results were returned by searching for the CASRN, the chemical name was used. Searches were initially completed in the EPA’s CompTox Dashboard [30], which provides an extensive list of available risk assessment, hazard, and toxicity values from a variety of sources, followed by searches in the seven authoritative sources listed in Fig. 2.

Data from authoritative sources were extracted for the 15 health outcome categories listed in Fig. 2, starting with EPA’s CompTox Dashboard, which was supplemented by the other sources. Human health hazard conclusions were prioritized for extraction, along with human-relevant risk estimates (e.g., cancer slope factors [CSFs], minimal risk levels [MRLs], reference doses [RfDs], reference concentrations [RfCs], recommended exposure limits [RELs], do-not-exceed limits [DNELs]). If human health hazard conclusions or risk estimate values were not available, we made note of available toxicity values (e.g., points of departure [POD], lethal dose or concentration at which 50% of the study population does not survive [LD50 or LC50]) and critical effects. If none of the above information was available, we noted whether human and/or animal data were available for the health outcome category. Although our extractions did not specifically capture data from mechanistic studies, authoritative sources often integrate mechanistic information with other data types to develop hazard conclusions. If data gaps were identified for a chemical, additional sources were searched for information, including National Academies’ Acute Exposure Guideline Level (AEGL) Reports [31, 32] for select chemicals (phosgene gas and hydrogen chloride) and reports from the World Health Organization (WHO) and Organisation for Economic Co-operation and Development (OECD).

We completed data extraction in Microsoft Excel from May to August 2023. During report development, we learned of an updated draft Toxicological Profile for Benzene published by ATSDR for public comment in 2024 [33]. We reviewed the report and updated our findings accordingly; however, we did not conduct comprehensive searches to identify updated authoritative sources across the chemicals. In addition to publication information (e.g., source name, access link, date of publication), we extracted the following information for each health outcome category (when applicable): hazard conclusion or risk estimate and critical effect, evidence type (i.e., human or animal; extractions did not aim to capture mechanistic data), duration and route of exposure, and additional relevant information (as necessary). A primary extractor reviewed and extracted the health effects data from all sources for each chemical, and the extraction was checked for completeness and accuracy by a secondary extractor.

Following initial extraction, we compiled results into a chemical-outcome summary file (see Supplemental Excel Table S1) to better understand data gaps across the chemicals and health outcome categories. For each chemical, the health hazard conclusions, relevant risk estimates, and data were extracted, organized, and summarized (Fig. 2 and S1). A list of authoritative sources (with links to web pages and access dates) with available data for each chemical was also included (see Supplemental Excel Table S2 and S4).

As a first step to quickly determine if additional research had been conducted since the authoritative reviews, we conducted additional targeted searches in the Causaly platform [34] for all primary chemicals using the term “diseases affected” by chemical name. Causaly uses artificial intelligence to rapidly search the body of available biomedical literature for a given chemical. The output provides a list of health outcome categories identified in the published literature for the chemical, the projected relationship between the chemical and the health outcome (e.g., upregulated, downregulated, bidirectional), and the citation for the published literature. If the Causaly results indicated that available literature addressed an identified gap, we reviewed the citations to better understand the available evidence. Applying this information, we prioritized additional health outcome categories with suggestive data from Causaly.

Using expert judgment, we harmonized data and conclusions for all health outcomes for each chemical from different authoritative sources using the guidance provided in Figure S2 (see extraction results in the chemical-outcome summary file in the Supplemental Excel Table S1) into categories (conclusions: higher, moderate, or lower confidence/severity, limited animal evidence, inadequate evidence, not likely a risk). If no conclusions were available in authoritative reviews (i.e., a research gap was identified), categories were “no or few studies” or “evidence suggestive” based on the available data. Figure S2 provides additional information on each category.

### Identification of key research gaps

Research gaps (identified above) for specific chemical and health outcome (“chemical × health outcome”) pairs were considered for additional targeted searches in PubMed.

Chemical × health outcome research gaps were further prioritized (highest, high, moderate, and low) and selected according to the following criteria: 1) higher priority of the chemical based on potential for human exposure following release (see Phase 1a: Identification of Chemicals of Interest), (2) no definitive hazard conclusions available, (3) lack of a recent hazard conclusion, and (4) a lack of data or conclusions for humans.

### Phase 2: Targeted literature searches and screenings

#### Searching the literature

In Phase 2, we conducted literature searches to identify primary and review articles relevant to the chemical × health outcome pairings identified in Phase 1 reviews. We structured searches to capture the available human and animal studies on these pairings; however, some review articles may have also included mechanistic data. In two instances, we did not conduct the search for primary articles because of the large volume of reviews returned from the initial search (see results for acrolein × nervous and dioxins other than TCDD × any health outcome).

Given the goal of conducting a rapid review, we conducted a more streamlined but relatively comprehensive search using one bibliographic database. We used search terms to identify the appropriate chemical, health outcome, evidence type (e.g., human or animal), and study type to search for relevant primary and review articles. We developed separate chemical search strings for each chemical of interest. We identified chemical search terms using EPA’s CompTox Dashboard list of “valid” and “good” chemical synonyms. We also searched the chemical in PubMed’s Medical Subject Headings (MeSH) thesaurus to identify and retrieve indexing terms. We identified search terms for each health outcome, evidence type, and epidemiological study design using SWIFT-Review’s publicly available Search Strategies Word documents [35] and the National Toxicology Program’s (NTP’s) Report on Carcinogens literature search approach [36]. We then translated terms into the appropriate syntax for use in PubMed (see Supplemental Materials, Search Strings).

We compiled search strings for the chemical and health outcome pairings identified after Phase 1 (see Table S2). Additionally, we specified which searches should return human versus animal evidence.

We conducted searches in PubMed June–August 2023. For chemical × health outcome pairs involving cancer, if the Phase 1 results included a recent review from the International Agency for Research on Cancer (IARC) (i.e., those published in the five years prior to June–August 2023), we added a date restriction to the search to identify studies published after the IARC publication date and up to June or July 2023, when searches were conducted. The Population, Exposure, Comparator, and Outcome (PECO) criteria used to guide the Phase 2 screening and additional details on relevant outcome categories are available in Tables S2 and S3.

Following Phase 1 extractions, for the five PFAS of interest, we explored EPA systematic evidence mapping (SEMs) [37–39] reviews for a variety of PFAS to inform our PubMed literature search strategy. Research on emerging PFAS has been rapidly increasing in recent years. These SEMs returned one study for 6:2 FTSHA, no literature for 6:2 FTSAS or 6:2 FTSA-PrB and did not include 6:2 FTNO. As a result, we did not pursue these chemicals further. However, studies were available for 6:2 FTSA [37]. To identify additional information about these chemicals, we conducted a nonrestricted literature search for 6:2 FTNO and a literature search to identify publications post-dating EPA’s most recent search for 6:2 FTSA. Searches were conducted in PubMed in August 2023. We developed chemical search strings using the approach outlined above; however, to identify as much available literature as possible, searches were not otherwise restricted by health outcome category, evidence type, or study type. Literature search details for relevant PFAS are included in Table S2.

For dioxins, most of the authoritative sources extracted in Phase 1 reported hazard information for TCDD, a dioxin often used as a proxy for the broader class. Phase 2 searches and screenings aimed to identify reviews of other dioxins (i.e., not TCDD only). We conducted searches in PubMed to identify reviews published in the five years prior to August 2023 and were not restricted by health outcome category or evidence type. Literature search details and search strings are in the Supplemental Materials, and Table S2.

#### Selecting the studies

For title-abstract screening, we uploaded results from literature searches to DistillerSR [40], a platform for literature screening and management. For each reference, one screener reviewed the title and abstract and indicated PECO relevance. For the purposes of this review, we considered case reports/series and worker surveillance studies as PECO relevant. References without an abstract were screened based on the title only. A senior-level screener reviewed ten percent of all excluded references as a quality control and assurance measure. We then reviewed relevant references identified during title-abstract screening at the full-text level in DistillerSR.

#### Summarizing the studies

Relevant references underwent tagging and data extraction in DistillerSR. For relevant review articles and primary studies, we extracted information on the publication type, study design, evidence type, population characteristics (human), exposure conditions (animal), health outcomes, and findings. A primary screener conducted data extraction, and a senior-level screener reviewed the extraction for quality control and assurance purposes.

For each chemical × health outcome pairing with PECO-relevant studies, we summarized studies identified in the literature searches along with the results from studies reported in the authoritative reviews (Phase 1b).

## Results

### Phase 1

**Fig. 3:**
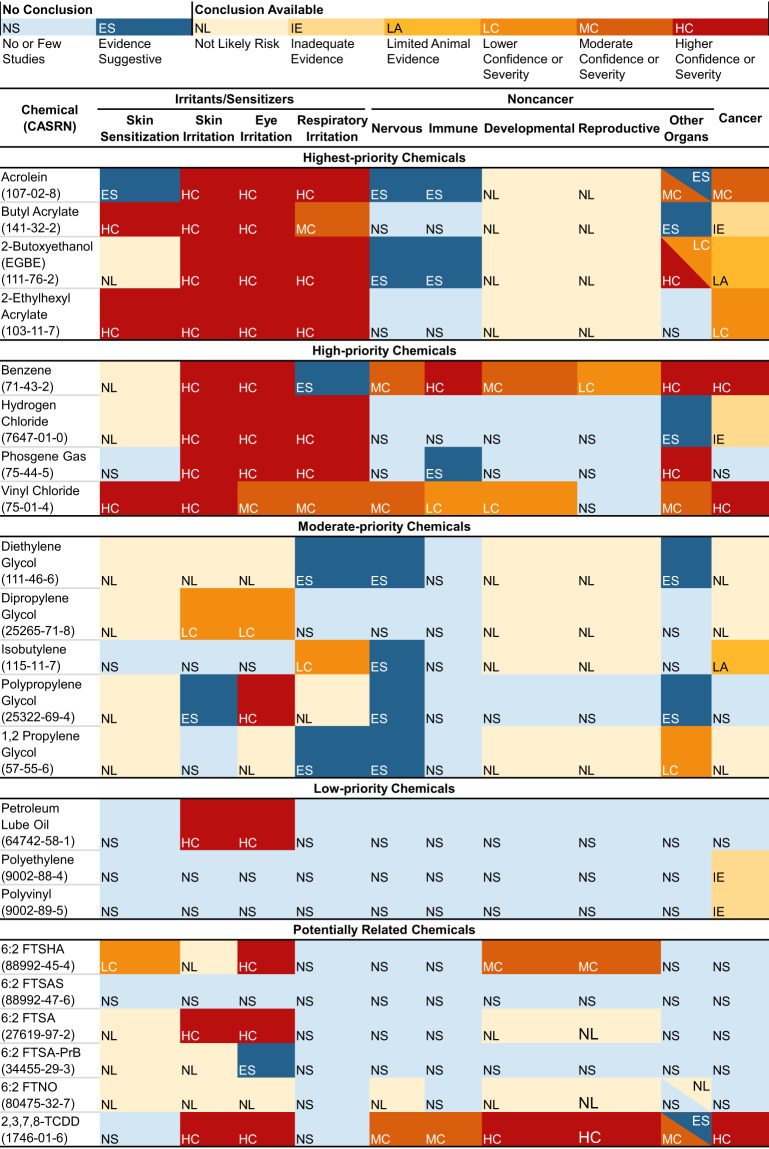
Summary of findings from authoritative sources^a^ for 16 Primary East Palestine chemicals of interest and potentially related East Palestine Chemicals. Summary of conclusions and other information from authoritative sources for 16 primary chemicals of interest and five PFAS chemicals and TCDD. See Fig. 2 and Tables S5 and S6 for more details. A list of authoritative sources is available in Fig. 2. For vinyl chloride and skin sensitization, one source reported Category 1 sensitization and another source reported that classification was not possible. “Conclusions available” relate to language used by authoritative sources as follows (see Fig. S2) : inadequate evidence (IE) = nonclassifiable (cancer); limited animal evidence (LA) = nonclassifiable (cancer); lower confidence or severity (LC) = possibly (cancer), suspected (noncancer), irritant category with other conflicting data, conflicting sensitizing data; moderate confidence or severity (MC) = probably (cancer), presumed (noncancer), animal data-derived risk estimate value, Category 3 (irritant), Category 1B (sensitizer); higher confidence or severity (HC) = known (cancer, noncancer), human data-derived risk estimate value, Category 1 and 2 (irritant), Category 1 A (sensitizer). 6:2 FTNO = 6:2 fluorotelomer sulfonamido amine oxide; 6:2 FTSHA = 6:2 fluorotelomer thiohydroxyammonium; 6:2 FTSAS = 6:2 fluorotelomermercap-toalkylamido sulfonate; 6:2 FTSA = 6:2 fluorotelomer sulfonic acid; 6:2 FTSA-PrB = 6:2 fluorotelomer sulfonamide alkylbetaine; PFAS = per- and polyfluoroalkyl substances; TCDD = 2,3,7,8-tetrachlorodibenzo-p-dioxin.

We reviewed and extracted a total of 134 resources (e.g., reviews, reports, databases) from authoritative sources across the 22 chemicals in Phase 1 (see Supplemental Excel File Table S1). Phase 1 results summarize and collate existing conclusions from authoritative sources on health outcomes associated with each chemical. We created an overview/map of the evidence (see Fig. 3) using the collated health outcome data from each authoritative source for the 22 chemicals (see Tables S4, S5, and S6 for supporting information; Table S5 [priority chemicals] and Table S6 [potentially related chemicals] summarize the health hazard conclusions and information across the authoritative sources). The maps provide information on whether, for each chemical, there was a health hazard conclusion and, if so, the confidence of the evidence or severity of the outcome (e.g., higher, moderate, lower) stratified by chemical priority (see Methods). If authoritative conclusions were not available, the maps indicate whether the studies in the reviewed sources suggested an association or if no/few studies were available. For some health outcomes (e.g., endocrine, immune, gastrointestinal, renal) there were few authoritative sources identified for chemicals. These health categories were combined as “Other Organs” for reporting purposes. We integrated determinations based on additional data from the targeted Causaly reviews with information from the authoritative reviews for select chemicals (acrolein; 2-butoxyethanol; diethylene glycol; dipropylene glycol; hydrogen chloride; and 1,2 propylene glycol).

#### Irritation and sensitization

##### Primary chemicals

**Fig. 4:**
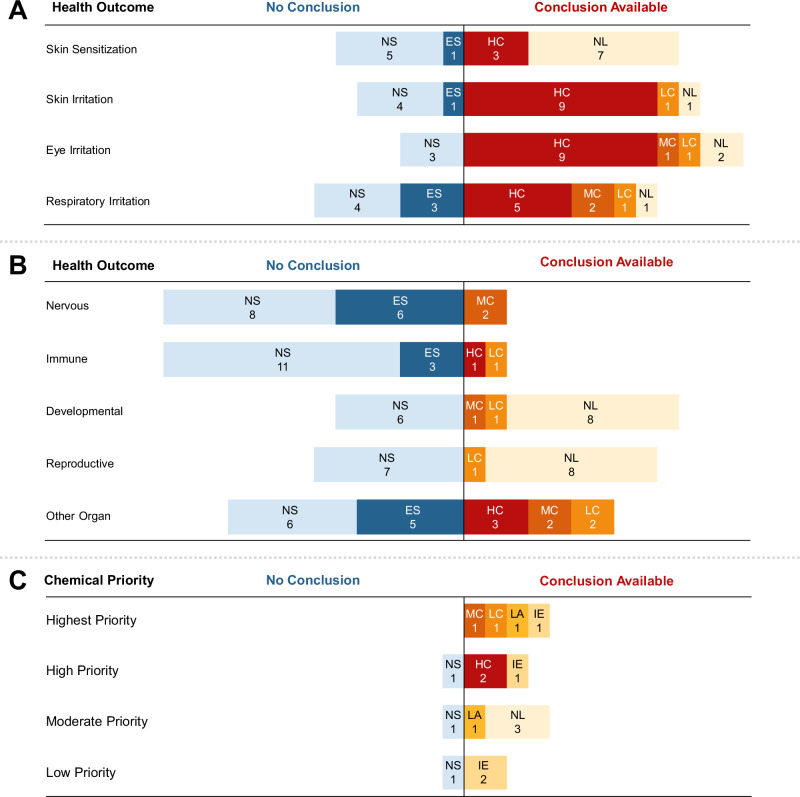
Authoritative conclusions for 16 primary chemicals: A. irritation and sensitization, B. Noncancer outcomes, and C. Cancer outcomes. Summary of irritant and sensitizer information, non-cancer and cancer outcomes from authoritative sources for 16 primary chemicals of interest. Detailed findings for each chemical × health outcome category are reported in Table S5 and Supplemental Excel Table S1. The “Other Organ” category includes double counts for two chemicals (acrolein and 2-butoxyethanol), wherein different conclusions were available for different organ systems NS = no or few studies, ES evidence suggestive, NL not likely to be irritating or sensitizing or risk for health outocme, LC lower confidence, MC moderate confidence or severity, HC higher confidence or severity. See Fig. S2 for more details.

Testing or health characterization of chemicals was the most complete for acute effects such as irritation or sensitization (see Fig. 4). Most chemicals were classified as causing skin (10 of 16 chemicals) or eye (11 of 16 chemicals) irritation, including all the higher-priority (i.e., highest and high-priority) chemicals, two moderate-priority chemicals (dipropylene glycol for both outcomes and polypropylene glycol for eye), and one low-priority chemical (petroleum lube oil for both outcomes). Among the higher-priority chemicals, risk assessment values were not found for skin irritation, and available values for eye irritation ranged from 0.0025 mg/m^3^ for acrolein to 180 mg/m^3^ for vinyl chloride (see Table S5). The reviews concluded that diethylene glycol was not associated with skin or eye irritation, and 1,2 propylene glycol was not associated with eye irritation. No other conclusions were identified for the other moderate- and low-priority chemicals. Half of the chemicals caused respiratory irritation, seven of which were higher-priority chemicals. Polypropylene glycol was not considered a respiratory irritant. Risk assessment values for high-priority chemicals and respiratory irritation ranged from 2 × 10^−5^ mg/m^3^ for acrolein to 180 mg/m^3^ for vinyl chloride (see Table S5). No definitive authoritative conclusions were found for benzene, three of the five moderate-priority chemicals, and all three low-priority chemicals. However, the reviews reported individual study findings for benzene, diethylene glycol, and 1,2-propylene glycol. Adverse respiratory effects or irritation from multiple chemicals are consistent with reported symptoms (e.g., running nose; congestion; coughing; burning nose, throat, or eyes; irritation) from the affected community and first responders in CDC’s ACE Survey [4].

While some authoritative sources discussed skin sensitization in the context of immune effects, skin sensitization was characterized as a separate outcome category in this review. Three higher-priority chemicals were categorized for skin sensitizing effects (butyl acrylate, 2-ethylhexyl acrylate, and vinyl chloride). The European Chemicals Bureau (ECB) [41] discussed sensitizing data for acrolein, another higher-priority chemical, but did not categorize it for sensitization. Little or no indication of skin sensitization was reported for seven chemicals (2-butoxyethanol, benzene, hydrogen chloride, diethylene glycol, dipropylene glycol, polypropylene glycol, and 1,2-propylene glycol). No conclusions were available for the remaining chemicals.

##### Potentially related chemicals

For PFAS, one chemical (6:2 FTSA) was classified for corrosive effects to the skin, whereas three chemicals (6:2 FTSHA, 6:2 FTSA-PrB, and 6:2 FTNO) did not have irritating effects to the skin in studies reported in Phase 1 sources. Conclusions for eye irritation were available for two chemicals (6:2 FTSHA and 6:2 FTSA); some irritation was reported following exposure to 6:2 FTSA-PrB in rabbits, and no eye irritation was predicted for 6:2 FTNO. Data were not available for categorizing irritating effects to the respiratory system following PFAS exposures. Authoritative sources reported little or no concern for skin sensitization following exposure to three chemicals (6:2 FTSA, 6:2 FTSA-PrB, and 6:2 FTNO). Mixed results were reported from animal and in vitro studies for one chemical (6:2 FTSHA). Skin sensitization data were not available for the remaining PFAS (6:2 FTSAS). TCDD was classified as causing irritation to both the skin and eyes. Additionally, reviews emphasized that high-level exposures to TCDD can cause moderate to severe chloracne, an acne-like eruption of the skin, and other skin rashes and discoloration. There were no conclusions for TCDD and respiratory irritation, although one review reported upper respiratory irritation in humans following inhalation. Skin sensitization was not classified for TCDD because few data were available. No chemicals were forwarded to Phase 2 for these outcomes because there were no gaps for the highest priority chemicals.

#### Other noncancer outcomes

##### Primary chemicals

Authoritative conclusions for reproductive and developmental toxicities were available for approximately half of all chemicals (see Fig. 4 for noncancer outcome findings). Of the eight higher-priority chemicals (see Fig. 3 for chemical-specific determinations and priority categories), two chemicals (benzene and vinyl chloride) may be linked to developmental effects based on determinations. California Environmental Protection Agency (CalEPA) Proposition 65 [42] lists benzene as a chemical that can cause developmental and reproductive effects; other reviews agreed with the developmental effects conclusions (particularly for developmental hematotoxicity) but stated that the evidence for reproductive effects was limited. Available risk assessment values for developmental effects from inhalation exposure (mg/m^**3**^) are 0.027 for benzene (CalEPA Office of Environmental Health Hazard Assessment [OEHHA] REL for acute exposure) and 1.30 for vinyl chloride (ATSDR MRL) (see Table S5). Although positive associations have been observed for adverse reproductive (e.g., effects to reproductive organs or in pregnant animals) or developmental outcomes and exposure to several higher-priority—acrolein, 2-butoxyethanol, butyl acrylate (developmental only)—and moderate-priority chemicals—diethylene glycol—these effects occurred at high doses or at doses causing maternal toxicity. As a result, several authoritative sources (primairly ECHA) concluded that the chemicals are not significantly toxic to reproduction or the developing fetus. There was low concern for reproductive toxicity following exposure to butyl acrylate based on studies showing no adverse effects. There was also low concern for developmental and reproductive toxicity for 2-ethylhexyl acrylate and two moderate-priority chemicals, isobutylene and 1,2 propylene glycol. Unclear or no conclusions were available for both reproductive and developmental effects for the remaining higher-priority chemicals: hydrogen chloride and phosgene gas.

Health outcome conclusions were typically not available for neurotoxicity (14 of 16 chemicals) and immunotoxicity (14 of 16 chemicals). Vinyl chloride was deemed a presumed neurotoxicant and a suspected immunotoxicant, and benzene was associated with both neurotoxicity in workers exposed to high doses and adverse immune effects. Some authoritative sources noted that some immunotoxicity outcomes associated with benzene exposure may result from hematotoxicity or may occur at levels like those inducing hematotoxicity.

Exposures to higher-priority chemicals were also associated (higher or moderate confidence) with other adverse health outcomes. The most sensitive available risk estimates for these higher-priority chemicals (see Tables S5 and S6) are reported in Box 1.

Given identified research gaps for the higher-priority chemicals, we selected the following chemical × outcome pairs for Phase 2 activities (see Methods): neurotoxicity for acrolein, 2-ethylhexyl acrylate, butyl acrylate, and 2-butoxyethanol; immunotoxicity for butyl acrylate and 2-butoxyethanol; and hepatotoxicity for butyl acrylate. Although vinyl chloride is most likely the chemical of most concern, we did not select it for Phase 2 because a recent ATSDR review was available with conclusions about the selected health outcomes.

##### Potentially related chemicals

ECHA classified 6:2 FTSHA as a presumed toxicant to fertility and development. Two PFAS (6:2 FTSA and 6:2 FTNO) were not associated with reproductive or developmental toxicity, and no conclusions were available for the remaining PFAS (6:2 FTSAS and 6:2 FTSA-PrB). Several agencies (e.g., OEHHA, EPA, ATSDR) have concluded that TCDD can cause adverse reproductive and/or developmental effects and have developed risk estimates based on these effects (EPA Integrated Risk Information System [IRIS] RfD: 7 × 10 − 10 mg/kg/day for oral exposure) (see Table S6). TCDD was also associated with both neurological and immune effects based on available human and animal evidence.

#### Cancer outcomes

##### Primary chemicals

Cancer conclusions were available for approximately two-thirds (12 of 16) of the chemicals (see Fig. 4). Of the eight higher-priority chemicals, five can cause cancer in humans or experimental animals: Benzene and vinyl chloride are IARC and NTP “known human carcinogens” [43–46], acrolein is “probably carcinogenic to humans” based on mechanistic and animal cancer data (IARC 2A), 2-ethylhexyl acrylate is “possibly carcinogenic to humans” (IARC 2B) based on sufficient evidence from studies in experimental animals, and 2-butoxyethanol induces tumors (hemangiosarcoma of the liver in male mice). IARC considered the evidence limited in experimental animals for 2-butoxyethanol and, thus, not classifiable as a carcinogen (Group 3). Butyl acrylate and hydrogen chloride are also classified as Group 3 by IARC based on inadequate evidence from experimental animal and human studies. No conclusions were available for phosgene gas. Among the moderate- and low-priority chemicals, exposure to isobutylene caused tumors in experimental animals. The others were either not likely to cause cancer or had no identifiable conclusions. Cancer types were available for the known human carcinogens: Benzene causes acute myeloid leukemia and other acute nonlymphocytic leukemia and may cause other lymphohematopoietic cancers (chronic myeloid leukemia, chronic lymphocytic leukemia, childhood leukemia, non-Hodgkin lymphoma, and multiple myeloma) and lung cancer; vinyl chloride causes angiosarcomas in the liver.

Taking into account these research gaps, we identified the following chemicals for Phase 2 searches for cancer outcomes: human cancer studies for acrolein, 2-ethylhexyl acrylate, and 2-butoxyethnanol; and human and animal cancer studies for butyl acrylate.

##### Potentially related chemicals

For dioxins, most available cancer outcome data were for TCDD. Both IARC [47] and NTP’s Report on Carcinogens [48] classified TCDD as “carcinogenic to humans” based on sufficient evidence in humans (Group 1). OEHHA [49] provided slope factors for TCDD and a variety of individual chemicals in the class. Inhalation slope factors ranged from 3.9 × 10^1 ^mg/kg/day (for 1,2,3,4,6,7,8,9-Octachlorodibenzo-p-dioxin) to 1.3 × 10^5 ^mg/kg/day (for TCDD and 1,2,3,7,8-Pentachlorodibenzo-p-dioxin) [49]. Data on cancer effects were not available from authoritative sources for the five PFAS or other dioxins.

To increase the utility of our review and because of the limited health information on PFAS and dioxins other than TCDD, we selected 6:2 FTSA, 6:2 FTNO, and dioxins other than TCDD for unrestricted searches (i.e., searches for any health outcome data).

Box 1 Most sensitive available risk assessment values for higher priority chemicals and other noncancer outcomes

**Table:** 

Chemical	Value	Source	Additional information
Hematotoxicity			
Benzene	REL = 0.003 mg/m^3^	OEHHA	Inhalation exposure
	MRL = 0.0003 mg/kg/day	ATSDR	Oral exposure; Based on hematotoxicity/immune effects
2-butoxyethanol	MRL = 0.97 mg/m^3^	ATSDR	Inhalation exposure
	RfD = 0.1 mg/kg/day	EPA IRIS	Oral exposure
Hepatotoxicity			
2-butoxyethanol	MRL = 0.07 mg/kg/day	ATSDR	Oral exposure
Vinyl chloride	RfD = 0.003 mg/kg/day	EPA IRIS	Oral exposure
Respiratory (non-irritating effects)			
Phosgene gas	RfC = 0.0003 mg/m^3^	EPA	Inhalation exposure

### Phase 2

#### Overview

Results from the targeted searches and screenings conducted during Phase 2 are available in Table 1.

**Table 1:** Overview of literature search results for selected chemical-outcome pairs for primary chemicals.

Chemical-outcome category	Phase 1 authoritative source conclusions	Search limit, date completed	Results (*N*)	PECO-relevant studies (N)	Phase 2 new studies (*N*)	Evidence gap
16 Priority Chemicals						
Acrolein-Cancer	Probably carcinogenic to humans (Group 2 A); Inadequate human evidence	Studies published 2020–present, June 22, 2023	183	0	0	Research gap remains (human cancer studies)
Acrolein-Nervous^	No conclusions: Suggestive evidence from Causaly	No date limit, June 20, 2023	228	8 reviews	7 reviews	Systematic review may be warranted
2-butoxyethanol-Cancer^	Not classifiable (Group 3); Limited evidence in experimental animals	No date limit, July 6, 2023	35	1 human	1 human	Research gap remains
2-butoxyethanol-Immune^	No conclusions: Suggestive evidence	No date limit, July 5, 2023	151	1 human 9 animal	1 human 1 animal	Additional studies with focus on functional immunotoxicity are needed
2-butoxyethanol-Nervous^	No conclusions: Suggestive evidence	No date limit, July 5, 2023	112	1 animal 3 human	1 animal	Additional studies designed to assess neurological effects are needed
Butyl acrylate-Cancer	Not classifiable (Group 3); Inadequate human and animal evidence	No date limit, July 6, 2023	65	1 animal	0	Research gap remains
Butyl acrylate-Hepatic	No conclusion: Suggestive evidence	No date limit, July 7, 2023	19	0	0	Research gap remains
Butyl acrylate-Immune	No conclusions: Few studies; Skin sensitization: Category 1	No date limit, July 5, 2023	92	2 animal^a^ 3 human^a^	0	Research gap remains (immune endpoints other than skin sensitization)
Butyl acrylate-Nervous	No conclusions: Few studies	No date limit, July 5, 2023	97	0	0	Research gap remains
2-ethylhexyl acrylate-Cancer	Possibly carcinogenic (Group 2B); Inadequate human evidence	Studies published 2019–present, June 20, 2023	6	0	0	Research gap remains (human cancer studies)
2-ethylhexyl acrylate-Nervous	No conclusions: Few studies	No date limit, June 23, 2023	9	0	0	Research gap remains
Potentially Related Chemicals						
6:2 FTSA-Any^	Skin irritation: Category 1B; Eye irritation: Category 1; No effects for developmental, reproductive, or skin sensitization in animal studies No other conclusions based on limited evidence in experimental animals	Studies published 2020–present, August 10, 2023	45	1 animal 1 human	1 animal 1 human	Research gaps remain
6:2 FTNO-Any	No effects observed on nervous, reproductive, developmental, hematologic, dermal, or ocular outcomes in animal studies	No date limit, August 10, 2023	0	0	0	Research gaps remain
Dioxins other than TCDD-Any^	Conclusions available for TCDD: Known carcinogen (Group 1) Skin and eye irritation: Category 2 May cause adverse effects in nervous, immune, reproductive, developmental, and hepatic systems	Studies published 2018–present, August 22, 2023	61	11	11	Systematic review of dioxin mixtures may be warranted for endometriosis; other research gaps remain
6:2 FTSA-Any^	Skin irritation: Category 1B; Eye irritation: Category 1; No effects for developmental, reproductive, or skin sensitization in animal studies No other conclusions based on limited evidence in experimental animals	Studies published 2020–present, August 10, 2023	45	1 animal 1 human	1 animal 1 human	Research gaps remain

*PECO* Population, Exposure, Comparator, and Outcome.

^Indicates chemical × outcome pairings that are discussed in detail.

^a^All studies on skin sensitization.

##### Primary chemicals

For five searches (acrolein × cancer, 2-ethylhexyl acrylate × cancer, 2-ethylhexyl acrylate × nervous, butyl acrylate × hepatic, butyl acrylate × nervous), no relevant results were identified during title-abstract and/or full-text screening. At least one PECO-relevant reference was identified for each of the remaining six searches (acrolein × nervous [*n* = 8], 2-butoxyethanol × cancer [*n* = 1], 2-butoxyethanol × immune [*n* = 10], 2-butoxyethanol × nervous [*n* = 4], butyl acrylate × cancer [*n* = 1], butyl acrylate × immune [*n* = 5]). Although five PECO-relevant studies were identified for butyl acrylate × immune, all five reported on skin sensitization findings only. As skin sensitization data were considered separately from immune data during Phase 1, the five studies were not considered for further analysis during Phase 2.

For chemical × health outcome pairs with at least one new study that had not been included in authoritative reviews (acrolein × nervous, 2-butoxyethanol × cancer, 2-butoxyethanol × immune, and 2-butoxyethanol × nervous), we examined and summarized all studies identified during Phase 2, alongside some findings from authoritative source reports in Phase 1. Findings for each chemical × health outcome pair are summarized by endpoint in the text (acrolein × nervous and 2-butoxyethanol × cancer) and Table S7 (2-butoxyethanol × immune) and Table S8 (2-butoxyethanol × nervous) below. Figure S3 captures the literature identified and included at each step for these searches. An interactive version of this flow diagram is available on Tableau (see figure caption).

##### Potentially related chemicals

No relevant results were identified during title-abstract and/or full-text screening for 6:2 FTNO × any health outcome. At least one PECO-relevant reference was identified for 6:2 FTSA × any health outcome (*n* = 2) and dioxins other than TCDD × any health outcome (*n* = 11). All studies identified during Phase 2 were summarized, alongside some findings from authoritative source reports in Phase 1. Information on the literature identified and included during these searches is also included in Fig. S1.

#### Selected chemical-outcome: Acrolein-nervous system

Four authoritative reviews—ATSDR [50] (12 studies), ECB [41] (1 study), EPA [51] (1 study), and OEHHA [52] (4 studies)—discussed findings from studies examining acrolein exposures and neurological effects, although none made hazard conclusions. Additionally, outputs from Causaly identified reviews and primary articles that discussed potential associations between acrolein levels and various neurological outcomes, including Alzheimer’s disease, Parkinson’s disease, and strokes.

Our literature scoping search to find published reviews on neurological outcomes identified eight reviews discussing effects and mechanisms associated with acrolein exposure in humans [53–59] and animals [53–56, 58–60], which are summarized below. One review was discussed in ATSDR, 2007 [60], but the remaining seven were not included in any authoritative sources. The four authoritative reports also included 14 primary studies in experimental animals—reporting effects related to neurotransmitter (neuropeptide) depletion, increased brain weight, inflammatory responses, loss of nerve tissue, and nonspecific histopathological effects (in inhalation studies)—and one primary study in humans that reported increased acrolein levels in the brains of Alzheimer’s patients compared to control subjects at autopsy. Most studies discussed in the ATSDR Toxicological Profile examined general toxicity in experimental animals and were not designed to measure neurotoxicity. Detailed results from the eight reviews identified in our literature scoping search are provided in the text below. As our search identified reviews only, a summary table is not provided. Importantly, exposure to acrolein can occur both exogenously and endogenously, as the chemical is a byproduct of lipid peroxidation initiated by oxidative stress [56]. In the eight identified reviews, acrolein is often used as a biomarker for oxidative stress and lipid peroxidation. Therefore, it was often unclear whether neurological effects are related specifically to acrolein or as a biomarker for oxidative stress. The review findings should be considered in this context.

Three other reviews identified during Phase 2 reported on the mechanistic effects of acrolein in nervous tissues [61–63], although mechanistic evidence was not the primary focus of our literature scoping activities. Acrolein was discussed as a highly toxic product of lipid peroxidation that can cross the blood-brain barrier [61]. In vitro and in vivo studies of neuroinflammation and neurodegeneration and acrolein’s role in the development of Alzheimer’s disease, Parkinson’s disease, and spinal cord injury were cited as evidence of its neurotoxic potential. Other reported mechanistic evidence suggests that acrolein induces demyelination of nerves—which impacts nerve conduction—neuronal apoptosis, neurotransmitter alterations, and protein adduct formation. Other reviews reported inhibition of glutamate and glucose uptake in acrolein-exposed neuronal cell cultures [62] and disruption of nerve terminals and subsequent potential for synaptic damage in in vitro studies [63]. These reviews suggest that acrolein, whether endogenous or exogenous, has the potential for neurotoxic effects.

Reviews of human and animal studies discussed the association between acrolein and strokes of varying severity [53, 55–57]; however, the discussion of acrolein’s role differed across reviews. Some reviews assessed acrolein’s role in the development of stroke or brain infarction, whereas others examined acrolein as a byproduct of the oxidative stress induced by stroke or brain infarction and its potential to cause additional neurological damage. Acrolein may be produced endogenously during ischemic stroke [53], and increased endogenous acrolein production has been reported in connection with both severe strokes and silent brain infarctions [57]. A mechanistic study summarized by Muguruma et al. [57] suggested that acrolein elicited a cycling of oxidative stress, resulting in stroke-related neuronal damage, and is a suspected driver of neuronal damage in stroke patients. Plasma levels of protein-conjugated acrolein (along with acrolein-producing enzymes) were shown to be appropriate biomarkers for human stroke [54–56, 58] and silent brain infarctions [55]. Multiple human studies have found dysregulated acrolein metabolism in stroke patients [53].

Findings from reviews of animal studies further support an association between acrolein and stroke, although it was unclear whether animals were dosed in studies cited in the reviews or whether effects were associated with endogenous acrolein. A review of animal studies reported an association between decreasing levels of acrolein and decreased infarction size [53]. A study in mice indicated that, during brain infarction, acrolein is “more strongly involved” in cell damage than reactive oxygen species [55]. Other reviews reported increased levels of acrolein at the site of brain infarction in mouse models [54, 58]. Neuronal damage was also reported in a review of animal studies, including acrolein-induced neuronal damage in pigs and rats, although some studies reported effects of endogenous acrolein only and should be considered accordingly [56]. In an in vitro study, acrolein induced mitochondrial dysfunction leading to neuronal death in HT22 mouse hippocampal cells [56].

Other neurological outcomes have also been considered for their association with acrolein exposure. In humans, significantly increased levels of acrolein were reported in the brains of patients with mild cognitive impairment [55, 57, 59] and cognitive impairment that had progressed to Alzheimer’s disease compared to control subjects [53, 55, 57, 59]. For cases of Parkinson’s disease, both human and animal studies reported that acrolein exposure leads to damage of the substantia nigra [53]. In a mouse model of multiple sclerosis, acrolein was found to be a critical pathological factor in the development of autoimmune encephalomyelitis [53]. Finally, in a review of animals exposed to acrolein via inhalation, alterations in reflex reactions and sensory irritation were reported in guinea pigs [60]. Alterations were a result of pulmonary nerve ending stimulation from the chemical. Decreased respiratory rates were also reported in exposed guinea pigs and in rabbits [60].

##### Evidence gap summary

A *systematic review* critically assessing the body of evidence of acrolein on the nervous system may be warranted for human, animal, and mechanistic studies, with a particular focus on effects from exogenous acrolein exposures.

#### Selected chemical-outcome: 2-Butoxyethanol-Cancer

IARC [64] concluded that 2-butoxyethanol was not classifiable based on its carcinogenicity to humans because of limited evidence from studies in experimental animals and inadequate evidence from studies in humans. One human study with limited information on 2-butoxyethanol exposure was identified. The limited evidence of carcinogenicity in experimental animals was from a study of 2-butoxyethanol inhalation in rats and mice published in NTP Technical Report 484 (NTP TR-484) [65]. Additional studies published after the IARC Monograph were not identified for the other two authoritative reviews (EPA IRIS [66] and OEHHA [67]). In mice, NTP [65] concluded there was some evidence of carcinogenicity in males based on liver hemangiosarcoma and in females based on forestomach squamous cell papilloma or carcinoma (mainly papilloma). For rats, there was equivocal evidence of carcinogenicity in females based on benign and malignant pheochromocytoma (mostly benign) of the adrenal medulla and no evidence of carcinogenicity in males.

Our literature scoping activities to find cancer studies in the literature identified one primary article published after the authoritative reviews that described cancer effects associated with 2-butoxyethanol exposure in humans [68]. Rodrigues et al. [68], an occupational nested case-control study of workers at three semiconductor and storage device manufacturing facilities, evaluated the association between exposure to 31 known or possible carcinogens, including 2-butoxyethanol, and central nervous system (CNS) cancers. The study reported significant exposure-response associations (p_trend_ < 0.01) with increased odds ratios (ORs) for CNS cancer incidence in all quartiles (vs. Quartile 1) at two of the three module manufacturing work sites assessed; ORs were <1 at the third site [68]. Statistically significant positive trends were reported for several chemicals that were present in the module manufacturing work sites in addition to 2-butoxyethanol.

##### Evidence gap summary

A *research gap remains* for additional primary studies of the carcinogenicity of 2-butoxyethanol, particularly for studies of effects in human populations.

#### Selected chemical-outcome: 2-Butoxyethanol-Immune

Three authoritative reviews—ATSDR [69] (24 studies), EPA [66] (5 studies), and OEHHA [67] (5 studies)—discussed findings from studies examining 2-butoxyethanol exposures and immune effects, although none made hazard conclusions. Despite the lack of hazard conclusions, each review provided summaries of immunological findings from several studies in animals and humans. Our literature scoping activities to find immunological studies in the published literature identified 10 discussing immune-related outcomes associated with 2-butoxyethanol exposure, including one primary article in a human population [70] and nine primary articles in experimental rodents (rats and mice) [71–79]. Two of these studies were not included in the collective authoritative source reports [70, 79]. Animal toxicology studies summarized in ATSDR [69] identified immunological effects in studies designed to assess general toxicity (and thus, not all were identified by our immune-targeted literature searches). These studies largely reported effects in lymphoreticular organs (e.g., thymus weight changes, thymus histopathology), whereas reviews from EPA IRIS [66] and OEHHA [80] were primarily of studies designed to evaluate immunotoxicity and found evidence of immunomodulatory effects (see study summaries in Table S7).

We reviewed studies identified in our literature scoping activities and the summaries of studies from authoritative reviews (that were not identified in our literature searches). In its discussion of immunotoxic effects, ATSDR [69] discussed immune and lymphoreticular effects separately and noted that some impacts to lymphoreticular organs can be attributed to hematotoxicity rather than to immunotoxicity (noting that there is overlap between the two, as leukocytes can be classified as part of both systems). These studies were not considered in this report’s summary of immune effects. Additionally, we did not include four studies from the reports that examined skin sensitization in humans [81, 82], guinea pigs [83], and mice [84] because our review of authoritative sources discussed skin sensitization as a separate health outcome category (see Fig. 3). Detailed summaries from 16 studies reporting immunotoxicity effects (functional and observational findings) are available in Table S7. Primarily based on findings from one study [77], OEHHA concluded that immunomodulation effects were observed in animals for autoimmune response, certain types of T-cell-mediated effects, and natural killer (NK) cell activities, but not for B-cells. Few other studies examining lymphocyte responses were available, limiting the ability to draw broader conclusions about these findings.

##### Evidence gap summary

*Additional studies focusing on functional immunotoxicity* are needed to provide more specific information on the direct effects of 2-butoxyethanol on the immune system. Available studies may not be adequate for a systematic review of potential immunotoxicity.

#### Selected chemical-outcome: 2-Butoxyethanol-nervous system

Three authoritative reviews—ATSDR [69] (24 studies), EPA [66] (9 studies), and OEHHA [67] (5 studies)—discussed studies of neurological effects following exposure to 2-butoxyethanol. ATSDR’s review of the association between exposure to 2-butoxyethanol and neurological effects included many general toxicology studies of animals exposed by oral, dermal, and inhalation routes and several case reports. Reviews from the EPA and OEHHA were limited to case reports. Our literature scoping activities to find neurological studies in the published literature identified one primary article in rats that was not included in any authoritative reviews [85] and three human case reports included in the reviews [86–88].

Below, we review the combined body of relevant literature (human and animal studies), which consists of animal evidence reported by ATSDR, an additional animal study identified in our literature scoping review, and the collective case reports/series identified in our literature search and discussed in authoritative reviews.

ATSDR [69] concluded that exposure to high doses in experimental animals can cause nervous system effects (e.g., physical weakness, unsteadiness, drowsiness, prostration, abnormal eye movement, convulsions). Studies also reported clinical observations prior to death (e.g., convulsions, nystagmus, moderate to marked inactivity, ataxia). While ATSDR classifies many of these as clinical signs of neurotoxicity, these could also be attributed to other causes. Thus, we did not include 14 animal studies reporting on these symptoms and clinical observations. Brain weight findings were also not included in this review, as ATSDR reported results from only two studies, and it was unclear whether this endpoint was measured in other general toxicity studies. Other observed effects in case reports and animal studies that may be more reflective of impacts to neurological function are included, such as cases of coma following exposure, severe CNS depression, and effects related to the motor and vestibular systems (e.g., impacts to coordination, loss of equilibrium), sensory systems (e.g., disturbed taste), and neurological histopathology (e.g., histopathological changes to the brain and nerves).

Detailed summaries of neurotoxicity findings from 17 studies (8 case reports/series, 9 primary studies) are available in Table S8.

##### Evidence gap summary

*Additional studies are needed*, including human epidemiological and additional animal studies specifically designed to assess neurological effects following exposure. Inadequate database for a systematic review of potential nervous system effects.

#### Selected chemical-outcome: 6:2 FTSA any health outcome

No reports from authoritative sources that discussed health effects associated with 6:2 FTSA exposure were identified during Phase 1. Two primary studies (one human study [89] and one animal study [90]) were identified during Phase 2 that examined the health effects associated with 6:2 FTSA. Studies assessed reproductive and developmental effects and immune effects.

##### Reproductive and developmental effects

Both studies assessed reproductive and developmental effects associated with 6:2 FTSA exposure [89, 90]. In humans, a case-control study in Hangzhou, China of 82 preeclamptic pregnant women and 169 healthy control subjects measured 6:2 FTSA in maternal serum prior to delivery [89]. The study observed no significant associations between maternal serum 6:2 FTSA levels and odds of preeclampsia or odds of low birth weight in infants. A study of male and female white-footed field mice exposed to 6:2 FTSA by oral gavage for 112 days (from 4 weeks premating to ≥4 weeks postmating) observed no associations between 6:2 FTSA exposure and reproductive and fertility endpoints in the exposed mice or developmental endpoints in their offspring [90]. Reproductive and developmental measures included the number of mating pairs, the number of pregnant animals, total litter loss, proportion of stillbirths, live litter size, total litter size, male sperm parameters, male and female sex hormone levels, and pup weights.

##### Immune effects

One primary study in an ecological model assessed the association between 6:2 FTSA exposure and immune endpoints [90]. Male and female white-footed field mice were exposed to 6:2 FTSA by oral gavage for 112 days (from 4 weeks premating to ≥4 weeks postmating). Researchers observed significantly decreased plaque-forming cell counts in both males and females, significantly increased spleen weights in males only, and no changes in thymus organ weight or histopathology of the thymus or spleen in either sex [90]. A benchmark dose (BMD) was derived using data for decreased plaque-forming cell counts (BMD for males = 4.06 mg/kg/day; for females = 3.72 mg/kg/day). The lower 95% confidence limits (BMDLs) were 2.63 mg/kg/day (males) and 2.26 mg/kg/day (females).

##### Evidence gap summary

A *research gap remains* for primary studies of health hazards associated with 6:2 FTSA exposure in humans and animals. Few studies were identified.

#### Selected chemical-outcome: Dioxin mixtures-any health outcome

Authoritative reviews identified in Phase 1 mostly discussed findings and drew conclusions based on studies of TCDD exposures, as little information was available for other chemicals in the class. During Phase 2, we identified 11 systematic reviews and meta-analyses reporting health effects associated with exposures to dioxin mixtures (including TCDD in some cases) or dioxins other than TCDD, including polychlorinated dibenzo-p-dioxins and dibenzofurans (PCDDs/Fs) [91–101]. One review by Wallace et al. [100] reported lower sleep quality associated with exposure to dioxin mixtures.

##### Reproductive and developmental effects

Five systematic reviews examined associations between dioxins and reproductive or developmental outcomes in humans and animals, including endometriosis [93, 97, 99], time to pregnancy [96], and in utero anogenital distance in humans [98].

A meta-analysis of 10 case-control studies in humans found significantly increased odds of endometriosis associated with exposure to dioxin mixtures and TCDD (OR = 1.65, 95% confidence interval [CI]: 1.14, 2.39) [93], with supporting suggestive epidemiological evidence reported in another systematic review of dioxins and dioxin-like chemical (DLC) exposures [99]. Findings from animal studies of TCDD and DLCs were largely consistent with those from human studies, reporting associations with endometriosis and endometriotic lesions following exposures [97]. Results from the review of animal studies are consistent with endometriosis findings in the animal studies included in authoritative reviews.

Results were mixed across eight studies reviewed by Kahn et al. [96] and two additional studies reviewed by Nelson et al. [98] examining changes in time to pregnancy and in utero anogenital distance in humans [96, 98]. Exposure assessment was heterogeneous across the eight studies, with some studies assessing TCDD or PCDDs/Fs and others assessing dioxin-like polychlorinated biphenyl (PCB) congeners.

##### Cancer effects

Three systematic reviews examined associations between dioxins and cancer outcomes (soft tissue sarcoma and breast cancer) in humans [92, 95, 101]. A meta-analysis of four cohorts reported a significant association between occupational exposure to dioxin mixtures, including exposure to TCDD in three cohorts, and soft tissue sarcoma mortality (pooled standardized mortality ratio (SMR) (95% CI) = 2.56 (1.60, 4.10)) [95]. This finding was supported by a case-control study from another systematic review, which observed increased risk of soft tissue sarcoma with increased TCDD and PCDD/F exposure levels [92]. These findings are consistent with the IARC conclusion that there is limited evidence from studies in humans of an association between TCDD and soft tissue sarcoma [47]. Across the two reviews, two studies reported on exposures to dioxins other than TCDD, and both studies reported positive associations with soft tissue sarcoma.

A systematic review of environmental exposures and breast cancer identified only one study on dioxin exposure: a case-control nested in a French cohort study, which found increased odds of early onset breast cancer for the second quintile of dioxin exposure. ORs were imprecise for higher dioxin exposure, and no exposure-response trends were observed [101].

##### Neurological effects

Two systematic reviews of studies reporting on attention deficit hyperactivity disorder (ADHD) in children and air pollution exposure [91], as well as autism in children and adolescents and exposure to endocrine disrupting chemicals [94], included studies of exposure to dioxins. Results for ADHD were mixed across two cohort studies, with both positive and negative associations observed [91]. One study was found that examined child autism following prenatal exposures to DLCs and reported decreased social responsiveness, particularly in girls [94]. No associations were reported for other autistic traits.

#### Evidence gap summary

*Systematic reviews* critically assessing the bodies of available human, animal, and mechanistic evidence on exposures to dioxin mixtures may be warranted for endometriosis. Additional studies examining soft tissue sarcoma and neurological effects are needed, as findings were mixed (soft tissue sarcoma) or few studies were identified (neurological effects). A research gap remains for health outcome categories other than reproductive/developmental, neurological, and cancer.

## Discussion

This review applied a rapid, phased approach to scope available health hazard information for chemicals released or suspected to be released during the East Palestine, Ohio train derailment. We considered eight chemicals (acrolein, butyl acrylate, 2-butoxyethanol, 2-ethylhexyl acrylate, benzene, hydrogen chloride, phosgene gas, and vinyl chloride) in higher-priority categories (i.e., highest or high) for identification of key health effect data gaps based on the potential for exposure.

Our review found that irritant was the most established health outcome; all eight higher-priority chemicals were identified as skin and eye irritants and seven as respiratory irritants. These findings are consistent with some acute symptoms reported by those affected following the train derailment in East Palestine. Five of the eight higher-priority chemicals were human (benzene and vinyl chloride) or animal carcinogens (butyl acrylate, 2-butoxyethanol, and 2-ethylhexyl acrylate). Studies in humans remained a research gap, as few additional human studies were identified in Phase 2 searches. Reproductive and developmental outcomes were the most studied noncancer outcomes. Few conclusions were available from authoritative sources for other noncancer outcomes, including neurological and immunological effects. Benzene and vinyl chloride were deemed harmful to reproductive and/or developmental systems and are associated with neurological and immunological effects. Four (acrolein, 2-butoxyethanol, butyl acrylate, and 2-ethylhexyl acrylate) were of low or no concern for reproductive or developmental effects. Our review reveals key health hazard evidence gaps for neurological effects following exposures to acrolein, butyl acrylate, 2-butoxyethanol, and 2-ethylhexyl acrylate. We also recommend conducting a systematic review to identify primary studies for neurological effects associated with acrolein exposure, as our report focused on reviews only. Finally, studies assessing immunotoxicity following 2-butoxyethanol and butyl acrylate exposure should be pursued, as many identified studies reported observational immune findings from studies of general toxicity. Additional research may be needed before a systematic review is pursued.

Among the potentially related chemicals, conclusions were not available for most health outcome categories for the five PFAS or dioxins other than TCDD. Additional studies examining the health effects associated with 6:2 FTSA and dioxin mixtures are needed. A systematic review may also be warranted to identify primary literature on dioxin mixtures and the potential association with endometriosis.

While we aimed to conduct a robust and transparent assessment of the available literature on health effects following exposure to the chemicals of interest, this literature scoping review has some limitations.

First, this review did not incorporate measures to determine the true exposure or risk experienced by East Palestine residents. As stated above, the focus of our review was on chemical human hazard identification, a necessary first step in environmental risk assessment, and vital for informing decision-making in scenarios such as the East Palestine train derailment. However, we want to emphasize that the process for identifying human health hazards should not be conflated with an automatic risk to human health. Determining that a chemical poses a potential hazard to human health does not establish that it will cause those hazards for individuals. Many factors that vary by the individual, including exposure-related factors (e.g., characteristics of exposure, co-exposures, and evacuation activities) and individual susceptibilities, influence an individual’s risk of adverse health effects. Public health decision makers should take into account that health hazard conclusions for single chemicals may underestimate the risk from a disaster to a specific community due to insufficient information on exposure and population susceptibility. The complete exposure scenario of relevant exposure pathways is unknown, and, in some cases, definitive information on the chemicals released was difficult to obtain. A study published after the completion of our literature scoping found that people may have been exposed via multiple sources or pathways, including indoor air, ambient air, surface water, and clean-up activities for months following the derailment [102]. Risk assessment, considering evidence-based hazard and exposure information, would then guide response activities. As such, caution should be given when interpreting and communicating results from hazard assessments. Certain subgroups–such as those living below the poverty level (~10%) [103], children, with underlying health conditions, or those exposed to higher exposure based on where they work or live–in East Palestine may have a higher risk for adverse health effects from the disaster. According to the EPA EJScreen [104], East Palestine, OH, ranks high in the percentile (80 to 90%) for low life expectancy, heart disease, and, to a lesser degree, asthma (50 to 80%). Lower socioeconomic status (low income, unemployment rate, and less than a high school education) is a concern in some of the surrounding communities. Additionally, community members were likely exposed to mixtures of chemicals that may present a greater risk than that reflected by hazard conclusions for single chemicals. Although EPA did not report PM_2.5_ levels in their monitoring dashboards [105], they were likely increased from the fire. Elevated PM_2.5_ is associated with increased risk for adverse health outcomes such as respiratory effects, cardiovascular disease [106], and lung cancer. In addition to the derailment chemicals, East Palestine ranks high for several environmental burden indicators, including toxic releases to the air, ozone, lead paint, underground storage tanks, wastewater discharges, and drinking water non-compliance, which may all play a role in adverse health effects.

Second, the heterogeneity of reviews from authoritative sources in Phase 1 should be acknowledged. Reports from authoritative sources did not always provide clear interpretations of the available data, and, in some cases, different reports provided conflicting interpretations of largely similar databases. These variations made it difficult to synthesize across some authoritative conclusions. Some reviews emphasized animal studies designed to assess toxicities to a specific organ system, such as neurotoxicity or immunotoxicity studies, whereas other authoritative reviews also integrated relevant endpoints from animal studies of general toxicity. Additionally, some reports (e.g., reports from ECHA) presented hazard data but drew risk-based conclusions, whereas others made hazard conclusions only. Not all authoritative sources incorporate mechanistic data, such as biological effects and key characteristics of carcinogens [107], cardiovascular toxicants [108], immune toxicants [109], reproductive toxicants [110, 111], or endocrine-disrupting chemicals [112], into their hazard conclusions. Finally, the language used to describe hazards and risks is not harmonized across sources. While some provide clear hazard conclusions with specific codified language, others summarize health effects data without obvious conclusive statements. In some cases, the variable language across sources made it difficult to discern hazard conclusions. Future reviews that follow this approach should consider the authoritative sources most informative for their topic and how findings across sources can be synthesized accurately and consistently.

Third, industrial chemicals, including those in this review, often have publicly available toxicological data that are not accessible in research journal articles or databases such as PubMed. Our search and synthesis of the information in the gray literature relied upon interpretations from authoritative sources (i.e., conclusions in reviews and reports from entities such as ATSDR, ECHA, EPA IRIS, and CalEPA). While ECHA reports and classification and labeling documents were reviewed to identify as much data as possible, some information in the gray literature may not have been identified.

Fourth, there may have been some published literature that was not identified during our searches. For example, some measures common in most animal studies (e.g., brain weight) may not be reported in the title and abstract of a study. If relevant endpoints were not discussed in the title and abstract, these studies were not identified in our review process. Additionally, two searches identified review articles only (acrolein and neurological effects, and dioxins other than TCDD and any health effects). As such, the available primary literature is not summarized but may provide useful information on these effects.

Fifth, because our review was associated with the NASEM workshop, the assessment was initiated several weeks after the train derailment, and the data were not released in phases. Figure 1 provides a timeline estimate of data were released in phases, which would have informed shorter-term research responses. For example, in a phased scoping review, comprehensive information on known and unknown health effects of multiple chemicals could be reported within weeks of a disaster or less for subsets of higher-priority chemicals. Nevertheless, because many non-cancer and cancer outcomes may have longer latency periods, our report provides meaningful and comprehensive knowledge to inform the affected community, health providers, and researchers.

Finally, our assessment of the available literature did not include an evaluation of study quality and risk of bias, which are traditionally conducted during systematic reviews, especially those of single chemicals, and without time restrictions. In addition to the potential for bias, studies finding no effects may not be sensitive to detect a true effect. While efforts were made to consider the available data in a standardized way, our results should be interpreted with appropriate caution.

Our review also had several strengths. This fit-for-purpose rapid review method can be used to search and review the literature on many chemicals simultaneously, allows for a broad understanding of hazard, and enables decision-makers to expedite the gathering of hazard information for a variety of chemicals on a compressed timeline. Additionally, a phased approach facilitates the delivery of different review products based on project needs and timelines. While we aimed to provide information responsive to the health concerns expressed by the affected community and, thus, focused on this specific incident, these chemicals are used in a variety of contexts and enter the environment regularly. Moreover, we believe this rapid, phased approach can be leveraged broadly for environmental emergencies, when the need for health hazard information is urgent, and time may be limited. According to the chemical incident database compiled by the Coalition to Prevent Chemical Disasters, there were 52 unique incidents across 20 states between 2021 and 2023 that involved at least one of the chemicals of interest in this review [1]. Therefore, the summarized hazard information in this review can also be used beyond the context of the East Palestine train derailment, including at facilities using these chemicals as part of EPA’s Risk Management Program.

This rapid review summarizes the available health hazard data for 22 chemicals released or potentially released during the East Palestine, Ohio train derailment and subsequent controlled burn and identifies known and suspected hazards and research gaps.

Our evaluation serves to inform health officials who communicate with the affected individuals and organizations in the East Palestine community, to foster further efforts to better characterize health hazards following environmental exposures, and to protect the general population from such hazardous health effects in the future. An infographic was developed with the aim to communicate our approach and review findings to affected community members (Supplemental Fig. S4). Still, findings from rapid reviews should be interpreted with caution. Importantly, this review solely summarized evidence for hazard identification and, thus, does not integrate data on the likelihood of exposure to the chemicals of interest or provide conclusions related to risk. These hazard-related findings should not be used to contextualize the potential for exposure following the derailment. In addition, the absence of health effect data on chemicals should not be construed as implying that they are not associated with specific adverse outcomes or are safe. Public health decision-makers often need to take action to protect people in the absence of knowledge. The review also helps to inform investigators conducting ongoing studies of the health of the community and surrounding areas. Despite limitations, this rapid review approach can be adapted to expedite the identification of available hazard evidence to inform environmental emergency decision-making and research prioritization.

## Supplementary information

Supplementary information

Supplementary information

Supplementary information

## Data Availability

All data generated or analyzed during this study are included in this published article and its supplementary information files.
