# Supplementary material for: Identifying the known and unknown health hazard information for chemical disasters: a phased scoping review of the East Palestine, Ohio train derailment
Source: J Expo Sci Environ Epidemiol. 2025 Sep 12;35(6):888–906. doi: 10.1038/s41370-025-00803-0 (PMC12583197; doi:10.1038/s41370-025-00803-0)
Supplement: Supplementary file 1 — Supplementary information [file 41370_2025_803_MOESM1_ESM.docx]

**Identifying the known and unknown health hazard information for chemical disasters: A phased scoping review of the East Palestine, Ohio train derailment**

Ruth M. Lunn^a^*, Meredith Clemons^b^, Robyn Blain^b^, Somdat Mahabir^c^, Suril S. Mehta^a^, Andrew A. Rooney^a^, Anisha Singh^a^, Stephanie Smith-Roe^a^, Kyla W. Taylor^a^, Wren Tracy^b^, Maricruz Zarco^d^, Suzanne E. Fenton^a,e,^^[[1]](#footnote-2)^

^a^Division of Translational Toxicology, National Institute of Environmental Health Sciences, Research Triangle Park, North Carolina 27709, USA

^b^ICF, Reston, Virginia 20190, USA

^c^Epidemiology and Genomics Research Program, Division of Cancer Control and Population Sciences National Cancer Institute, Rockville, Maryland 20892, USA

^d^Formerly employed at ICF, Reston, Virginia 20190, USA

^e^Center for Human Health and the Environment, NC State University, Raleigh, NC 27695 USA

Ruth M. Lunn and Meredith Clemons contributed equally to this work.

*Corresponding Author:

Ruth M. Lunn

[lunn@niehs.nih.gov](mailto:lunn@niehs.nih.gov)

National Institute of Environmental Health Sciences

P.O. Box 12233, MD K2-14, Research Triangle Park, NC 27709

984-287-3155

**Supplemental Materials**

[Phase 2 Search Strings 3](#_Toc201827299)

[Chemical Strings 3](#_Toc201827300)

[Acrolein 3](#_Toc201827301)

[2-Ethylhexyl Acrylate 3](#_Toc201827302)

[Butyl Acrylate 3](#_Toc201827303)

[2-Butoxyethanol 3](#_Toc201827304)

[6:2 FTNO 3](#_Toc201827305)

[6:2 FTSA 4](#_Toc201827306)

[Dioxins 4](#_Toc201827307)

[Health Outcome Strings 4](#_Toc201827308)

[Cancer 4](#_Toc201827309)

[Neurological 5](#_Toc201827310)

[Liver/Hepatic 7](#_Toc201827311)

[Immunological 7](#_Toc201827312)

[Evidence Stream Strings 8](#_Toc201827313)

[Human and Epidemiological Terms 8](#_Toc201827314)

[Animal 8](#_Toc201827315)

[Study Type 9](#_Toc201827316)

[Reviews 9](#_Toc201827317)

[Supplemental Tables 10](#_Toc201827318)

[Table S1 Production Volumes of East Palestine Chemicals of Interest from EPA’s 2020 Chemical Data Reporting [2] 10](#_Toc201827319)

[Table S2 Population, Exposure, Comparator, and Outcome/Evidence Stream, Exposure, Comparator, and Outcome Statements 12](#_Toc201827320)

[Table S3 Health Outcome Concepts 15](#_Toc201827321)

[Table S4 Phase 1 Authoritative Sources with Available Data by Chemical 16](#_Toc201827322)

[Table S5 Detailed Findings from Phase 1 Authoritative Source Reviews for 16 Primary East Palestine Chemicals of Interest 19](#_Toc201827323)

[Table S6 Detailed Findings from Phase 1 Authoritative Source Reviews for Potentially Related Chemicals 23](#_Toc201827324)

[Table S7. Summary of 2-Butoxyethanol Immune Studies 25](#_Toc201827325)

[Table S8. Summary of 2-Butoxyethanol Nervous Studies 27](#_Toc201827326)

[Supplemental Figures 29](#_Toc201827327)

[Figure S1. Phased Approach Schematic 29](#_Toc201827328)

[Figure S2. Categorization of Authoritative Source Findings by Confidence or Severity 30](#_Toc201827329)

[Figure S3. Summary of Phase 2 Literature Search and Screening for Select Chemicals 31](#_Toc201827330)

[Figure S4. Infographic of the Rapid Review of East Palestine Chemicals 32](#_Toc201827331)

[References 33](#_Toc201827332)

Phase 2 Search Strings

Chemical Strings

Acrolein

("107-02-8"[rn] OR "2-Propenal"[tiab] OR "Acrolein"[tiab] OR "Prop-2-enal"[tiab] OR "2-Propen-1-al"[tiab] OR "2-Propen-1-one"[tiab] OR "Acroleina"[tiab] OR "Acrylaldehyd"[tiab] OR "Acrylaldehyde"[tiab] OR "Acrylic aldehyde"[tiab] OR "Allyl aldehyde"[tiab] OR "Aqualin"[tiab] OR "Magnacide B"[tiab] OR "Magnacide H"[tiab] OR "NSC 8819"[tiab] OR "Prop-2-en-1-al"[tiab] OR "Propenal"[tiab] OR "UN 1092"[tiab] OR "DTXSID5020023"[tiab] OR "Acrolein"[mh])

2-Ethylhexyl Acrylate

("103-11-7"[rn] OR "2-Ethylhexyl acrylate"[tiab] OR "2-Ethylhexyl prop-2-enoate"[tiab] OR "2-Propenoic acid, 2-ethylhexyl ester"[tiab] OR "EC No.: 203-080-7"[tiab] OR "2-Ethylhexyl 2-propenoate"[tiab] OR "2-Ethylhexylacrylat"[tiab] OR "2-Propenoic acid 2-ethylhexyl ester"[tiab] OR "2-Propenoic acid, 2-ethylhexyl ester"[tiab] OR "acrilato de 2-etilhexilo"[tiab] OR "ACRYLATE, 2-ETHYLHEXYL"[tiab] OR "Acrylic acid, 2-ethylhexyl ester"[tiab] OR "ACRYLSAEURE-(2-AETHYLHEXYL)-ESTER"[tiab] OR "NSC 4803"[tiab] OR "Octyl acrylate"[tiab] OR "PROP-2-ENOATE, 2-ETHYLHEXYL"[tiab] OR "DTXSID9025297"[tiab])

Butyl Acrylate

("Butyl acrylate"[tiab] OR "141-32-2"[rn] OR "DTXSID6024676"[tiab] OR "141-32-2"[tiab] OR "2-Propenoic acid, butyl ester"[tiab] OR "ACRYLATE, BUTYL"[tiab] OR "ACRYLIC ACID, BUTYL ESTER"[tiab] OR "Butan-1-yl acrylate"[tiab] OR "Butyl acrylate"[tiab] OR "Butyl prop-2-enoate"[tiab] OR "Butyl propenoate"[tiab] OR "n-Butyl acrylate"[tiab] OR "PROP-2-ENOATE, BUTYL"[tiab] OR "UN 2348 (DOT)"[tiab] OR "2-Propenoic acid butyl ester"[tiab] OR "2-Propenoic acid, n-butyl ester"[tiab] OR "ACRYLATE, BUTYL"[tiab] OR "Acrylic acid butyl ester"[tiab] OR "ACRYLIC ACID, BUTYL ESTER"[tiab] OR "Acrylic acid n-butyl ester"[tiab] OR "ACRYLSAEURE-BUTYLESTER"[tiab] OR "Butyl 2-propenoate"[tiab] OR "Butylacrylat"[tiab] OR "NSC 5163"[tiab] OR "PROP-2-ENOATE, BUTYL"[tiab] OR "UN 2348"[tiab] OR "n-butyl acrylate"[Supplementary Concept])

2-Butoxyethanol

("111-76-2"[rn] OR "2-butoxietanol"[tiab] OR "2-Butoxyethan-1-ol"[tiab] OR "2-Butoxyethanol"[tiab] OR "EGBE"[tiab] OR "Ethanol, 2-butoxy-"[tiab] OR "Ethylene glycol monobutyl ether"[tiab] OR "2-butoxietanol"[tiab] OR "2-Butoxy-1-ethanol"[tiab] OR "2-BUTOXY ETHANOL"[tiab] OR "2-butoxyethanol m"[tiab] OR "2-n-Butoxyethanol"[tiab] OR "3-Oxa-1-heptanol"[tiab] OR "AETHYLENGLYKOL-MONOBUTYLAETHER"[tiab] OR "Bikanol B 1"[tiab] OR "Buchiseru"[tiab] OR "Butoxyethanol"[tiab] OR "Butyl Cellosolve"[tiab] OR "Butyl Cellu-Sol"[tiab] OR "BUTYL GLYCOL"[tiab] OR "Butyl Glysolv"[tiab] OR "Butyl icinol"[tiab] OR "Butyl monoether glycol"[tiab] OR "Butyl Oxitol"[tiab] OR "Chimec NR"[tiab] OR "DB solvent"[tiab] OR "Dowanol EB"[tiab] OR "Eastman EB"[tiab] OR "Ektasolve EB"[tiab] OR "Ethanol, 2-butoxy-"[tiab] OR "ETHYLENE GLYCOL BUTYL ETHER"[tiab] OR "Ethylene glycol mono-n-butyl ether"[tiab] OR "Ethylene glycol n-butyl ether"[tiab] OR "Gafcol EB"[tiab] OR "Glycol butyl ether"[tiab] OR "Glycol EB"[tiab] OR "Glycol monobutyl ether"[tiab] OR "Hydroxyethyl butyl ether"[tiab] OR "K Foam Lo"[tiab] OR "Mearcell 3532"[tiab] OR "Minex BDH"[tiab] OR "Monobutyl glycol ether"[tiab] OR "n-Butoxyethanol"[tiab] OR "n-Butyl cellosolve"[tiab] OR "NSC 60759"[tiab] OR "O-Butyl ethylene glycol"[tiab] OR "Poly-Solv EB"[tiab] OR "UN 2369"[tiab] OR "β-Butoxyethanol"[tiab])

6:2 FTNO

("1-Octanesulfonamide, N-[3-(dimethylamino)propyl]-3,3,4,4,5,5,6,6,7,7,8,8,8-tridecafluoro-, N-oxide"[tiab] OR "1-Octanesulfonamide, N-[3-(dimethylnitroryl)propyl]-3,3,4,4,5,5,6,6,7,7,8,8,8-tridecafluoro-"[tiab] OR "1-Octanesulfonamide, N-[3-(dimethyloxidoamino)propyl]-3,3,4,4,5,5,6,6,7,7,8,8,8-tridecafluoro-"[tiab] OR "80475-32-7"[rn] OR "N-[3-(dimethylamino)propyl]-3,3,4,4,5,5,6,6,7,7,8,8,8-tridecafluorooctanesulphonamide N-oxide"[tiab] OR "N-[3-(Dimethyloxidoamino)propyl]-3,3,4,4,5,5,6,6,7,7,8,8,8-tridecafluoro-1-octanesulfonamide"[tiab] OR "N,N-Dimethyl-3-[(3,3,4,4,5,5,6,6,7,7,8,8,8-tridecafluorooctane-1-sulfonyl)amino]propan-1-amine N-oxide"[tiab] OR "N,N-Dimethyl-3-{[(3,3,4,4`,5`,5`,6`,6`,7`,7`,8`,8`,8-tridecafluorooctyl)sulfonyl]amino}-1-propanamine N-oxide"[tiab] OR "N,N-Dimethyl-3-((perfluorohexyl)ethylsulfonyl)aminopropanamine N-oxide"[tiab] OR "6:2 FTNO"[tiab] OR "6:2 fluorotelomer sulfonamide amine oxide"[tiab])

6:2 FTSA

("1H,1H,2H,2H-perfluorooctanesulfonic acid"[tiab] OR "1H,1H,2H,2H-Perfluorooctanesulfonic acid"[tiab] OR "1-Octanesulfonic acid, 3,3,4,4,5,5,6,6,7,7,8,8,8-tridecafluoro-"[tiab] OR "27619-97-2"[rn] OR "2-(Perfluorohexyl)ethane-1-sulfonic acid"[tiab] OR "2-(Perfluorohexyl)ethanesulfonic acid"[tiab] OR "3,3,4,4,5,5,6,6,7,7,8,8,8-Tridecafluoro-1-octanesulfonic acid"[tiab] OR "3,3,4,4,5,5,6,6,7,7,8,8,8-Tridecafluoroctansulfonsaure"[tiab] OR "3,3,4,4,5,5,6,6,7,7,8,8,8-Tridecafluorooctane-1-sulfonic acid"[tiab] OR "3,3,4,4,5,5,6,6,7,7,8,8,8-Tridecafluorooctanesulfonic acid"[tiab] OR "3,3,4,4,5,5,6,6,7,7,8,8,8-tridecafluorooctanesulphonic acid"[tiab] OR "6:2 Fluorotelomer sulfonic acid"[tiab] OR "Acide 3,3,4,4,5,5,6,6,7,7,8,8,8-tridecafluorooctanesulfonique"[tiab] OR "Fluorotelomer sulfonic acid 6:2"[tiab] OR "6:2 FtS"[tiab] OR "6:2 FTS"[tiab] OR "6:2 FTSA"[tiab] OR ("fluorotelomer sulfonic acids" [Supplementary Concept] AND 6:2[tiab]))

Dioxins

("Dioxins"[Mesh] OR "Dioxins and Dioxin-like Compounds"[Mesh] OR Dioxin*)

Health Outcome Strings

Note that health outcome strings are based on SWIFT-Review search filters [[1](#_ENREF_1)].

Cancer

(acanthoma*[tiab] OR acrochord*[tiab] OR acrospiroma*[tiab] OR adamantinoma*[tiab] OR adenoacanthoma*[tiab] OR adenoameloblast*[tiab] OR adenocarcin*[tiab] OR adenofibrom*[tiab] OR adenol*[tiab] OR adenom*[tiab] OR "adenosquamous"[tiab] OR ameloblast*[tiab] OR androblast*[tiab] OR angiofib*[tiab] OR angiog*[tiab] OR angiok*[tiab] OR angiol*[tiab] OR angiom*[tiab] OR "angiomatosis"[tiab] OR "angiomatosis"[mh] OR "angiosarc*"[tiab] OR "antibodies, neoplasm"[tiab] OR "antibodies, neoplasm"[mh] OR "antigens, neoplasm"[tiab] OR "antigens, neoplasm"[mh] OR apudom*[tiab] OR argentaffin*[tiab] OR arrhenoblast*[tiab] OR astroblast*[tiab] OR astrocytom*[tiab] OR astrogliom*[tiab] OR "atypia"[tiab] OR "baltoma"[tiab] OR "barrett esophagus"[tiab] OR "barrett esophagus"[mh] OR blastom*[tiab] OR "cancer"[tiab] OR cancero*[tiab] OR "cancers"[tiab] OR carcinog*[tiab] OR "carcinogenicity tests"[tiab] OR "carcinogenicity tests"[mh] OR "carcinogens"[tiab] OR "carcinogens"[mh] OR carcinoid*[tiab] OR carcinom*[tiab] OR carcinos*[tiab] OR cavernom*[tiab] OR "cell line, tumor"[tiab] OR "cell line, tumor"[mh] OR cementom*[tiab] OR cerumin*[tiab] OR chloroma*[tiab] OR cholangio*[tiab] OR chondrob*[tiab] OR chondrom*[tiab] OR chondros*[tiab] OR chord*[tiab] OR chorioa*[tiab] OR choriocarc*[tiab] OR chorioep*[tiab] OR chorionep*[tiab] OR chromaffinom*[tiab] OR collagenom*[tiab] OR comedocarcinom*[tiab] OR condylom*[tiab] OR "condylomata acuminata"[tiab] OR "condylomata acuminata"[mh] OR corticotrop*[tiab] OR craniopharyng*[tiab] OR cylindrom*[tiab] OR cystadeno*[tiab] OR cystoma*[tiab] OR cystosa*[tiab] OR dentinom*[tiab] OR dermatofibro*[tiab] OR "dermoid"[tiab] OR "desmoid"[tiab] OR desmoplastic*[tiab] OR "dictyota"[tiab] OR dysgerm*[tiab] OR dyskerat*[tiab] OR "dysmyelopoiesis"[tiab] OR dysplas*[tiab] OR ectomesenchym*[tiab] OR elastofibr*[tiab] OR enchondrom*[tiab] OR endotheliom*[tiab] OR ependymo*[tiab] OR epidermoid*[tiab] OR epitheliom*[tiab] OR erythrol*[tiab] OR erythropl*[tiab] OR esthesioneuro*[tiab] OR etiolog*[tiab] OR fibroaden*[tiab] OR fibrochond*[tiab] OR fibroe*[tiab] OR fibrofol*[tiab] OR fibroid*[tiab] OR fibrolip*[tiab] OR fibrom*[tiab] OR fibroodontom*[tiab] OR fibrosarcom*[tiab] OR fibrothecom*[tiab] OR fibroxantho*[tiab] OR ganglioblast*[tiab] OR gangliocytom*[tiab] OR gangliogliom*[tiab] OR ganglioneuro*[tiab] OR gastrinom*[tiab] OR "genes, neoplasm"[tiab] OR "genes, neoplasm"[mh] OR germinom*[tiab] OR glioblast*[tiab] OR gliom*[tiab] OR glomangio*[tiab] OR glucagonom*[tiab] OR gonadoblastom*[tiab] OR gonocytom*[tiab] OR gynandroblastom*[tiab] OR haemangio*[tiab] OR hamartom*[tiab] OR hemangio*[tiab] OR hepatoblastom*[tiab] OR hepatom*[tiab] OR hibernom*[tiab] OR hidradenom*[tiab] OR hidrocy*[tiab] OR hodgkin*[tiab] OR hydatidiform*[tiab] OR hydradenom*[tiab] OR hypernephrom*[tiab] OR "IARC"[tiab] OR immunocytom*[tiab] OR insulinom*[tiab] OR leiomyo*[tiab] OR lesion*[tiab] OR leukaemia*[tiab] OR leukemia*[tiab] OR leukoplak*[tiab] OR leukostas*[tiab] OR "leukostasis"[tiab] OR "leukostasis"[mh] OR lipoadenom*[tiab] OR lipoblastom*[tiab] OR lipom*[tiab] OR liposarcom*[tiab] OR luteinom*[tiab] OR luteom*[tiab] OR lymphangio*[tiab] OR lymphoepitheliom*[tiab] OR lymphom*[tiab] OR lymphoscintigraph*[tiab] OR macroglobulinem*[tiab] OR macroprolactinom*[tiab] OR malignan*[tiab] OR maltom*[tiab] OR masculinovoblastom*[tiab] OR mastocyto*[tiab] OR "mcf-7"[tiab] OR "medullo*"[tiab] OR "meigs syndrome"[tiab] OR melanoa*[tiab] OR melanocytom*[tiab] OR melanom*[tiab] OR meningio*[tiab] OR mesenchymom*[tiab] OR mesonephrom*[tiab] OR mesotheliom*[tiab] OR metaplas*[tiab] OR "metaplasia"[tiab] OR "metaplasia"[mh] OR metasta*[tiab] OR microgliom*[tiab] OR micrometastas*[tiab] OR "mucositis"[tiab] OR "mucositis"[mh] OR mycosis fungoides*[tiab] OR myelodysplas*[tiab] OR "myelodysplastic syndromes"[tiab] OR "myelodysplastic syndromes"[mh] OR "myelodysplastic-myeloproliferative diseases"[tiab] OR "myelodysplastic-myeloproliferative diseases"[mh] OR "myelofibrosis"[tiab] OR myelol*[tiab] OR myeloma*[tiab] OR myeloproliferat*[tiab] OR "myeloproliferative disorders"[tiab] OR "myeloproliferative disorders"[mh] OR myelosuppression*[tiab] OR myoblastom*[tiab] OR myoepitheliom*[tiab] OR myofibro*[tiab] OR myolipom*[tiab] OR myoma*[tiab] OR myosarcom*[tiab] OR myxof*[tiab] OR myxom*[tiab] OR "naevus"[tiab] OR neoplas*[tiab] OR "neoplasm proteins"[tiab] OR "neoplasm proteins"[mh] OR "neoplasms"[tiab] OR "neoplasms"[mh] OR "neoplastic stem cells"[tiab] OR "neoplastic stem cells"[mh] OR nephroblastom*[tiab] OR neurilem*[tiab] OR neurinom*[tiab] OR neuroblastom*[tiab] OR neurocytom*[tiab] OR neuroepitheliom*[tiab] OR neurofibro*[tiab] OR neurolipocytom*[tiab] OR neuroma*[tiab] OR neuronevus[tiab] OR neurothekeom*[tiab] OR "nevus"[tiab] OR "non coding RNA"[tiab] OR nonseminom*[tiab] OR odontoam*[tiab] OR odontom*[tiab] OR oligoastrocytom*[tiab] OR oligodendrogliom*[tiab] OR oncocytom*[tiab] OR "oncogen*"[tiab] OR "oncogene fusion"[tiab] OR "oncogene fusion"[mh] OR "oncogene proteins"[tiab] OR "oncogene proteins"[mh] OR "oncogenic viruses"[tiab] OR "oncogenic viruses"[mh] OR oncolog*[tiab] OR "oncolytic viruses"[tiab] OR "oncolytic viruses"[mh] OR oncoprotein*[tiab] OR "opsoclonus-myoclonus"[tiab] OR orchioblastom*[tiab] OR osteoblastom*[tiab] OR osteoch*[tiab] OR osteofibrosarcom*[tiab] OR osteom*[tiab] OR osteosarcom*[tiab] OR pancreatoblastom*[tiab] OR papillom*[tiab] OR parachordom*[tiab] OR paragangliom*[tiab] OR paraneoplas*[tiab] OR perineuriom*[tiab] OR phaeochromocytom*[tiab] OR pheochromo*[tiab] OR pilomatri*[tiab] OR plasmacytom*[tiab] OR pneumoblast*[tiab] OR pneumocytom*[tiab] OR polyembryom*[tiab] OR polyhistiom*[tiab] OR polyp*[tiab] OR "polyps"[tiab] OR "polyps"[mh] OR porocarcinom*[tiab] OR porom*[tiab] OR pre-cancer*[tiab] OR precancer*[tiab] OR preleukaem*[tiab] OR preleukem*[tiab] OR prelymphom*[tiab] OR pre-lymphom*[tiab] OR pre-malign*[tiab] OR premalignan*[tiab] OR preneoplas*[tiab] OR pre-neoplas*[tiab] OR prolactinom*[tiab] OR protooncogen*[tiab] OR pseudotum*[tiab] OR reninom*[tiab] OR retinoblastom*[tiab] OR rhabdo*[tiab] OR "RNA, neoplasm"[tiab] OR "RNA, neoplasm"[mh] OR sarcoma*[tiab] OR schwannom*[tiab] OR "SEER program"[tiab] OR "SEER program"[mh] OR seminom*[tiab] OR "sentinel lymph node"[tiab] OR "sentinel lymph node biopsy"[tiab] OR "sentinel lymph node biopsy"[mh] OR "sertoli-leydig cell tumor"[tiab] OR "sezary syndrome"[tiab] OR somatostatinom*[tiab] OR somatotropinom*[tiab] OR spermatocytom*[tiab] OR spiradenom*[tiab] OR spongioblastom*[tiab] OR subependymom*[tiab] OR thecom*[tiab] OR thymom*[tiab] OR trichilemmom*[tiab] OR trichoadenom*[tiab] OR trichoblastom*[tiab] OR trichodiscom*[tiab] OR trichoepitheliom*[tiab] OR trichofolliculom*[tiab] OR tricholemm*[tiab] OR "tumor"[tiab] OR "tumor markers, biological"[tiab] OR "tumor markers, biological"[mh] OR tumorgen*[tiab] OR tumorig*[tiab] OR tumor-inhibit*[tiab] OR tumorog*[tiab] OR "tumors"[tiab] OR "tumors"[tiab] OR "tumour"[tiab] OR up-regulat*[tiab] OR vipom*[tiab] OR waldenstrom*[tiab] OR xantho*[tiab])

Neurological

(acetylcholine*[tiab] OR "ADHD"[tiab] OR adrenergic*[tiab] OR "adrenoleukodystrophy"[tiab] OR afferent*[tiab] OR "agoraphobia"[tiab] OR alzheimer*[tiab] OR amacrine*[tiab] OR "amnesia"[tiab] OR "amygdala"[tiab] OR "angelman-syndrome"[tiab] OR "anorexia"[tiab] OR antisocial*[tiab] OR anxiet*[tiab] OR anxious*[tiab] OR aphasi*[tiab] OR "aphonia"[tiab] OR apraxia*[tiab] OR "arachnoid"[tiab] OR "arousal"[tiab] OR astrocyte*[tiab] OR ataxia*[tiab] OR attention-deficit*[tiab] OR autis*[tiab] OR autonomic*[tiab] OR axon*[tiab] OR "baroreflex"[tiab] OR binge-eat*[tiab] OR "bipolar"[tiab] OR bovine-spongiform*[tiab] OR "brain"[tiab] OR "bulimia"[tiab] OR canavan*[tiab] OR cannabinoid*[tiab] OR "capgras"[tiab] OR cerebellar*[tiab] OR cerebral*[tiab] OR cerebro*[tiab] OR "cervical-cord"[tiab] OR charcot-marie-tooth*[tiab] OR "child behavior"[tiab] OR chronic-fatigue*[tiab] OR "circumventricular"[tiab] OR "cockayne-syndrome"[tiab] OR "cognition"[tiab] OR "cognitiv*"[tiab] OR "corpus callosum"[tiab] OR "cortical"[tiab] OR cranial*[tiab] OR "craniocerebral"[tiab] OR creutzfeldt-jakob*[tiab] OR "cyclothymi*"[tiab] OR "delirium"[tiab] OR "dementia"[tiab] OR demyelinat*[tiab] OR dendrit*[tiab] OR "dentate-gyrus"[tiab] OR "depressed"[tiab] OR "depression"[tiab] OR developmental-disabilit*[tiab] OR "dissociative"[tiab] OR dopamine*[tiab] OR "down-syndrome"[tiab] OR "drug-abuse"[tiab] OR "dura-matter"[tiab] OR dysautonomia*[tiab] OR dyscalcul*[tiab] OR dyskines*[tiab] OR dyslexi*[tiab] OR "dysphonia"[tiab] OR dyssomnia*[tiab] OR dyston*[tiab] OR eating-disorder*[tiab] OR efferent*[tiab] OR "encephalitis"[tiab] OR encephalo*[tiab] OR "entorhinal cortex"[tiab] OR ependy*[tiab] OR "epilep*"[tiab] OR "epithalamus"[tiab] OR "essential-tremor"[tiab] OR excitatory amino acid*[tiab] OR "extra-pyramidal"[tiab] OR extrapyramidal*[tiab] OR "fibromyalgia"[tiab] OR "friedreich ataxia"[tiab] OR "fronto-temporal"[tiab] OR frontotemporal*[tiab] OR ganglia*[tiab] OR ganglion*[tiab] OR "glia"[tiab] OR "glial"[tiab] OR "gliogenesis"[tiab] OR glossopharyngeal*[tiab] OR "gray-matter"[tiab] OR guillain-barre*[tiab] OR "hemiplegia"[tiab] OR hippocamp*[tiab] OR huntington*[tiab] OR "hydranencephaly"[tiab] OR hydrocephal*[tiab] OR "hyperkinesis"[tiab] OR hypochondr*[tiab] OR "hypokinesia"[tiab] OR hypomani*[tiab] OR "hypotha*"[tiab] OR insomnia*[tiab] OR "intell*"[tiab] OR "interneuron"[tiab] OR "inter-neuron"[tiab] OR intracranial*[tiab] OR "IQ"[tiab] OR "ischemi*"[tiab] OR "learning"[tiab] OR leukodystrophy*[tiab] OR leukoencephal*[tiab] OR lewy-bod*[tiab] OR limbic*[tiab] OR "memory"[tiab] OR meningeal*[tiab] OR "meninges"[tiab] OR meningitis*[tiab] OR "meningoencephalitis"[tiab] OR "mesencephalon"[tiab] OR microglia*[tiab] OR mononeuropath*[tiab] OR "mood"[tiab] OR motor-skill*[tiab] OR movement-disorder*[tiab] OR multiple-personalit*[tiab] OR "Munchausen"[tiab] OR muscarinic*[tiab] OR muscular-dystroph*[tiab] OR "myalgia*"[tiab] OR myasthen*[tiab] OR "myeli*"[tiab] OR "myoclonus"[tiab] OR "myokymia"[tiab] OR myopath*[tiab] OR "myositis"[tiab] OR myotoni*[tiab] OR nerve*[tiab] OR "nervous system"[tiab] OR "nervous system"[mh] OR "nervous system diseases"[tiab] OR "nervous system diseases"[mh] OR "nervous system physiological phenomema"[tiab] OR "nervous system physiological phenomema"[mh] OR nervous*[tiab] OR neural*[tiab] OR "neurit*"[tiab] OR "neuroaspergillosis"[tiab] OR neuroaxon*[tiab] OR neuro-axon*[tiab] OR neurobehav*[tiab] OR neurodegenerat*[tiab] OR "neuroeffector"[tiab] OR neuroendocrine*[tiab] OR neurofib*[tiab] OR neurofun*[tiab] OR neurogen*[tiab] OR "neuroglia*"[tiab] OR "neuroim*"[tiab] OR neurokinin*[tiab] OR neurologic*[tiab] OR neuromuscular*[tiab] OR neuromyelitis*[tiab] OR neuron*[tiab] OR neuropath*[tiab] OR "neuropil"[tiab] OR neurosecret*[tiab] OR neurotox*[tiab] OR neurotrans*[tiab] OR "neurotransmitter agents"[tiab] OR "neurotransmitter agents"[mh] OR nicotinic*[tiab] OR nissl-bod*[tiab] OR obsessive-compulsive*[tiab] OR "OCD"[tiab] OR oculomotor*[tiab] OR "olfact*"[tiab] OR "oligodendroglia"[tiab] OR ophthalmoplegia*[tiab] OR palsy*[tiab] OR "panic"[tiab] OR parahippocamp*[tiab] OR "paraly*"[tiab] OR parano*[tiab] OR "paraparesis"[tiab] OR "paraplegia"[tiab] OR parasomnia*[tiab] OR "paresis"[tiab] OR parkinson*[tiab] OR perforant*[tiab] OR perimeningeal*[tiab] OR "personality"[tiab] OR phob*[tiab] OR "pica"[tiab] OR piloerect*[tiab] OR pineal*[tiab] OR pituitary*[tiab] OR plasticity*[tiab] OR "poliomyelitis"[tiab] OR polyneuropath*[tiab] OR polyradicul*[tiab] OR "potentia*"[tiab] OR prader-willi*[tiab] OR "premenstrual dysphoric disorder"[tiab] OR presynap*[tiab] OR "primary dysautonomias"[tiab] OR prion*[tiab] OR propriocept*[tiab] OR "prosencephalon"[tiab] OR "psychiatry and psychology category"[tiab] OR "psychiatry and psychology category"[mh] OR psychomotor*[tiab] OR purinergic*[tiab] OR "radicul*"[tiab] OR receptor*[tiab] OR "receptors, cell surface"[tiab] OR "receptors, cell surface"[mh] OR reflex*[tiab] OR "rett-syndrome"[tiab] OR "rhabdomyolysis"[tiab] OR "rhombencephalon"[tiab] OR rhythm*[tiab] OR schizophreni*[tiab] OR schwann-cell*[tiab] OR sclerosis*[tiab] OR scrapi*[tiab] OR "season* affective disorder"[tiab] OR seizure*[tiab] OR senil*[tiab] OR sensation*[tiab] OR "sensory gating"[tiab] OR seroton*[tiab] OR sleep*[tiab] OR somatosensory*[tiab] OR speech*[tiab] OR spinal-cord*[tiab] OR spinocerebellar*[tiab] OR "stress"[tiab] OR "stroke"[tiab] OR subarachnoid*[tiab] OR subdural*[tiab] OR "substance abuse"[tiab] OR "substantia-nigra"[tiab] OR synap*[tiab] OR "syncope"[tiab] OR tauopath*[tiab] OR "thalamic"[tiab] OR tic-disorder*[tiab] OR tourette*[tiab] OR "vagal"[tiab] OR vagus*[tiab] OR "vertigo"[tiab] OR "voice disorders"[tiab] OR "white-matter"[tiab] OR "williams-syndrome"[tiab] OR "wolfram-syndrome"[tiab])

Liver/Hepatic

((portal[tiab] AND hypertension[tiab]) OR ("alanine aminotransferase"[tiab] OR "alanine aminotransferase"[mh] OR "alkaline phosphatase"[tiab] OR "alkaline phosphatase"[mh] OR aspartate aminotransferase*[tiab] OR "aspartate aminotransferases"[tiab] OR "aspartate aminotransferases"[mh] OR bilirubin*[tiab] OR bilirubin*[mh] OR cholestasis*[tiab] OR cirrhosis*[tiab] OR "erythropoietic protoporphyria"[tiab] OR "extrahepatic"[tiab] OR "fascioliasis"[tiab] OR "focal nodular hyperplasia"[tiab] OR hepatic*[tiab] OR hepatitis*[tiab] OR "hepato"[tiab] OR hepatobil*[tiab] OR "hepatoc*"[tiab] OR "hepatocytes"[tiab] OR "hepatocytes"[mh] OR hepatolent*[tiab] OR hepatomeg*[tiab] OR hepatopulm*[tiab] OR hepato-pulm*[tiab] OR hepatorenal*[tiab] OR hepato-renal*[tiab] OR hepatotox*[tiab] OR hepato-tox*[tiab] OR hyperbilirubin*[tiab] OR "hyperbilirubinemia"[tiab] OR "hyperbilirubinemia"[mh] OR intrahepatic*[tiab] OR intra-hepatic*[tiab] OR jaundice*[tiab] OR "liver"[tiab] OR "liver"[mh] OR "liver diseases"[tiab] OR "liver diseases"[mh] OR "liver function tests"[tiab] OR "liver function tests"[mh] OR liver*[tiab] OR porphyria*[tiab] OR "Reye syndrome"[tiab] OR "Reye syndrome"[mh]))

Immunological

(diabetes[tiab] AND type 1[tiab]) OR (hepatitis[tiab] AND autoimmune[tiab]) OR (addison*[tiab] OR adhesin*[tiab] OR agglutinat*[tiab] OR allergen*[tiab] OR allergi*[tiab] OR alpha-fetoprotein*[tiab] OR anaphylatoxin*[tiab] OR anemi*[tiab] OR angiotensin*[tiab] OR antibod*[tiab] OR anticoagulan*[tiab] OR "antifibrinolytic agents"[tiab] OR antigen*[tiab] OR "antigens"[tiab] OR "antigens"[mh] OR antisickling agent*[tiab] OR antithrombin*[tiab] OR "arrestin"[tiab] OR arthritis*[tiab] OR autoantigen*[tiab] OR autocoid*[tiab] OR autoimmun*[tiab] OR basophil*[tiab] OR b-cell*[tiab] OR bleed*[tiab] OR "blood physiological phenomena"[tiab] OR "blood physiological phenomena"[mh] OR "blood proteins"[tiab] OR "blood proteins"[mh] OR blood*[tiab] OR b-lymphocyt*[tiab] OR "bone-marrow"[tiab] OR "cd25"[tiab] OR "cd27"[tiab] OR "cd28"[tiab] OR "cd29"[tiab] OR "cd3"[tiab] OR "cd4"[tiab] OR "cd45"[tiab] OR "cd8"[tiab] OR chemokine*[tiab] OR "churg-strauss syndrome"[tiab] OR coagulat*[tiab] OR "coccidioidin"[tiab] OR "crp"[tiab] OR cytokine*[tiab] OR cytophagocytos*[tiab] OR dendrit*[tiab] OR dermatitis*[tiab] OR eicosanoid*[tiab] OR enterochromaffin*[tiab] OR eosinophil*[tiab] OR epitheloid-cell*[tiab] OR "epitope mapping"[tiab] OR erythrocyte*[tiab] OR fibrin-clot*[tiab] OR fibrinoly*[tiab] OR fluoroimmunoas*[tiab] OR foam-cell*[tiab] OR gamma-globulin*[tiab] OR giant cell*[tiab] OR glomerulonephritis*[tiab] OR granulocyte*[tiab] OR "graves disease"[tiab] OR guillain-barre*[tiab] OR "haematopoietic"[tiab] OR "haemic"[tiab] OR hemangioma*[tiab] OR hematinic*[tiab] OR hematocrit*[tiab] OR "hematologic agents"[tiab] OR "hematologic agents"[mh] OR "hematologic diseases"[tiab] OR "hematologic diseases"[mh] OR "hematologic tests"[tiab] OR "hematologic tests"[mh] OR hematologic*[tiab] OR hematop*[tiab] OR "hemic and immune systems"[tiab] OR "hemic and immune systems"[mh] OR hemocyte*[tiab] OR hemoglo*[tiab] OR "hemolytic"[tiab] OR hemophil*[tiab] OR hemorheolog*[tiab] OR hemorrhag*[tiab] OR hemostas*[tiab] OR hemostatic*[tiab] OR histamine*[tiab] OR histocompatib*[tiab] OR "histoplasmin"[tiab] OR "host-resistance"[tiab] OR hyperresponsiv*[tiab] OR hypersensitiv*[tiab] OR "il-6"[tiab] OR "il-8"[tiab] OR "immune system diseases"[tiab] OR "immune system diseases"[mh] OR "immune system phenomena"[tiab] OR "immune system phenomena"[mh] OR immune*[tiab] OR immunit*[tiab] OR immunoassay*[tiab] OR immunobl*[tiab] OR immunochroma*[tiab] OR immunoco*[tiab] OR immunog*[tiab] OR immunolog*[tiab] OR "immunologic techniques"[tiab] OR "immunologic techniques"[mh] OR "immunologic tests"[tiab] OR "immunologic tests"[mh] OR immunom*[tiab] OR immunophenotyp*[tiab] OR immunopr*[tiab] OR immunosuppress*[tiab] OR immunotherap*[tiab] OR immunotox*[tiab] OR inflamm*[tiab] OR "inflammation"[tiab] OR "inflammation"[mh] OR "inflammation mediators"[tiab] OR "inflammation mediators"[mh] OR "insulin-dependent"[tiab] OR interferon*[tiab] OR interleukin*[tiab] OR isoimmunizat*[tiab] OR killer cell*[tiab] OR kinin*[tiab] OR kupffer-cell*[tiab] OR langerhans*[tiab] OR "lepromin"[tiab] OR leukocyte*[tiab] OR leukopoies*[tiab] OR lupus*[tiab] OR lymphoc*[tiab] OR lymphokine*[tiab] OR lymphom*[tiab] OR lymphop*[tiab] OR macrophage*[tiab] OR mast-cell*[tiab] OR monocyte*[tiab] OR monokine*[tiab] OR "multiple sclerosis"[tiab] OR "myasthenia gravis"[tiab] OR myelop*[tiab] OR "neprilysin"[tiab] OR neutrophil*[tiab] OR nk-cell*[tiab] OR "osmotic fragility"[tiab] OR phagocyt*[tiab] OR "plasma"[tiab] OR platelet*[tiab] OR polyradiculoneuropath*[tiab] OR prostaglandin*[tiab] OR protein-c-deficienc*[tiab] OR prothrombin*[tiab] OR purpura*[tiab] OR radioim*[tiab] OR reticulocyt*[tiab] OR rheumatoid*[tiab] OR sensitiz*[tiab] OR serodiagnosis*[tiab] OR serotyp*[tiab] OR sperm agglutinat*[tiab] OR "spleen"[tiab] OR "splenic"[tiab] OR splenocyte*[tiab] OR staphylococc*[tiab] OR t-cell*[tiab] OR "t-helper"[tiab] OR thrombin*[tiab] OR thromboc*[tiab] OR thrombop*[tiab] OR "thymic"[tiab] OR thymocyte*[tiab] OR thymus*[tiab] OR t-lympho*[tiab] OR "tnf alpha"[tiab] OR "transverse myelitis"[tiab] OR "trichophytin"[tiab] OR vaccinat*[tiab] OR vaccine*[tiab] OR von-willebrand*[tiab])

Evidence Stream Strings

Note that population strings are based on SWIFT-Review search filters [[1](#_ENREF_1)].

Human and Epidemiological Terms

(humans[mh] OR "human development"[mh]) OR (human[tiab] OR Humans[tiab] OR person[tiab] OR people[tiab]) OR ((“age groups”[mh]) OR (pediatric[tiab] OR pediatrician[tiab] OR paediatric[tiab] OR paediatrician[tiab] OR baby [tiab]OR babies[tiab] OR toddler[tiab] OR toddlers[tiab] OR child[tiab] OR children[tiab] OR youth[tiab] OR youngster[tiab] OR tween[tiab] OR tweens[tiab] OR teen[tiab] OR teens[tiab] OR teenager[tiab] OR teenagers[tiab] OR teenaged[tiab]) OR (("in utero"[tiab] OR prenatal[tiab] OR perinatal[tiab] OR neonatal[tiab] OR postnatal[tiab]) NOT (mice[tiab] OR mouse[tiab] OR rat[tiab] OR rats[tiab]))) OR (preschool[tiab] OR preschooler[tiab] OR pre-school[tiab] OR kindergarten[tiab] OR kindergartener[tiab] OR schoolchild[tiab] OR schoolchildren[tiab] OR student[tiab] OR students[tiab]) OR ("middle age"[tiab] OR “middle-aged”[tiab] OR aged[tiab] OR elder[tiab] OR elderly[tiab] OR "senior citizen"[tiab] OR seniors[tiab] OR retiree[tiab] OR septuagenarian[tiab] OR octagenarian[tiab] OR sexagenarian[tiab] OR nonagenarian[tiab] OR centenarian[tiab]) OR ("nuclear family"[mh]) OR (family[tiab] OR families[tiab] OR parent[tiab] OR parents[tiab] OR father[tiab] OR fathers[tiab] OR mother[tiab] OR mothers[tiab] OR sibling[tiab] OR siblings[tiab] OR brother[tiab] OR brothers[tiab] OR sister[tiab] OR sisters[tiab] OR twin[tiab] OR twins[tiab] OR "stepfather"[tiab] OR "step father"[tiab] OR "stepmother"[tiab] OR "step mother"[tiab] OR "stepdaughter"[tiab] OR "step daughter"[tiab] OR "stepson"[tiab] OR "step son"[tiab] OR aunt[tiab] OR aunts[tiab] OR uncle[tiab] OR uncles[tiab] OR niece[tiab] OR nieces[tiab] OR nephew[tiab] OR nephews[tiab] OR grandparent[tiab] OR grandparents[tiab] OR grandfather[tiab] OR "grand father"[tiab] OR grandmother[tiab] OR "grand mother"[tiab] OR grandchild[tiab] OR granddaughter[tiab] OR grandson[tiab] OR spouse[tiab] OR spouses[tiab] OR spousal[tiab] OR partner[tiab] OR partners[tiab] OR husband[tiab] OR husbands[tiab] OR wife[tiab] OR wives[tiab] OR guardian[tiab] OR caregiver[tiab] OR caregivers[tiab] OR "care giver"[tiab]) OR (men[mh] OR women[mh]) OR (men[tiab] OR man[tiab] OR boy[tiab] OR boys[tiab] OR boyhood[tiab] OR women[tiab] OR woman[tiab] OR girl[tiab] OR girls[tiab] OR girlhood[tiab]) OR ("population groups"[mh] OR "vulnerable populations"[mh]) OR ("african american"[tiab] OR "asian american"[tiab] OR hispanic[tiab] OR latina[tiab] OR latino[tiab] OR "mexican american"[tiab] OR underserved[tiab] OR disadvantaged[tiab]) OR ("epidemiologic studies"[mh] OR "double-blind method"[mh] OR "single-blind method"[mh]) OR (epidemiology[mh]) OR ("case control"[tiab] OR cohort[tiab] OR "cross sectional"[tiab] OR "follow-up study"[tiab] OR longitudinal[tiab] OR prospective[tiab] OR retrospective[tiab]) OR ("case reports"[mh] OR "clinical trial"[mh] OR "observational study"[mh] OR "randomized control trial"[mh] OR "twin study"[mh]) OR ("clinical trial"[tiab] OR observational[tiab] OR "randomized control trial"[tiab]) OR ("research subjects"[mh] OR "human experimentation"[mh] OR patients[mh] OR "Patient Participation"[mh]) OR ("human subjects"[tiab] OR "research subjects"[tiab] OR clients[tiab] OR patient[tiab] OR inpatient[tiab] OR outpatient[tiab] OR participants[tiab] OR volunteers[tiab]) OR ("occupational groups"[mh] OR "occupational exposure"[mh]) OR (occupation[tiab] OR occupational[tiab] OR workplace[tiab] OR "work place"[tiab] OR "work-related"[tiab] OR administrators[tiab] OR aides[tiab] OR assistants[tiab] OR crew[tiab] OR crews[tiab] OR employees[tiab] OR personnel[tiab] OR professional[tiab] OR staff[tiab] OR technicians[tiab] OR workers[tiab] OR educators[tiab] OR instructors[tiab] OR teachers[tiab] OR clinicians[tiab] OR doctors[tiab] OR physicians[tiab] OR pharmacists[tiab] OR nurses[tiab] OR residents[tiab] OR veterinarians[tiab]) OR (epidemiologic[tiab])

Animal

"animal experimentation”[mh] OR "models, animal”[mh] OR "behavior, animal”[mh] OR "animal population groups”[mh] OR "invertebrates”[mh] OR "chordata, nonvertebrate”[mh] OR "amphibians”[mh] OR "birds”[mh] OR "fishes”[mh] OR "reptiles”[mh] OR "carnivora”[mh] OR "insectivora”[mh] OR "lagomorpha”[mh] OR "rodentia”[mh] OR "strepsirhini”[mh] OR "platyrrhini"[mh] OR "tarsii”[mh] OR "cercopithecidae”[mh] OR "hylobatidae”[mh] OR "gorilla gorilla”[mh] OR "pan paniscus”[mh] OR "pan troglodytes”[mh] OR "pongo pygmaeus”[mh] OR "Animals”[mh] OR "chordata”[mh] OR "vertebrates”[mh] OR "mammals”[mh] OR "primates”[mh] OR "haplorhini”[mh] OR "catarrhini”[mh] OR "hominidae”[mh] OR animal[tiab] OR animals[tiab] OR mice[tiab] OR mus[tiab] OR mouse[tiab] OR murine[tiab] OR rats[tiab] OR rat[tiab] OR murinae[tiab] OR muridae[tiab] OR "cotton rat"[tiab] OR "cotton rats"[tiab] OR hamster[tiab] OR hamsters[tiab] OR rodent[tiab] OR rodents[tiab] OR pigs[tiab] OR pig[tiab] OR swine[tiab] OR piglet[tiab] OR piglets[tiab] OR "guinea pigs"[tiab] OR "guinea pig"[tiab] OR cavia[tiab] OR callithrix[tiab] OR marmoset[tiab] OR marmosets[tiab] OR cebuella[tiab] OR hapale[tiab] OR octodon[tiab] OR chinchilla[tiab] OR chincillas[tiab] OR gerbillinae[tiab] OR gerbil[tiab] OR gerbils[tiab] OR rabbit[tiab] OR rabbits[tiab] OR hares[tiab] OR hare[tiab] OR cats[tiab] OR cat[tiab] OR carus[tiab] OR felis[tiab] OR dogs[tiab] OR dog[tiab] OR canine[tiab] OR canines[tiab] OR canis[tiab] OR haplorhini[tiab] OR monkey[tiab] OR monkeys[tiab] OR anthropoid[tiab] OR saguinus[tiab] OR tamarin[tiab] OR leontopithecus[tiab] OR hominidae[tiab] OR ape[tiab] OR apes[tiab] OR "pan paniscus"[tiab] OR bonobo[tiab] OR "pan troglodytes"[tiab] OR gibbon[tiab] OR gibbons[tiab] OR nomascus[tiab] OR symphalangus[tiab] OR chimpanzee[tiab] OR chimpanzees[tiab] OR chimp[tiab] OR chimps[tiab] OR prosimian[tiab] OR pongidae[tiab] OR gorilla[tiab] OR gorillas[tiab] OR "pongo pygmaeus"[tiab] OR orangutan[tiab] OR orangutans[tiab] OR lemur[tiab] OR lemurs[tiab] OR lemuridae[tiab] OR chicken[tiab] OR chickens[tiab] OR gallus[tiab] OR quail[tiab] OR quails[tiab] OR bird[tiab] OR birds[tiab] OR poultry[tiab] OR fowl[tiab] OR fowls[tiab] OR reptile[tiab] OR reptiles[tiab] OR turtle[tiab] OR turtles[tiab] OR amphibian[tiab] OR frog[tiab] OR frogs[tiab] OR xenopus[tiab] OR bombina[tiab] OR salientia[tiab] OR toad[tiab] OR toads[tiab] OR "epidalea calamita"[tiab] OR salamander[tiab] OR fish[tiab] OR fishes[tiab] OR pisces[tiab] OR catfish[tiab] OR perch[tiab] OR percidae[tiab] OR perca[tiab] OR trout[tiab] OR char[tiab] OR salmon[tiab] OR salvelinus[tiab] OR minnow[tiab] OR cyprinidae[tiab] OR carp[tiab] OR zebrafish[tiab] OR "zebra fish"[tiab] OR nematode[tiab] OR elegans[tiab] OR diptera[tiab] OR flies[tiab] OR dipteral[tiab] OR drosophila[tiab]

Study Type

Reviews

("Meta-Analysis"[pt] OR "Review"[pt] OR "Systematic Review" [pt] OR review[ti] OR metaanalysis[tiab] OR case-report[tiab] OR metaanalyses[tiab] OR meta-analysis[tiab] OR meta-analyses[tiab

Supplemental Tables

Table S1 Production Volumes of East Palestine Chemicals of Interest from EPA’s 2020 Chemical Data Reporting [[2](#_ENREF_2)]

| Chemical Name  CASRN | 2018 Nationally Aggregated Production Volume (lbs/yr) | 2019 Nationally Aggregated Production Volume (lbs/yr) |
| --- | --- | --- |
| Acrolein  107-02-8 | 250,000,000 – <500,000,000 | 250,000,000 – <500,000,000 |
| Benzene  71-43-2 | 10,000,000,000 – <20,000,000,000 | 10,000,000,000 – <20,000,000,000 |
| Butyl acrylate  141-32-2 | 1,000,000,000 – <5,000,000,000 | 1,000,000,000 – <5,000,000,000 |
| Diethylene glycol  111-46-6 | 750,000,000 – <1,000,000,000 | 750,000,000 – <1,000,000,000 |
| Dipropylene glycol  25265-71-8 | 100,000,000 – <250,000,000 | 100,000,000 – <250,000,000 |
| Ethylene glycol monobutyl ether (2-Butoxyethanol)  111-76-2 | 500,000,000 – <750,000,000 | 500,000,000 – <750,000,000 |
| 2-Ethylhexyl acrylate  103-11-7 | 250,000,000 – <500,000,000 | 250,000,000 – <500,000,000 |
| Hydrogen chloride  7647-01-0 | 10,000,000,000 – <20,000,000,000 | 10,000,000,000 – <20,000,000,000 |
| Isobutylene  115-11-7 | 1,000,000,000 – <5,000,000,000 | 1,000,000,000 – <5,000,000,000 |
| Petroleum lube oil  64742-58-1 | 500,000,000 – <750,000,000 | 1,000,000,000 – <5,000,000,000 |
| Phosgene gas  75-44-5 | 250,000,000 – <500,000,000 | 250,000,000 – <500,000,000 |
| Polyethylene  9002-88-4 | 1,874,993 | 100,536 |
| Polypropylene glycol  25322-69-4 | <1,000,000 | <1,000,000 |
| 1,2-Propylene glycol  57-55-6 | 1,000,000,000 – <5,000,000,000 | 1,000,000,000 – <5,000,000,000 |
| Polyvinyl alcohol  9002-89-5 | 60,146 | 29,720 |
| Vinyl chloride  75-01-4 | 10,000,000,000 – <20,000,000,000 | 10,000,000,000 – <20,000,000,000 |
| 6:2 FTSHA  88992-45-4 | <1,000,000 | <1,000,000 |
| 6:2 FTSAS  88992-47-6 | Not found | Not found |
| 6:2 FTSA  27619-97-2 | 25,000 – <100,000 | 25,000 – <100,000 |
| 6:2 FTSA-PrB  34455-29-3 | <1,000,000 | <1,000,000 |
| 6:2 FTNO  80475-32-7 | Not found | Not found |
| 2,3,7,8-TCDD  1746-01-6 | Not found | Not found |

6:2 FTNO = 6:2 fluorotelomer sulfonamido amine oxide; 6:2 FTSHA = 6:2 fluorotelomer thiohydroxyammonium; 6:2 FTSAS = 6:2 fluorotelomermercap-toalkylamido sulfonate; 6:2 FTSA = 6:2 fluorotelomer sulfonic acid; 6:2 FTSA-PrB = 6:2 fluorotelomer sulfonamide alkylbetaine; TCDD = 2,3,7,8-tetrachlorodibenzo-p-dioxin.

Table S2 Population, Exposure, Comparator, and Outcome/Evidence Stream, Exposure, Comparator, and Outcome Statements

| Publication Type/Evidence Stream | Population or Evidence Type (Primary Studies) | Exposure^a^ | Comparison Group^b^ | Outcome^c^ |
| --- | --- | --- | --- | --- |
| Human epidemiological reviews:^d^ Post recent IARC publication [[3](#_ENREF_3)]  Primary epidemiological studies: Post recent IARC publication [[3](#_ENREF_3)]; not restricted to design   - Studies reporting risk estimate or correlation (ecological studies) - Case reports, case series | Humans: Workers, community, not restricted | Acrolein | Low or no exposure to acrolein | Cancer |
| Human epidemiological, animal, mechanistic reviews | NA | Acrolein | Low or no exposure to acrolein | Neurotoxicity |
| Human epidemiological reviews^d^  Primary epidemiological studies   - Studies reporting risk estimate or correlation (ecological studies) - Case reports, case series | Humans: Workers, community, not restricted | 2-Butoxyethanol | Low or no exposure to 2-butoxyethanol | Cancer |
| Human epidemiological, animal, mechanistic reviews  Primary epidemiological and toxicology studies   - Studies reporting risk estimate or correlation (ecological studies) - Case reports, case series | Humans: Workers, community, not restricted  Animals: Nonhuman mammalian | 2-Butoxyethanol | Low or no exposure to 2-butoxyethanol | Immunotoxicity |
| Human epidemiological, animal, mechanistic reviews  Primary epidemiological and toxicology studies   - Studies reporting risk estimate or correlation (ecological studies) - Case reports, case series | Humans: Workers, community, not restricted  Animals: Nonhuman mammalian | 2-Butoxyethanol | Low or no exposure to 2-butoxyethanol | Neurotoxicity |
| Human epidemiological, animal, mechanistic reviews  Primary epidemiological and toxicology studies   - Studies reporting risk estimate or correlation (ecological studies) - Case reports, case series | Humans: Workers, community, not restricted  Animals: Nonhuman mammalian | Butyl acrylate | Low or no exposure to butyl acrylate | Cancer |
| Human epidemiological, animal, mechanistic reviews  Primary epidemiological and toxicology studies   - Studies reporting risk estimate or correlation (ecological studies) - Case reports, case series | Humans: Workers, community, not restricted  Animals: Nonhuman mammalian | Butyl acrylate | Low or no exposure to butyl acrylate | Hepatotoxicity |
| Human epidemiological, animal, mechanistic reviews  Primary epidemiological and toxicology studies   - Studies reporting risk estimate or correlation (ecological studies) - Case reports, case series | Humans: Workers, community, not restricted  Animals: Nonhuman mammalian | Butyl acrylate | Low or no exposure to butyl acrylate | Immunotoxicity |
| Human epidemiological, animal, mechanistic reviews  Primary epidemiological and toxicology studies   - Studies reporting risk estimate or correlation (ecological studies) - Case reports, case series | Humans: Workers, community, not restricted  Animals: Nonhuman mammalian | Butyl acrylate | Low or no exposure to butyl acrylate | Neurotoxicity |
| Human epidemiological reviews:^d^ Post recent IARC publication [[4](#_ENREF_4)]  Primary epidemiological studies: Post recent IARC publication [[4](#_ENREF_4)]; not restricted to design   - Studies reporting risk estimate or correlation (ecological studies) - Case reports, case series | Humans: Workers, community, not restricted | 2-Ethylhexyl acrylate | Low or no exposure to 2-ethylhexyl acrylate | Cancer |
| Human epidemiological, animal, mechanistic reviews  Primary epidemiological and toxicology studies   - Studies reporting risk estimate or correlation (ecological studies) - Case reports, case series | Humans: Workers, community, not restricted  Animals: Nonhuman mammalian | 2-Ethylhexyl acrylate | Low or no exposure to 2-ethylhexyl acrylate | Neurotoxicity |
| Human epidemiological, animal, mechanistic reviews  Primary epidemiological and toxicology studies   - Studies reporting risk estimate or correlation (ecological studies)   Case reports, case series | Humans: Workers, community, not restricted  Animals: Nonhuman mammalian | 6:2 FTSA | Low or no exposure to 6:2 FTSA | Any health outcome |
| Human epidemiological, animal, mechanistic reviews  Primary epidemiological and toxicology studies   - Studies reporting risk estimate or correlation (ecological studies)   Case reports, case series | Humans: Workers, community, not restricted  Animals: Nonhuman mammalian | 6:2 FTNO | Low or no exposure to 6:2 FTNO | Any health outcome |
| Human epidemiological, animal, mechanistic reviews | NA | Dioxins other than TCDD | Low or no exposure to dioxins other than TCDD | Any health outcome |

IARC = International Agency for Research on Cancer; NA = not applicable; TCDD = 2,3,7,8-tetrachlorodibenzo-p-dioxin.

^a^Includes all routes, all life stages, and exposure proxies (e.g., biomarkers); does not include references studying exposure from only endogenous formation.

^b^Case reports/case series do not include a nonexposed control group but were considered PECO relevant for this review.

^c^Outcomes are defined in Table S3.

^d^Reviews reporting on individual epidemiological studies.

Table S3 Health Outcome Concepts

| Outcomes | Example Categories | |
| --- | --- | --- |
| Cancer: Organ Systems | - Digestive/gastrointestinal - Endocrine - Female reproductive - Head and neck - Hematologic/lymphatic/immune - Hepatic - Male reproductive - Musculoskeletal | - Nervous - Respiratory - Skin - Special senses - Systemic - Urinary - Other |
| Hepatotoxicity | - Albumin - Albumin/globulin ratio - Bile acids/salts - Bilirubin - Hepatic steatosis/fatty liver | - Liver disease - Liver enzymes (e.g., alanine transaminase, aspartate transferase, alkaline phosphatase) - Liver-specific serum biochemistry markers (e.g., gamma-glutamyl transferase, sorbitol dehydrogenase) - Other |
| Immunotoxicity | - Allergy - Autoimmune diseases (e.g., multiple sclerosis, lupus, rheumatoid arthritis) - General immune assays (e.g., white blood cell counts) - Hypersensitivity - Immunoglobulins (e.g., IgE, IgG, IgM) | - Infectious diseases - Serum globulin levels - Vaccine response - White blood cell activity assays - Other |
| Neurotoxicity | Human effect categories   - Academic achievement - Attention - Autonomic function - Clinical conditions (e.g., depression, Alzheimer’s disease, Parkinson’s disease, autism, intellectual disabilities) - Executive function - General intelligence (i.e., IQ) - Hearing impairment - Learning and memory - Motor function - Neurodevelopment - Peripheral nervous system - Social-emotional behavioral regulation - Verbal-language - Visuospatial function - Other | Animal effect categories   - Behavioral (e.g., sensory, motor, or learning and memory changes; may be measured by functional observation batteries) - Neurochemical (e.g., impacts to sodium or calcium levels/transmission, impacts to neurotransmitters and receptors, impacts to transport of important neurochemicals) - Neurophysiological (e.g., seizures; impacts to electrical activity, including never conduction and action or evoked potentials; tests of electrical activity) |

Table S4 Phase 1 Authoritative Sources with Available Data by Chemical

| Chemical  CASRN | [ATSDR](https://www.atsdr.cdc.gov/toxprofiledocs/index.html) [[5](#_ENREF_5)] | [ECHA](https://echa.europa.eu/information-on-chemicals) [[6](#_ENREF_6)] | [EPA CompTox](https://comptox.epa.gov/dashboard/) [[7](#_ENREF_7)] | [EPA IRIS](https://iris.epa.gov/AtoZ/?list_type=alpha) [[8](#_ENREF_8)] | [Health Canada](https://www.canada.ca/en/environment-climate-change/services/canadian-environmental-protection-act-registry/substances-list/priority-list.html) [[9](#_ENREF_9)] | [IARC](https://monographs.iarc.who.int/monographs-available/) [[10](#_ENREF_10)] | [NTP](https://ntp.niehs.nih.gov/data/tr) [[11](#_ENREF_11)] | [OEHHA](https://oehha.ca.gov/chemicals) [[12](#_ENREF_12)] | Additional Sources |
| --- | --- | --- | --- | --- | --- | --- | --- | --- | --- |
| Acrolein  107-02-8 | X | X | X | X | X | X | — | X | None |
| Benzene  71-43-2 | X | X | X | X | X | X | X^a,b^ | X | NIOSH Pocket Guide |
| Butyl acrylate  141-32-2 | — | X | X | — | X | X | — | — | OECD Screening Information Dataset Profile |
| Diethylene glycol  111-46-6 | — | X | X | — | X | — | X^c^ | — | OECD Screening Information Dataset Profile |
| Dipropylene glycol  25265-71-8 | — | X | X | — | — | — | X^b^ | — |  |
| Ethylene glycol monobutyl ether (2-butoxyethanol)  111-76-2 | X | X | X | X | X | X | X^b^ | X | NIOSH Pocket Guide |
| 2-Ethylhexyl acrylate  103-11-7 | — | X | X | — | X | X | — | X | None |
| Hydrogen chloride  7647-01-0 | X | X | X | X | X | X | — | X | NIOSH Pocket Guide; NRC AEGL Document |
| Isobutylene  115-11-7 | — | X | X | — | — | — | X^b^ | — | OECD Screening Information Dataset Profile |
| Petroleum lube oil  64742-58-1 | — | X | X | — | — | — | — | — | EPA Screening-Level Hazard Characterization Document |
| Phosgene gas  75-44-5 | — | X | X | X | — | — | — | X | NRC AEGL Document; INCHEM Health and Safety Guide; NIOSH Pocket Guide |
| Polyethylene  9002-88-4 | — | X | X | — | — | X | X^c^ | — | NIIOSH pocket guide |
| Polypropylene glycol  25322-69-4 | — | X | X | — | X | — | — | — | None |
| 1,2-Propylene glycol  57-55-6 | X | X | X | X | X | — | — | — | EPA PPRTV; NTP CERHR Monograph and studies; OSHA data |
| Polyvinyl alcohol  9002-89-5 | — | X | X | — | — | X | X^b^ | — |  |
| Vinyl chloride  75-01-4 | X | X | X | X | X | X | X^a^ | X | NIOSH Pocket Guide |
| 6:2 FTSHA  88992-45-4 | — | X | X | — | — | — | — | — | None |
| 6:2 FTSAS  88992-47-6 | — | — | X | — | — | — | — | — | None |
| 6:2 FTSA  27619-97-2 | — | X | X | — | — | — | — | — | None |
| 6:2 FTSA-PrB  34455-29-3 | — | X | X | — | — | — | — | — | None |
| 6:2 FTNO  80475-32-7 | — | X | X | — | — | — | — | — | None |
| 2,3,7,8-TCDD  1746-01-6 | X | — | X | X | X | X | — | X | Alaska DEC; WHO Factsheet; NRC Evaluation of EPA Reassessment |

6:2 FTNO = 6:2 fluorotelomer sulfonamido amine oxide; 6:2 FTSHA = 6:2 fluorotelomer thiohydroxyammonium; 6:2 FTSAS = 6:2 fluorotelomermercap-toalkylamido sulfonate; 6:2 FTSA = 6:2 fluorotelomer sulfonic acid; 6:2 FTSA-PrB = 6:2 fluorotelomer sulfonamide alkylbetaine; AEGL = Acute Exposure Guideline Levels for Airborne Chemicals; ATSDR = Agency for Toxic Substances and Disease Registry; CERHR = Center for the Evaluation of Risks to Human Reproduction; Comptox = Comptox Chemicals Dashboard; DEC = Department of Environmental Conservation; ECHA = European Chemicals Agency; EPA = Environmental Protection Agency; IARC = International Agency for Research on Cancer; INCHEM = Internationally Peer Reviewed Chemical Safety Information; IRIS = Integrated Risk Information System; NIOSH = National Institute for Occupational Safety and Health; NRC = National Research Council; NTP = National Toxicology Program; OECD = Organisation for Economic Co-operation and Development; OEHHA = Office of Environmental Health Hazard Assessment; OSHA = Occupational Safety and Health Administration; PPRTV = provisional peer-reviewed toxicity values; TCDD = tetrachlorodibenzo-p-dioxin; WHO = World Health Organization.

X signifies that a report, assessment, or other data were identified from the source.

— signifies that a report, assessment, or other data were not identified from the source.

^a^NTP Report on Carcinogens [[13](#_ENREF_13)]

^b^NTP Technical Report

^c^NTP toxicology studies

Table S5 Detailed Findings from Phase 1 Authoritative Source Reviews for 16 Primary East Palestine Chemicals of Interest

| Chemicals (CASRN) | Cancer | Nervous | Immune | Developmental | Reproductive | Other Organs | Skin Sensitization | Skin Irritation | Eye Irritation | Respiratory Irritation |
| --- | --- | --- | --- | --- | --- | --- | --- | --- | --- | --- |
| Acrolein  (107-02-8) | IARC: Probably carcinogenic  Gap: Human | Gap: Suggestive, no conclusion | Gap: Suggestive, no conclusion | No risk or concern:  Guideline study  Positive effects at doses causing maternal toxicity in animal studies | Low risk or concern:  Guideline study  Positive effects at high doses | Cardio & metabolic: Suggestive  Stomach irritant – ATSDR MRL [[14](#_ENREF_14)]: 0.004 mg/kg-day (oral; mice) | Suggestive, no conclusion | Category 1B | Category 1  OEHHA REL [[15](#_ENREF_15)]: 0.0025 mg/m^3^ (acute; humans) | EPA IRIS RfC [[16](#_ENREF_16)]: 2e-05 mg/m^3^* (rodents) |
| Butyl acrylate  (141-32-2) | IARC: Unclassifiable  Inadequate evidence from animal studies (negative) [[17](#_ENREF_17)] | Gap: No or few studies | Gap: No or few studies | No risk or concern: Guideline study  Positive effects at doses causing maternal toxicity in animal studies | No risk or concern: Guideline study | Hepatic: Suggestive, no conclusion | Category 1 | Category 2 | Category 2 | ECHA DNEL [[18](#_ENREF_18)]: 11 mg/m^3^* (inhalation; rats) |
| Ethylene glycol monobutyl ether  (EGBE or 2-Butoxyethanol)  (111-76-2) | IARC: Unclassifiable  Hemangiosarcoma and forestomach in animals [[19](#_ENREF_19)]  Gap: Human | Gap: Suggestive, no conclusion | Gap: Suggestive, no conclusion | Low risk or concern  Positive effects at doses causing maternal toxicity in animal studies | Low risk or concern:  Positive effects at high doses | Hemotoxicant   - ATSDR MRL [[20](#_ENREF_20)]: 0.97 mg/m^3^ (chronic, inhalation; humans) - EPA IRIS RfD [[21](#_ENREF_21)]: 0.100 mg/kg-day (oral; rats and mice)   Hepatic – ATSDR MRL *ATSDR [*[*20*](#_ENREF_20)*]***:** 0.07 mg/kg-day* (intermediate, oral; rats) | No or low concern | Category 2 | Category 2  OEHHA REL [[22](#_ENREF_22)]: 4.7 mg/m^3^ (acute; respiratory and eye irritation in humans) | OEHHA REL [[22](#_ENREF_22)]: 0.082 mg/m^3^ (chronic; humans) |
| 2-Ethylhexyl acrylate  (103-11-7) | IARC: Possibly carcinogenic to humans  Skin in animal studies [[4](#_ENREF_4)] Gap: Human | Gap: No or few studies | Gap: No or few studies | No or low concern | No or low concern | Gastro, renal, cardio, hemo, hepatic – Gap: No or few studies | Category 1 | Category 2 | Category 2 | May cause irritation |
| Benzene  (71-43-2) | NTP RoC and IARC: Known human carcinogen  Acute myeloid leukemia, other acute non-lymphocytic leukemia in humans  EPA CSF [[23](#_ENREF_23)]: 0.015 mg/kg-day* (humans) | Evidence indicates neurotoxicity from high exposure (e.g., workplace) in humans | ATSDR MRL *[*[*24*](#_ENREF_24)*]*: 0.00639 mg/m^3^ (inhalation; humans) | Causes developmental hematotoxicity in animals  OEHHA REL [[25](#_ENREF_25)]: 0.027 mg/m^3^ (inhalation; mice) | May harm the reproductive system (limited evidence) | Hemo: Changes in cellularity; bone marrow depression   - ATSDR MRL [[24](#_ENREF_24)]: 0.0003 mg/kg-day* (oral; humans) - OEHHA REL [[25](#_ENREF_25)]*:* 0.003 mg/m^3^* (inhalation; humans) | No or low concern | Category 2 | Category 2 | Gap: Suggestive, no conclusion |
| Hydrogen chloride  (7647-01-0) | IARC: Unclassifiable  Inadequately designed animal studies | Gap: No or few studies | Gap: No or few studies | Gap: No or few studies | Gap: No or few studies | Renal: Suggestive | No or low concern | Category 1 | Category 1  OEHHA REL [[26](#_ENREF_26)]: 2.1 mg/m^3^ (humans) | EPA IRIS RfC [[27](#_ENREF_27)]: 0.002 mg/m^3^* (rats) |
| Phosgene gas  (75-44-5) | Gap: No or few studies | Gap: No or few studies | Immune in lung;  Suggestive, no conclusion | Gap: No or few studies | Gap: No or few studies | Respiratory:  INCHEM [[28](#_ENREF_28)]  Pulmonary edema >600 mg/m^3^ (inhalation; humans)  EPA IRIS RfC [[29](#_ENREF_29)]: 3.00e-4 mg/m^3^* (rats) | Gap: No or few studies | Category 1 | Category 1 | Cough and throat irritation |
| Vinyl chloride  (75-01-4) | NTP RoC and IARC: Known human carciogen  Angiosarcoma of the liver and hepatocellular carcinoma in humans  ECHA DMEL [[30](#_ENREF_30)]: 0.002 mg/m^3^* (inhalation; humans) | Presumed for humans | Suspected for humans | Suspected for humans  ATSDR MRL [[31](#_ENREF_31)]: 1.30 mg/m**^3^** (inhalation; mice) | Gap: No or few studies | Hepatic: Presumed for humans   - EPA IRIS RfD [[32](#_ENREF_32)]: 0.003 mg/kg-day* (rats) - ATSDR MRL [[31](#_ENREF_31)]: 0.05 mg/m**^3^** (inhalation; rats) | Category 1 | Category 2 | OEHHA REL [[33](#_ENREF_33)]: 180 mg/m^3^ (inhalation; humans) | OEHHA REL [[33](#_ENREF_33)]: 180 mg/m^3^ (inhalation; humans) |
| Diethylene glycol  (111-46-6) | No carcinogenic potential | Gap: Suggestive, no conclusion | Gap: No or few studies | No or low concern  Positive effects at doses causing maternal toxicity in animal studies | No or low concern  Positive effects at high doses | Renal: Suggestive, no conclusion | No or low concern | No or low concern | Negative studies | Gap: Suggestive under certain exposure conditions |
| Dipropylene glycol  (25265-71-8) | No carcinogenic potential | Gap: No or few studies | Gap: No or few studies | No or low concern | No or low concern | Gap: No or few studies | No or low concern | Category 3 | Low confidence because of conflicting sources: Category 2A and null | Gap: No or few studies |
| Isobutylene  (115-11-7) | Thyroid follicular cell carcinomas in animals  Gap: Human | Gap: Suggestive, no conclusion | Gap: No or few studies | No or low concern | No or low concern | Gap: No or few studies | Gap: No or few studies | Gap: No or few studies | Gap: No or few studies | Nasal irritation at high doses in animals |
| Polypropylene glycol  (25322-69-4) | Gap: No or few studies | Gap: Suggestive | Gap: No or few studies | Gap: No or few studies | Gap: No or few studies | Cardio: Suggestive, no conclusion | No or low concern | Suggestive, no conclusion | Category 2A | No or low concern |
| 1,2 Propylene glycol  (57-55-6) | Not likely carcinogenic | Gap: No or few studies | Gap: No or few studies | No or low concern | No or low concern | Hemo**:** Effects (not severe) and hyperglycemia in animals;  EPA Provisional RfD from 1971 [[34](#_ENREF_34)]: 20 mg/kg-day* (rats) | No or low concern | Unclear; may be irritant at high doses | No or low concern | Suggestive^a^ |
| Petroleum lube oil  (64742-58-1) | Gap: No or few studies | Gap: No or few studies | Gap: No or few studies | Gap: No conclusions | Gap: No or few studies | Gap: No or few studies | Gap: No or few studies | Category 2 | Category 2 | Gap: No or few studies |
| Polyethylene  (9002-88-4) | IARC: Unclassifiable  Inadequately designed animal studies [[35](#_ENREF_35)] | Gap: No or few studies | Gap: No or few studies | Gap: No or few studies | Gap: No or few studies | Gap: No or few studies | Gap: No or few studies | Gap: No or few studies | Gap: No or few studies | Gap: No or few studies |
| Polyvinyl alcohol  (9002-89-5) | IARC: Unclassifiable  Conflicting animal evidence [[35](#_ENREF_35)] | Gap: No or few studies | Gap: No or few studies | Gap: No or few studies | Gap: No or few studies | Gap: No or few studies | Gap: No or few studies | Gap: No or few studies | Gap: No or few studies | Gap: No or few studies |

*Indicates cancer slope factors or values that were lowest across health systems for a given exposure route.

Values for the inhalation route were converted to mg/m^3^ for ease of comparison.

^a^ATSDR provides an MRL value for respiratory irritation following 1,2 propylene glycol exposure. “Suggestive” was selected because the overall evidence was inconclusive across sources.

ATSDR = Agency for Toxic Substances and Disease Registry; Cardio = cardiovascular; CASRN = Chemical Abstracts Service Registry Number; CSF = cancer slope factor; DMEL = derived minimal effect level; DOD = Department of Defense; ECHA = European Chemicals Agency; EPA = Environmental Protection Agency; Gastro = gastrointestinal; Hemo = hematological; IARC = International Agency for Research on Cancer; INCHEM = Internationally Peer Reviewed Chemical Safety Information; IRIS = Integrated Risk Information System; MEG = military exposure guidelines; MRL = minimal risk level; NIOSH = National Institute for Occupational Safety and Health; NRC = National Research Council; NTP RoC = National Toxicology Program Report on Carcinogens; OEHHA = Office of Environmental Health Hazard Assessment; PPRTV = provisional peer-reviewed toxicity values; REL = recommended exposure limit; RfC = reference concentration; RfD = reference dose; TWA = time-weighted average.

Table S6 Detailed Findings from Phase 1 Authoritative Source Reviews for Potentially Related Chemicals

| Chemical (CASRN) | Cancer | Nervous | Immune | Developmental | Reproductive | Other Organs | Skin Sensitization | Skin Irritation | Eye Irritation | Respiratory Irritation |
| --- | --- | --- | --- | --- | --- | --- | --- | --- | --- | --- |
| 6:2 FTSHA  (88992-45-4) | Gap: No or few studies | Gap: No or few studies | Gap: No or few studies | Presumed (Category 1B) in guideline animal study | Presumed (Category 1B) in guideline animal study | Gap: No or few studies for specfiic organs  General Toxicity: ECHA DNELs [[36](#_ENREF_36)]   - Oral: 20.8 µg/kg bw-day* - Dermal: 52.1 µg/kg bw-day - Inhalation: 36.3 µg/m^3^* | Not sensitizing (in vitro); sensitizing in guideline animal study | No or low concern | Category 1 | Gap: No or few studies |
| 6:2 FTSAS  (88992-47-6) | Gap: No or few studies | Gap: No or few studies | Gap: No or few studies | Gap: No or few studies | Gap: No or few studies | Gap: No or few studies | Gap: No or few studies | Gap: No or few studies | Gap: No or few studies | Gap: No or few studies |
| 6:2 FTSA  (27619-97-2) | Gap: No or few studies | Gap: No or few studies | Gap: No or few studies | No or low concern | No or low concern | Gap: No or few studies | No or low concern | Category 1B | Category 1 | Gap: No or few studies |
| 6:2 FTSA-PrB  (34455-29-3) | Gap: No or few studies | Gap: No or few studies | Gap: No or few studies | Gap: No or few studies | Gap: No or few studies | Gap: No or few studies | No or low concern | No or low concern | Suggestive: Some irritation in guideline study (reversed in 24 hours) | Gap: No or few studies |
| 6:2 FTNO  (80475-32-7) | Gap: No or few studies | No or low concern | Gap: No or few studies | No or low concern | No or low concern | Cardiovascular: Suggestive, some decreased serum lipids in guideline studies  Hemopoietic: No or low concern | No or low concern | No or low concern | No or low concern | Gap: No or few studies |
| 2,3,7,8-TCDD  (1746-01-6) | NTP RoC and IARC: Known human carcinogen  IARC humans (all cancers, STS, NHL) [[37](#_ENREF_37)] | Nervous effects in animals;  Alaska DEC MRL [[38](#_ENREF_38)]: 1 × 10^−9^ mg/kg/day (oral, monkeys) | Immune suppression in animals;  ATSDR MRL [[39](#_ENREF_39)]: 2 × 10−^4^ mg/kg/day (oral, mice) | Casuses developmental toxicity;  RfD: 7 × 10^−10^ mg/kg/day* (oral, humans) [[40](#_ENREF_40)] | Casuses reproductive toxicity;  EPA IRIS RfD [[40](#_ENREF_40)]: 7 × 10^−10^ mg/kg/day* (oral, humans) | OEHHA PHG [[41](#_ENREF_41)] : 0.002 ng/L (animals)  Hepatic, respiratory, hematological:  OEHHA REL [[42](#_ENREF_42)] : 1 x 10^−8^ mg/kg-day (oral, animals)  OEHHA REL [[42](#_ENREF_42)]: 4 × 10^−5^ µg/m^3^-day* (inhalation, animals)  Cardiovascular: Suggestive | Gap: No or few studies | Category 2 | Category 2 | Gap: No or few studies |

*Indicates cancer slope factors or values that were lowest across health systems for a given exposure route.

6:2 FTNO = 6:2 fluorotelomer sulfonamido amine oxide; 6:2 FTSHA = 6:2 fluorotelomer thiohydroxyammonium; 6:2 FTSAS = 6:2 fluorotelomermercap-toalkylamido sulfonate; 6:2 FTSA = 6:2 fluorotelomer sulfonic acid; 6:2 FTSA-PrB = 6:2 fluorotelomer sulfonamide alkylbetaine; ATSDR = Agency for Toxic Substances and Disease Registry; CASRN = Chemical Abstracts Service Registry Number; DEC = Department of Environmental Conservation; EPA = Environmental Protection Agency; Hemo = hematological; IARC = International Agency for Research on Cancer; IRIS = Integrated Risk Information System; MRL = minimal risk level; NHL = non-Hodgkin lymphoma; NTP RoC = National Toxicology Program Report on Carcinogens; OEHHA = Office of Environmental Health Hazard Assessment; PFAS = per- and polyfluoroalkyl substances; PHG = public health goal; REL = recommended exposure limit; RfD = reference dose; STS = soft tissue sarcoma; TCDD = tetrachlorodibenzo-p-dioxin.

Table S7. Summary of 2-Butoxyethanol Immune Studies

| Endpoint,  Identified Studies | Summary of Findings |
| --- | --- |
|  | *Functional Immune Findings* |
| Antibody Response (Functional Assay)  3 *primary* articles in animals [[43-45](#_ENREF_43)] | No significant findings in male and female rats exposed via drinking water for 21 days at doses ranging from 1,600 to 6,000 ppm [[43](#_ENREF_43)] or in male rats dosed by oral gavage for 2 days after immunization at doses ranging from 50 to 100 mg/kg/day [[44](#_ENREF_44)]. The authors attributed signficant findings at 200 mg/kg/day to hematotoxicity and mortality [[44](#_ENREF_44)].  No significant effects to IgM plaque-forming cell response to sheep red blood cells in female mice exposed topically for 4 days at doses ranging from 100 to 1,500 mg/kg/day [[45](#_ENREF_45)].  **Endpoint Summary:** No significant findings in two studies of rats at nontoxic doses and in one study of female mice. |
| Autoimmune Response  1 *primary* article in animals [[46](#_ENREF_46)] | A study of male rats following a single intraperitoneal injection of 20 mg/kg-bw reported significantly increased in vitro agglutination to the rat’s own red blood cells collected prior to study initiation, suggestive of autoimmune response [[46](#_ENREF_46)]. The study also reported histopathological changes in the thymus, indicative of organ stress response (see Observational Findings below).  **Endpoint Summary:** Increased autoimmune response in one study of male rats that also reported histopathological thymus effects. |
| Natural Killer (NK) Cell Activity  2 *primary* articles in animals [[43](#_ENREF_43), [45](#_ENREF_45)] | Significant increase in NK cytotoxic responses in male and female rats exposed via drinking water for 21 days at doses ranging from 1,600 to 6,000 ppm [[43](#_ENREF_43)].  No effect on NK cytotoxic activity in female mice exposed topically for 4 days at doses ranging from 100 to 1,500 mg/kg/day [[45](#_ENREF_45)].  **Endpoint Summary:** Increased responses reported in one rat study; no change in one mouse study exposed to lower doses. |
| Delayed-type Hypersensitivity (DTH) Response  1 *primary* article in animals [[43](#_ENREF_43)] | No significant effects on the DTH response in male and female rats exposed via drinking water for 21 days at doses ranging from 1,600 to 6,000 ppm [[43](#_ENREF_43)].  **Endpoint Summary:** No significant findings in one study in rats. |
| Cytotoxic T Lymphocyte (CTL) Activity  1 *primary* article in animals [[45](#_ENREF_45)] | No signficant response to cytotoxic T-cell response (primarily CD8+ cells) [[45](#_ENREF_45)].  **Endpoint Summary:** No significant response in one study in mice. |
| Mixed Lymphocyte Response (MLR)  1 *primary* article in animals [[45](#_ENREF_45)] | Signficant reduction of the MLR to allogenic antigen in female mice exposed topically for 4 days at doses ranging from 100 to 1,500 mg/kg/day [[45](#_ENREF_45)].  **Endpoint Summary:** Reduced MLR in one study in mice. |
| Nonspecific Mitogenic Response (Lymphoproliferative Assays)  1 *primary* article in animals [[45](#_ENREF_45)] | B-cell mitogens: No significant effects to splenic B-cell lymphoproliferate response to lipopolysaccharide in female mice exposed topically for 4 days at doses ranging from 100 to 1,500 mg/kg/day [[45](#_ENREF_45)].  T-cell mitogens: Significant reduction of splenic T-cell lymphoproliferate response to Con a in female mice exposed topically for 4 days at doses ranging from 100 to 1,500 mg/kg/day [[45](#_ENREF_45)].  **Endpoint Summary:** Reduced lymphoproliferative response results in T-cell assays in one study in mice. No effects in B-cell assays. |
|  | Observational Immune Findings |
| Cytokines  1 *primary* article in animals [[43](#_ENREF_43)] | No significant findings in interleukin-2 (IL-2) or interferon (IFN) production in male and female rats exposed via drinking water for 21 days at doses ranging from 1,600 to 6,000 ppm [[43](#_ENREF_43)].  **Endpoint Summary Across Observational Immune Findings:** Histopathology of the thymus was reported in one study that also found increased autoimmune response in rats. Other observational findings were largely mixed and lacked consistency, as studies reporting effects varied in design, route of exposure, and species. The study heterogeneity limits the ability to draw conclusions about observational immune findings. |
| Immune Organ Histopathology and Cellularity  9 *primary* articles in animals [[43](#_ENREF_43), [46-53](#_ENREF_46)] | One study reporting autoimmune response following exposure also observed involution of the thymus [[46](#_ENREF_46)]. Another study observed changes in thymic cellularity, including transient lymphocyte depletion in the cortex and increased lymphocytes in the medulla [[50](#_ENREF_50)].  Two studies reported histopathological changes in the spleen, including a significant decrease in relative volume of white pulp [[46](#_ENREF_46)] and white atrophic pulp after death [[48](#_ENREF_48)].  No histopathological changes were reported in the thymus or lymph nodes in other studies of rats, guinea pigs, mice, and rabbits of various designs. Most studies provided observational data only and did not include functional measures.  **Endpoint Summary:** See Cytokines above. |
| Immune Organ Weight  6 *primary* articles in animals [[43](#_ENREF_43), [45](#_ENREF_45), [47](#_ENREF_47), [50](#_ENREF_50), [52](#_ENREF_52), [54](#_ENREF_54)] | Mixed results were reported for relative and absolute thymus weights across studies of various designs, exposure routes, and measurement timings.  **Endpoint Summary:** See Cytokines above. |
| White Blood Cell Counts and Differentials  1 *primary* article in humans [[55](#_ENREF_55)]  7 *primary* articles in animals [[49](#_ENREF_49), [50](#_ENREF_50), [52](#_ENREF_52), [54](#_ENREF_54), [56-58](#_ENREF_56)] | Mixed results were reported for white blood cell changes, including total leukocyte counts and differentials, with studies reporting significant increases, significant decreases, and no significant changes across a variety of study designs, exposure routes, and measurement timings.  Lymphocyte counts were mixed across one study in humans and six studies in animals. Some studies of longer duration reported significant findings (increases and decreases) at earlier timepoints that were not reported at later timepoints.  Of studies reporting neutrophil counts, several reported significantly increased counts at various time points that were not observed at later time points. Other studies reported decreases or no change in counts.  **Endpoint Summary:** See Cytokines above. |

CTL = cytotoxic T lymphocyte; DTH = delayed-type hypersensitivity; IFN = interferon; IgM = immunoglobulin M; IL-2: interleukin-2; MLR = mixed lymphocyte response; NK = natural killer.

Table S8. Summary of 2-Butoxyethanol Nervous Studies

| Endpoint,  Identified Studies | Summary of Findings |
| --- | --- |
| Brain and Nerve Histopathology  5 *primary* articles in animals [[47](#_ENREF_47), [49](#_ENREF_49), [52](#_ENREF_52), [56](#_ENREF_56), [59](#_ENREF_59)] | No lesions or histopathological changes were noted in the brains or nervous tissue of rats, mice, or rabbits exposed via oral, inhalation, and dermal routes [[47](#_ENREF_47), [49](#_ENREF_49), [52](#_ENREF_52), [56](#_ENREF_56), [59](#_ENREF_59)].  **Endpoint Summary:** No histopathological changes identified. |
| Motor and Vestibular Deficits  3 *primary* articles in animals [[56](#_ENREF_56), [60](#_ENREF_60), [61](#_ENREF_61)] | Loss of coordination was observed in male and female rats after inhalation exposure to 523 and 867 ppm, respectively, for 4 hours [[56](#_ENREF_56)].  Male albino rabbits showed loss of equilibrium and poor coordination from inhalation exposure for 7 hours/day for 1–2 days at ~400 ppm [[60](#_ENREF_60)].  Pregnant mice exposed via gavage at ≥1,500 mg/kg/day experienced lethargy and failure to right [[61](#_ENREF_61)].  **Endpoint Summary:** Signs of coordination and equilibrium loss in three animal studies. |
| Sensory Impacts  1 *primary* article in animals [[62](#_ENREF_62)] | Photoreceptor degeneration observed in 5/8 female rats following daily gavage exposure to 250 mg/kg-bw for 3 days [[62](#_ENREF_62)].  **Endpoint Summary:** Photoreceptor effects in one animal study. |
| Severe Nervous System Depression (Including Coma)  5 *case reports* in humans [[63-68](#_ENREF_63)] | Comas were observed in males and females ranging from 19 to 87 years old after ingestion of 2-butoxyethanol, largely from household cleaners, ranging in estimated dose from 391 mg/kg to 650 mg/kg [[63](#_ENREF_63), [64](#_ENREF_64), [66-68](#_ENREF_66)].  After ingesting a household cleaner containing 22% 2-butoxyethanol, an 18-year-old male experienced severe central nervous system depression, although this was not reported after a second ingestion event of the same cleaner [[69](#_ENREF_69)].  Two children (14 months and 2 years old) had no evidence of nervous system depression after ingestion event at estimated doses of 290 and 1,862 mg/kg, respectively [[65](#_ENREF_65)].  **Endpoint Summary:** Comas and severe nervous system depression observed in case reports only. |
| Dysautonomia  2 *case reports* in humans [[64](#_ENREF_64), [70](#_ENREF_70)] | A 19-year-old male with preexisting neurological conditions showed inhibited reflexes immediately following ingestion [[64](#_ENREF_64)].  A 16-month-old female was unable to open her eyes to voice immediately following ingestion; her gag and withdrawal reflexes were unaffected [[70](#_ENREF_70)].  **Endpoint Summary:** Impacts to reflexes observed in case reports only. |
| Sensory Impacts  1 *experimental study* in humans [[71](#_ENREF_71)] | Male and female volunteers reported disturbed taste sensation after inhalation at 113 and 195 ppm, respectively, for 4–8 hours in an experimental study [[71](#_ENREF_71)].  **Endpoint Summary:** Taste sensation effects in one human study. |
| Verbal Function  1 *case report* in humans [[64](#_ENREF_64)] | A 19-year-old male with preexisting neurological conditions was unable to speak beyond sounds 2 months after an ingestion event [[64](#_ENREF_64)].  **Endpoint Summary:** Verbal function effects in one case report. |

Supplemental Figures

Figure S1. Phased Approach Schematic


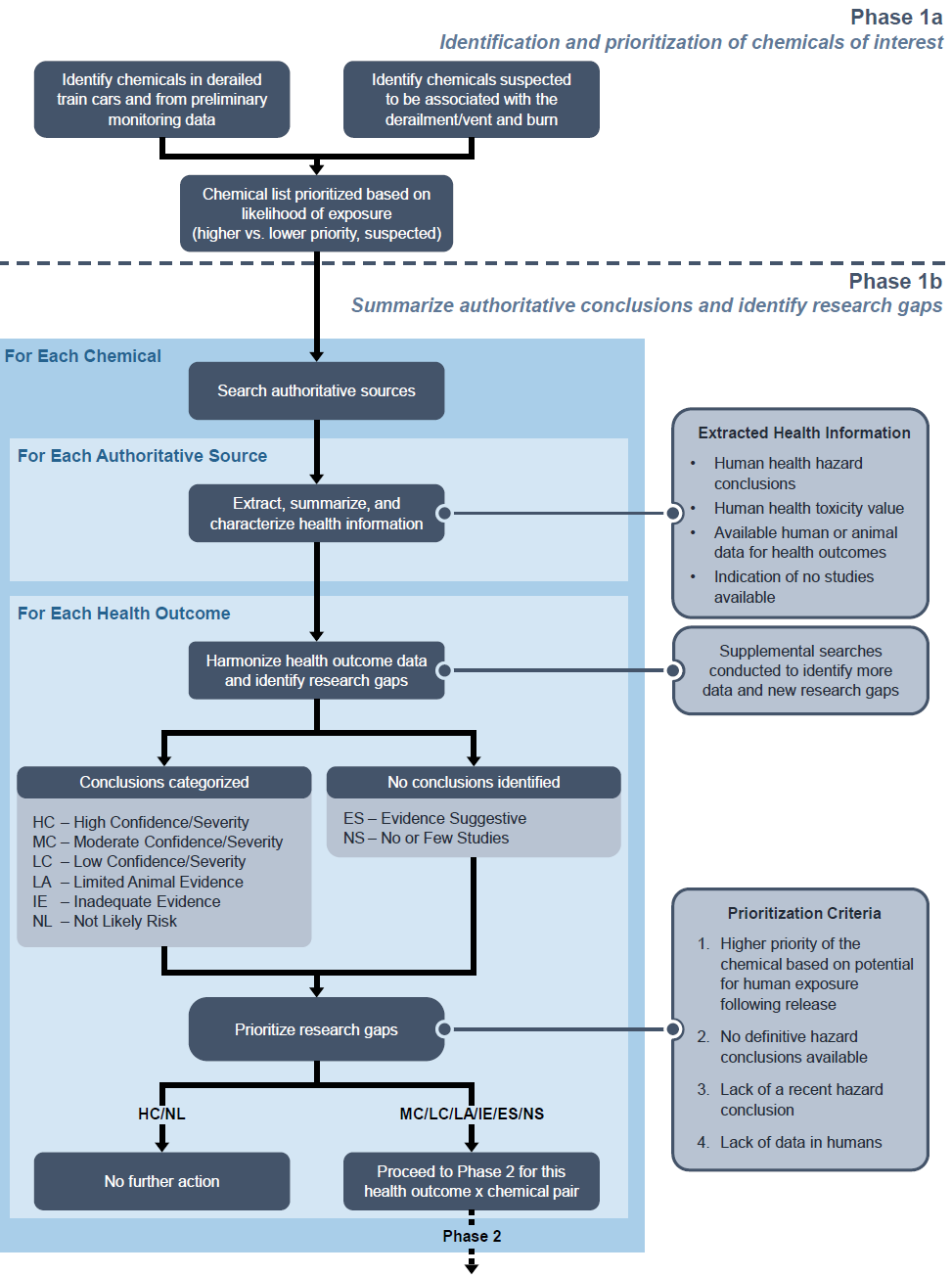


Schematic that outlines steps taken during Phase 1a and Phase 1b to identify and categorize chemicals, identify and categorize available hazard data, and prioritize chemical × health outcome pair data gaps for additional Phase 2 searching. HC = higher confidence or severity; MC = moderate confidence or severity; LC = lower confidence or severity; LA = limited animal evidence; IE = inadequate evidence; NL = not likely to be a risk; ES = evidence suggestive; NS = no or few studies

Figure S2. Categorization of Authoritative Source Findings by Confidence or Severity

**
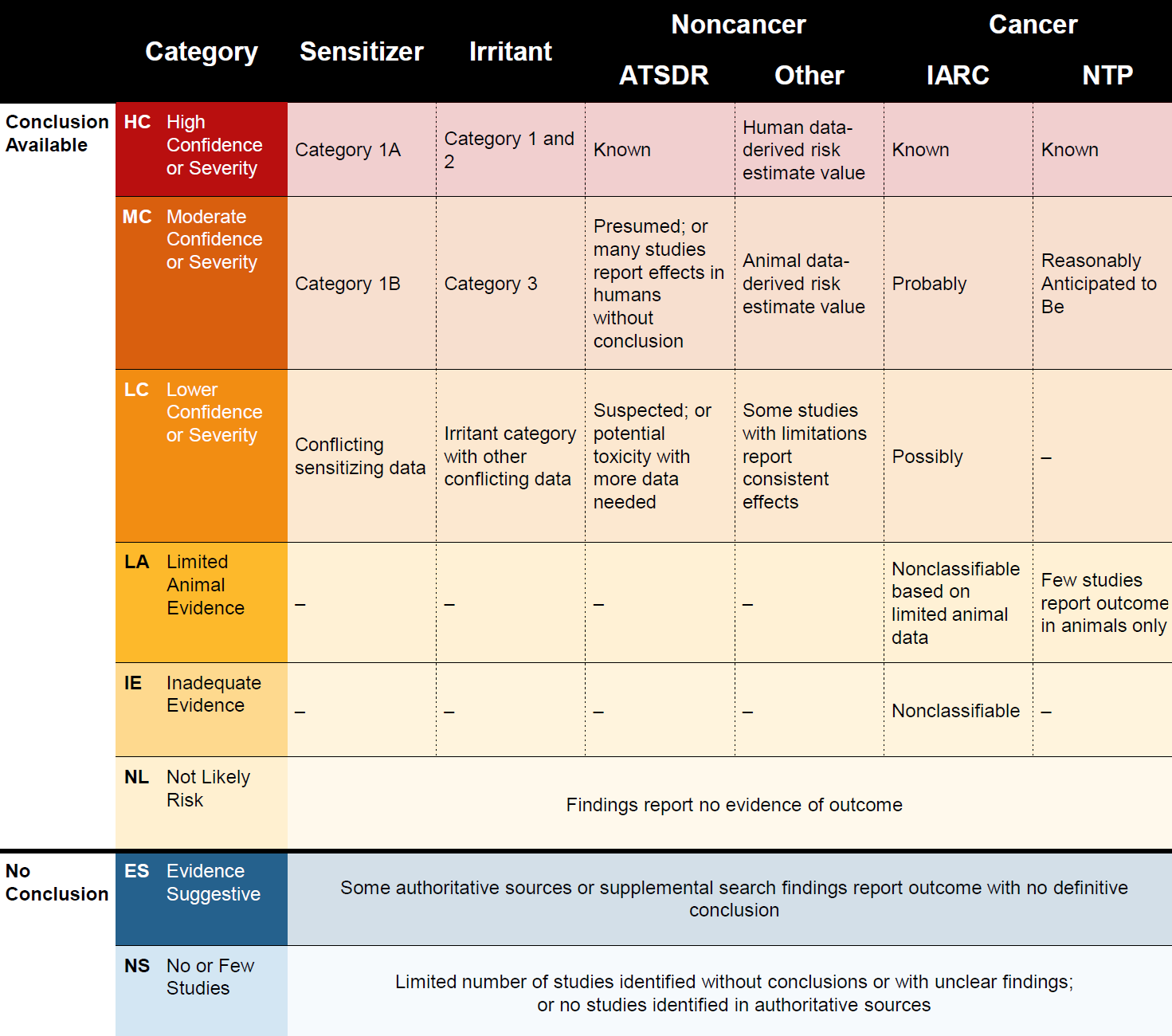
**

Mapping of authoritative source findings to categories used for each chemical × health outcome pair. Specific considerations were made for sensitization, irritation, cancer, and noncancer findings. ATSDR reviews did not consistently use conclusive language for moderate (MC) or lower confidence or severity (LC) categories. For benzene× immune, ATSDR’s 2024 draft Toxicological Profile [[24](#_ENREF_24)] reported a presumed health effect, but the pair was assigned HC due to the collective available evidence.

HC = higher confidence or severity; MC = moderate confidence or severity; LC = lower confidence or severity; LA = limited animal evidence; IE = inadequate evidence; NL = not likely to be a risk; ES = evidence suggestive; NS = no or few studies.

Figure S3. Summary of Phase 2 Literature Search and Screening for Select Chemicals


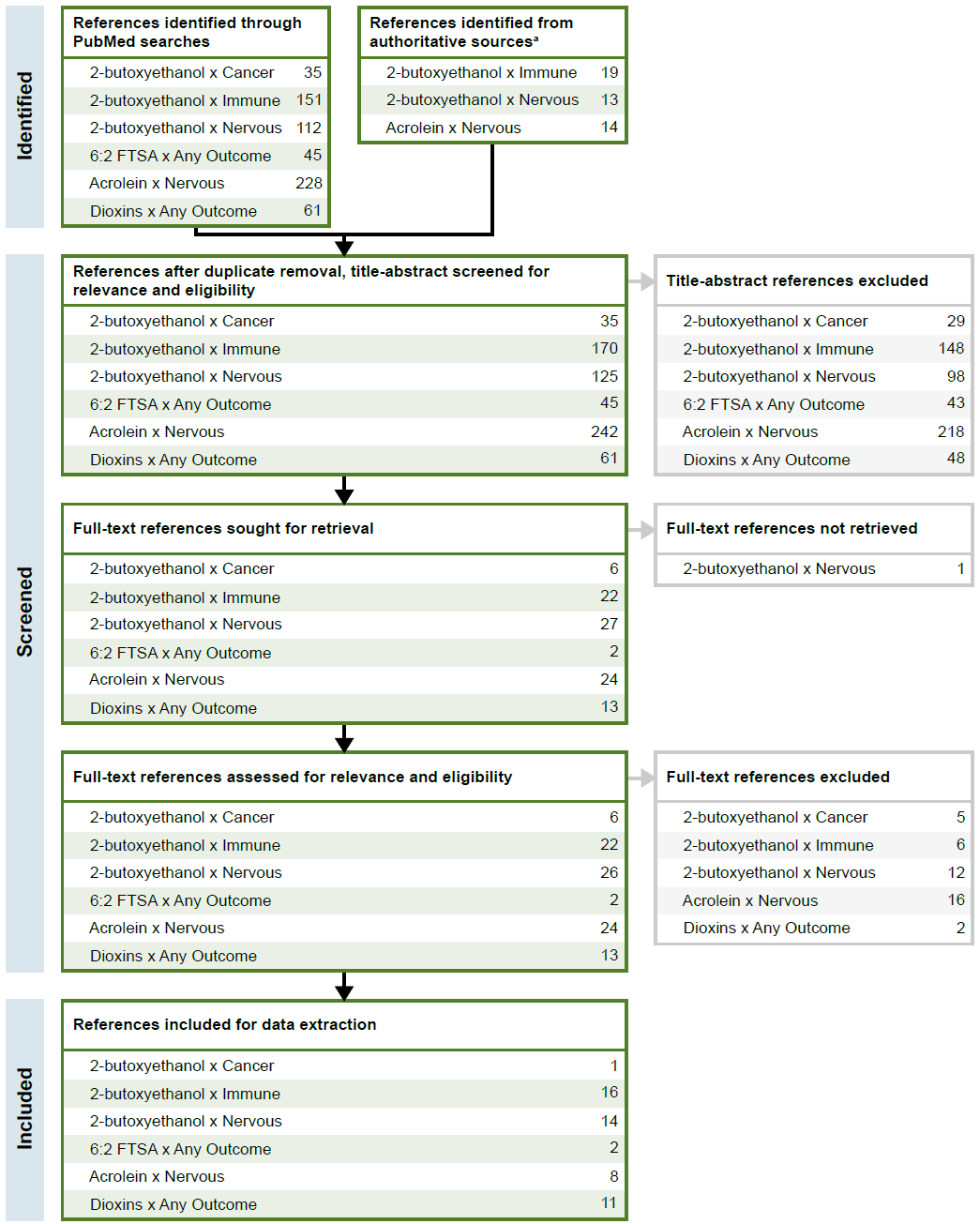


Flow chart illustrating selection of Phase 1 and Phase 2 literature assessed for scoping review for select chemicals.

Interactive figure and additional study details available on [Tableau](https://public.tableau.com/app/profile/division.of.translational.toxicology.dtt./viz/EastPalestineI-REFF_17025746048980/IREFF?publish=yes). This interactive figure is based on work by [Taylor et al. [72]](#_ENREF_72).

^a^ Studies identified in reviews from authoritative sources were screened for relevance and, when applicable, were integrated in syntheses with those identified in PubMed searches. No studies were identified from authoritative sources for two chemicals × outcome pairs (2-butoxyethanol × cancer; 6:2 FTSA × any outcome).

Figure S4. Infographic of the Rapid Review of East Palestine Chemicals

This infographic provides a summary of key information on the rapid review with the intention of communicating with the impacted East Palestine, OH community.

References

1. Sciome. SWIFT-Review search strategies. Research Triangle Park, NC: Sciome; 2023. <https://www.sciome.com/swift-review/searchstrategies/>. Accessed 1 Jun 2023.

2. U.S. Environmental Protection Agency (USEPA). Access Chemical Data Reporting data: 2020 CDR data. Washington, DC: U.S. Environmental Protection Agency; 2020. <https://www.epa.gov/chemical-data-reporting/access-chemical-data-reporting-data#2020>. Accessed 7 Nov 2023.

3. International Agency for Research on Cancer (IARC). IARC monographs on the identification of carcinogenic hazards to humans, volume 128: Acrolein, crotonaldehyde, and arecoline. Lyon, France: International Agency for Research on Cancer; 2021. <https://publications.iarc.fr/602>.

4. International Agency for Research on Cancer (IARC). IARC monographs on the evaluation of carcinogenic risks to humans, volume 122: Isobutyl nitrite, β-picoline, and some acrylates. Lyon, France: International Agency for Research on Cancer; 2019. <https://publications.iarc.fr/583>.

5. Agency for Toxic Substances and Disease Registry (ATSDR). Toxicological profiles. Atlanta, GA: U.S. Department of Health and Human Services, Public Health Service, Agency for Toxic Substances and Disease Registry; 2023. <https://www.atsdr.cdc.gov/toxprofiledocs/index.html>. Accessed 15 Aug 2023.

6. European Chemicals Agency (ECHA). Search for chemicals. Helsinki, Finland: European Chemicals Agency; 2023. <https://echa.europa.eu/information-on-chemicals>. Accessed 15 Aug 2023.

7. U.S. Environmental Protection Agency (USEPA). CompTox Chemicals Dashboard. Washington, DC: U.S. Environmental Protection Agency; 2023. <https://comptox.epa.gov/dashboard/>. Accessed 1 Jun 2023.

8. U.S. Environmental Protection Agency (USEPA). IRIS assessments. Washington, DC: U.S. Environmental Protection Agency; 2023. <https://iris.epa.gov/AtoZ/?list_type=alpha>. Accessed 15 Aug 2023.

9. Environment and Climate Change Canada (ECCC), Health Canada. Canadian Environmental Protection Act: Priority substances list. Ottawa, Ontario: Government of Canada; 2023. <https://www.canada.ca/en/environment-climate-change/services/canadian-environmental-protection-act-registry/substances-list/priority-list.html>. Accessed 15 Aug 2023.

10. International Agency for Research on Cancer (IARC). IARC monographs on the identification of carcinogenic hazards to humans. Lyon, France: International Agency for Research on Cancer; 2023. <https://monographs.iarc.who.int/monographs-available/>. Accessed 15 Aug 2023.

11. National Toxicology Program (NTP). NTP technical reports index. Research Triangle Park, NC: U.S. Department of Health and Human Services, National Institutes of Health, National Institute of Environmental Health Sciences, National Toxicology Program; 2023. <https://ntp.niehs.nih.gov/data/tr>. Accessed 15 Aug 2023.

12. Office of Environmental Health Hazard Assessment (OEHHA). Chemicals: Toxicity criteria on chemicals evaluated by OEHHA. Sacramento, CA: California Environmental Protection Agency, Office of Environmental Health Hazard Assessment; 2023. <https://oehha.ca.gov/chemicals>. Accessed 15 Aug 2023.

13. National Toxicology Program (NTP). Report on carcinogens, fifteenth edition. Research Triangle Park, NC: U.S. Department of Health and Human Services, Public Health Service, National Toxicology Program; 2021. <https://doi.org/10.22427/NTP-OTHER-1003>.

14. Agency for Toxic Substances and Disease Registry (ATSDR). Toxicological profile for acrolein. Atlanta, GA: U.S. Department of Health and Human Services, Public Health Service, Agency for Toxic Substances and Disease Registry; 2007. <https://www.atsdr.cdc.gov/ToxProfiles/tp124.pdf>.

15. Office of Environmental Health Hazard Assessment (OEHHA). Acrolein. Sacramento, CA: California Environmental Protection Agency, Office of Environmental Health Hazard Assessment; 2023. <https://oehha.ca.gov/chemicals/acrolein>. Accessed 15 Jun 2023.

16. U.S. Environmental Protection Agency (USEPA). Toxicological review of acrolein (CAS No. 107-02-8). Washington, DC: U.S. Environmental Protection Agency; 2003. EPA Report No. EPA/635/R-03/003. <https://iris.epa.gov/static/pdfs/0364tr.pdf>.

17. International Agency for Research on Cancer (IARC). n-Butyl acrylate. In: IARC Monographs on the Evaluation of Carcinogenic Risks to Humans, Volume 71: Re-evaluation of Some Organic Chemicals, Hydrazine and Hydrogen Peroxide. Lyon, France: International Agency for Research on Cancer; 1999. p. 359-366. <https://publications.iarc.fr/_publications/media/download/2294/ff59fbb2de7198b219c16e7ad6cdb37e0e7cf464.pdf>.

18. European Chemicals Agency (ECHA). Registration dossier: Butyl acrylate: Toxicological summary. Helsinki, Finland: European Chemicals Agency; 2023. <https://echa.europa.eu/registration-dossier/-/registered-dossier/15779/7/1>. Accessed 15 Jun 2023.

19. International Agency for Research on Cancer (IARC). IARC monographs on the evaluation of carcinogenic risks to humans, volume 88: Formaldehyde, 2-butoxyethanol and 1-tert-butoxypropan-2-ol. Lyon, France: International Agency for Research on Cancer; 2006. <https://publications.iarc.fr/106>.

20. Agency for Toxic Substances and Disease Registry (ATSDR). Toxicological profile for 2-butoxyethanol and 2-butoxyethanol acetate. Atlanta, GA: U.S. Department of Health and Human Services, Public Health Service, Agency for Toxic Substances and Disease Registry; 1998. <https://www.atsdr.cdc.gov/ToxProfiles/tp118.pdf>.

21. U.S. Environmental Protection Agency (USEPA). Toxicological review of ethylene glycol monobutyl ether (EGBE) (CAS No. 111-76-2). Washington, DC: U.S. Environmental Protection Agency; 2010. EPA Report No. EPA/635/R-08/006F. <https://iris.epa.gov/static/pdfs/0500tr.pdf>.

22. Office of Environmental Health Hazard Assessment (OEHHA). Ethylene glycol mono‐n‐butyl ether reference exposure levels: Technical support document for the derivation of noncancer reference exposure levels: Appendix D1. Sacramento, CA: California Environmental Protection Agency, Office of Environmental Health Hazard Assessment; 2018. <https://oehha.ca.gov/media/downloads/crnr/finalegberel050418.pdf>.

23. U.S. Environmental Protection Agency (USEPA). Integrated Risk Information System (IRIS) chemical assessment summary: Benzene; CASRN 71-43-2. Washington, DC: U.S. Environmental Protection Agency; 2003. <https://iris.epa.gov/static/pdfs/0276_summary.pdf>.

24. Agency for Toxic Substances and Disease Registry (ATSDR). Toxicological profile for benzene: Draft for public comment, October 2024. Atlanta, GA: U.S. Department of Health and Human Services, Public Health Service, Agency for Toxic Substances and Disease Registry; 2024. <https://www.atsdr.cdc.gov/toxprofiles/tp3.pdf>.

25. Office of Environmental Health Hazard Assessment (OEHHA). TSD for noncancer RELS: Appendix D. Individual acute, 8-hour, and chronic reference exposure level summaries. Sacramento, CA: California Environmental Protection Agency, Office of Environmental Health Hazard Assessment; 2014. <https://oehha.ca.gov/media/downloads/crnr/appendixd1final.pdf>.

26. Office of Environmental Health Hazard Assessment (OEHHA). Hydrogen chloride. Sacramento, CA: California Environmental Protection Agency, Office of Environmental Health Hazard Assessment; 2023. <https://oehha.ca.gov/chemicals/hydrogen-chloride>. Accessed 15 Jun 2023.

27. U.S. Environmental Protection Agency (USEPA). Integrated Risk Information System (IRIS) chemical assessment summary: Hydrogen chloride; CASRN 7647-01-0. Washington, DC: U.S. Environmental Protection Agency; 2003. <https://iris.epa.gov/static/pdfs/0396_summary.pdf>.

28. International Programme on Chemical Safety (IPCS). Phosgene: Health and safety guide. Geneva, Switzerland: World Health Organization; 1998. <https://www.inchem.org/documents/hsg/hsg/hsg106.htm>.

29. U.S. Environmental Protection Agency (USEPA). Toxicological review of phosgene (CAS No. 75-44-5). Washington, DC: U.S. Environmental Protection Agency; 2005. EPA Report No. EPA/635/R-06/001. <https://iris.epa.gov/static/pdfs/0487tr.pdf>.

30. European Chemicals Agency (ECHA). Registration dossier: Chloroethylene: Toxicological summary. Helsinki, Finland: European Chemicals Agency; 2023. <https://echa.europa.eu/de/registration-dossier/-/registered-dossier/16163/7/1>. Accessed 15 Jun 2023.

31. Agency for Toxic Substances and Disease Registry (ATSDR). Toxicological profile for vinyl chloride. Atlanta, GA: U.S. Department of Health and Human Services, Public Health Service, Agency for Toxic Substances and Disease Registry; 2024. <https://www.atsdr.cdc.gov/ToxProfiles/tp20.pdf>.

32. U.S. Environmental Protection Agency (USEPA). Toxicological review of vinyl chloride (CAS No. 75-01-4). Washington, DC: U.S. Environmental Protection Agency; 2000. EPA Report No. EPA/635R-00/004. <https://iris.epa.gov/static/pdfs/1001tr.pdf>.

33. Office of Environmental Health Hazard Assessment (OEHHA). Vinyl chloride. Sacramento, CA: California Environmental Protection Agency, Office of Environmental Health Hazard Assessment; 2023. <https://oehha.ca.gov/chemicals/vinyl-chloride>. Accessed 15 Jun 2023.

34. U.S. Environmental Protection Agency (USEPA). Provisional peer reviewed toxicity values for propylene glycol (CASRN 57-55-6). Cincinnati, OH: U.S. Environmental Protection Agency, Office of Research and Development, National Center for Environmental Assessment, Superfund Health Risk Technical Support Center; 2008. <https://hhpprtv.ornl.gov/issue_papers/PropyleneGlycol.pdf>.

35. International Agency for Research on Cancer (IARC). IARC monographs on the evaluation of the carcinogenic risk of chemicals to humans, volume 19: Some monomers, plastics and synthetic elastomers, and acrolein. Lyon, France: International Agency for Research on Cancer; 1979. <https://publications.iarc.fr/37>.

36. European Chemicals Agency (ECHA). Registration dossier: 1-Propanaminium, 2-hydroxy-N,N,N-trimethyl-3-[(3,3,4,4,5,5,6,6,7,7,8,8,8-tridecafluorooctyl)thio]-, chloride (1:1): Toxicological summary. Helsinki, Finland: European Chemicals Agency; 2023. <https://echa.europa.eu/fi/registration-dossier/-/registered-dossier/25974/7/1>. Accessed 15 Jun 2023.

37. International Agency for Research on Cancer (IARC). 2,3,7,8-Tetrachlorodibenzo-para-dioxin, 2,3,4,7,8-pentachlorodibenzofuran, and 3,3′,4,4′,5-pentachlorobiphenyl. In: IARC Monographs on the Evaluation of Carcinogenic Risks to Humans, Volume 100F: Chemical Agents and Related Occupations. Lyon, France: International Agency for Research on Cancer; 2012. p. 339-378. <https://publications.iarc.fr/_publications/media/download/5294/54fa5d8636ffa820655319ed30187a23c866f4c6.pdf>.

38. Alaska Department of Environmental Conservation. 2,3,7,8-Tetrachlorodibenzo-p-dioxin (dioxin): #1 minimum risk level. Juneau, AK: Department of Environmental Conservation; 2003. <https://dec.alaska.gov/media/8817/tetrachlorodibenzodioxin1.pdf>.

39. Agency for Toxic Substances and Disease Registry (ATSDR). Toxicological profile for chlorinated dibenzo-p-dioxins. Atlanta, GA: U.S. Department of Health and Human Services, Public Health Service, Agency for Toxic Substances and Disease Registry; 1998. <https://www.atsdr.cdc.gov/toxprofiles/tp104.pdf>.

40. U.S. Environmental Protection Agency (USEPA). Integrated Risk Information System (IRIS) chemical assessment summary: 2,3,7,8-tetrachlorodibenzo-p-dioxin (TCDD); CASRN 1746-01-6. Washington, DC: U.S. Environmental Protection Agency; 2012. <https://iris.epa.gov/static/pdfs/1024_summary.pdf>.

41. Office of Environmental Health Hazard Assessment (OEHHA). Public health goal for TCDD (dioxin) in drinking water. Sacramento, CA: California Environmental Protection Agency, Office of Environmental Health Hazard Assessment; 2010. <https://oehha.ca.gov/media/downloads/water/chemicals/phg/091610tcddphg.pdf>.

42. Office of Environmental Health Hazard Assessment (OEHHA). 2,3,7,8-Tetrachlorodibenzo-p-dioxin and related compounds. Sacramento, CA: California Environmental Protection Agency, Office of Environmental Health Hazard Assessment; 2023. <https://oehha.ca.gov/chemicals/2378-tetrachlorodibenzo-p-dioxin-and-related-compounds>. Accessed 15 Oct 2023.

43. Exon JH, Mather GG, Bussiere JL, Olson DP, Talcott PA. Effects of subchronic exposure of rats to 2-methoxyethanol or 2-butoxyethanol: Thymic atrophy and immunotoxicity. Fundam Appl Toxicol. 1991;16(4):830-840. <https://doi.org/10.1016/0272-0590(91)90168-4>.

44. Smialowicz RJ, Williams WC, Riddle MM, Andrews DL, Luebke RW, Copeland CB. Comparative immunosuppression of various glycol ethers orally administered to Fischer 344 rats. Fundam Appl Toxicol. 1992;18(4):621-627. <https://doi.org/10.1016/0272-0590(92)90123-y>.

45. Singh P, Zhao S, Blaylock BL. Topical exposure to 2-butoxyethanol alters immune responses in female BALB/c mice. Int J Toxicol. 2001;20(6):383-390. <https://doi.org/10.1080/109158101753333668>.

46. Chereshnev VA, Kosareva PV, Samodelkin EI, Sivakova LV. A new experimental model of hemolytic anemia after butoxyethanol and the study of its immunology. Hell J Nucl Med. 2014;17 Suppl 1:7-10.

47. Chemical Manufacturers Association (CMA). 90-day subchronic dermal toxicity study in rabbits with ethylene glycol monobuty ether with cover sheet dated 061289. Washington, DC: WIL Research Laboratories, Inc. for the Chemical Manufacturers Association; 1983. NTIS Document No. OTS0521232. EPA/OTS Document No. 86-890000726. Project No. WIL-81150. <https://ntrl.ntis.gov/NTRL/dashboard/searchResults/titleDetail/OTS0521232.xhtml>.

48. Duprat P, Gradiski D. Percutaneous toxicity of butyl cellosolve (ethylene glycol monobutyl ether). IRCS Med Sci Libr Compend. 1979;7(1):26.

49. Krasavage WJ. Subchronic oral toxicity of ethylene glycol monobutyl ether in male rats. Fundam Appl Toxicol. 1986;6(2):349-355. <https://doi.org/10.1016/0272-0590(86)90250-2>.

50. Grant D, Sulsh S, Jones HB, Gangolli SD, Butler WH. Acute toxicity and recovery in the hemopoietic system of rats after treatment with ethylene glycol monomethyl and monobutyl ethers. Toxicol Appl Pharmacol. 1985;77(2):187-200. <https://doi.org/10.1016/0041-008x(85)90318-7>.

51. Nachreiner DJ. Ethylene glycol butyl ether: Acute vapor inhalation toxicity study in guinea pigs. Washington, DC: Union Carbide Corporation, Bushy Run Research Center for the Chemical Manufacturers Association; 1994. Project ID 94N1392.

52. National Toxicology Program (NTP). NTP technical report on toxicity studies of ethylene glycol ethers 2-methoxyethanol, 2-ethoxyethanol, 2-butoxyethanol (CAS Nos. 109-86-4, 110-80-5, 111-76-2) administered in drinking water to F344/N rats and B6C3F1 mice. Research Triangle Park, NC: U.S. Department of Health and Human Services, Public Health Service, National Institutes of Health, National Toxicology Program; 1993. NTP Toxicity Report No. 26. NIH Publication No. 93-3349. <https://ntp.niehs.nih.gov/sites/default/files/ntp/htdocs/st_rpts/tox026.pdf>.

53. Shepard KP. Ethylene glycol monobutyl ether: Acute dermal toxicity study in the guinea pig. Washington, DC: Eastman Kodak Company, Toxicological Sciences Laboratory for the Chemical Manufacturers Association, Ethylene Glycol Ether Panel; 1994. EGE-58.0-GPIG-EASTMAN. HAEL No. 94-0300. KAN: 902270.

54. National Toxicology Program (NTP). NTP technical report on the toxicology and carcinogenesis studies of 2-butoxyethanol (CAS No. 111-76-2) in F344/N rats and B6C3F1 mice (inhalation studies). Research Triangle Park, NC: U.S. Department of Health and Human Services, Public Health Service, National Institutes of Health, National Toxicology Program; 2000. NTP Technical Report No. 484. NIH Publication No. 00-3974. <https://ntp.niehs.nih.gov/sites/default/files/ntp/htdocs/lt_rpts/tr484.pdf>.

55. Song SH, Kang SK, Choi WJ, Kwak KM, Lee DH, Kang DY, et al. Reticulocytosis in screen-printing workers exposed to 2-butoxyethanol and 2-ethoxyethanol. Ann Occup Environ Med. 2017;29(1):54. <https://doi.org/10.1186/s40557-017-0210-z>.

56. Dodd DE, Snellings WM, Maronpot RR, Ballantyne B. Ethylene glycol monobutyl ether: Acute, 9-day, and 90-day vapor inhalation studies in Fischer 344 rats. Toxicol Appl Pharmacol. 1983;68(3):405-414. <https://doi.org/10.1016/0041-008x(83)90285-5>.

57. Ghanayem BI, Blair PC, Thompson MB, Maronpot RR, Matthews HB. Effect of age on the toxicity and metabolism of ethylene glycol monobutyl ether (2-butoxyethanol) in rats. Toxicol Appl Pharmacol. 1987;91(2):222-234. <https://doi.org/10.1016/0041-008x(87)90103-7>.

58. Starek A, Szymczak W, Zapor L. Hematological effects of four ethylene glycol monoalkyl ethers in short-term repeated exposure in rats. Arch Toxicol. 2008;82(2):125-136. <https://doi.org/10.1007/s00204-007-0236-z>.

59. Eastman Kodak. Subchronic oral toxicity of ethylene glycol monobutyl ether in male rats with cover letter dated 060383. Rochester, NY: Eastman Kodak Company, Toxicology Section; 1983. NTIS Document No. OTS0503697. EPA/OTS Document No. 88-8300509. <https://ntrl.ntis.gov/NTRL/dashboard/searchResults/titleDetail/OTS0503697.xhtml>.

60. Dow Chemical Company (Dow). Inhalation toxicity studies on three samples of ethylene glycol monobutyl ether (Dowanol EB), n-butyl Oxitol - Shell USA, n-butyl Oxitol - Shell Europe. Midland, MI: Dow Chemical Company; 1986. NTIS Document No. OTS0520734. EPA/OTS Document No. 86-890001224. <https://ntrl.ntis.gov/NTRL/dashboard/searchResults/titleDetail/OTS0520734.xhtml>.

61. Wier PJ, Lewis SC, Traul KA. A comparison of developmental toxicity evident at term to postnatal growth and survival using ethylene glycol monoethyl ether, ethylene glycol monobutyl ether, and ethanol. Teratog Carcinog Mutagen. 1987;7(1):55-64. <https://doi.org/10.1002/tcm.1770070108>.

62. Nyska A, Maronpot RR, Ghanayem BI. Ocular thrombosis and retinal degeneration induced in female F344 rats by 2-butoxyethanol. Hum Exp Toxicol. 1999;18(9):577-582. <https://doi.org/10.1191/096032799678845070>.

63. Bauer P, Weber M, Mur JM, Protois JC, Bollaert PE, Condi A, et al. Transient non-cardiogenic pulmonary edema following massive ingestion of ethylene glycol butyl ether. Intensive Care Med. 1992;18(4):250-251. <https://doi.org/10.1007/bf01709843>.

64. Burkhart KK, Donovan JW. Hemodialysis following butoxyethanol ingestion. J Toxicol Clin Toxicol. 1998;36(7):723-725. <https://doi.org/10.3109/15563659809162622>.

65. Dean BS, Krenzelok EP. Clinical evaluation of pediatric ethylene glycol monobutyl ether poisonings. J Toxicol Clin Toxicol. 1992;30(4):557-563. <https://doi.org/10.3109/15563659209017941>.

66. Gijsenbergh FP, Jenco M, Veulemans H, Groeseneken D, Verberckmoes R, Delooz HH. Acute butylglycol intoxication: A case report. Hum Toxicol. 1989;8(3):243-245. <https://doi.org/10.1177/096032718900800307>.

67. Litovitz TL, Bailey KM, Schmitz BF, Holm KC, Klein-Schwartz W. 1990 annual report of the American Association of Poison Control Centers National Data Collection System. Am J Emerg Med. 1991;9(5):461-509. <https://doi.org/10.1016/0735-6757(91)90216-7>.

68. Rambourg-Schepens MO, Buffet M, Bertault R, Jaussaud M, Journe B, Fay R, et al. Severe ethylene glycol butyl ether poisoning. Kinetics and metabolic pattern. Hum Toxicol. 1988;7(2):187-189. <https://doi.org/10.1177/096032718800700215>.

69. Gualtieri JF, DeBoer L, Harris CR, Corley R. Repeated ingestion of 2-butoxyethanol: Case report and literature review. J Toxicol Clin Toxicol. 2003;41(1):57-62. <https://doi.org/10.1081/clt-120018271>.

70. Osterhoudt KC. Fomepizole therapy for pediatric butoxyethanol intoxication. J Toxicol Clin Toxicol. 2002;40(7):929-930. <https://doi.org/10.1081/clt-120016967>.

71. Carpenter CP, Keck GA, Nair JH 3rd, Pozzani UC, Smyth HF Jr, Weil CS. The toxicity of butyl cellosolve solvent. AMA Arch Ind Health. 1956;14(2):114-131.

72. Taylor KW, Howdeshell KL, Bommarito PA, Sibrizzi CA, Blain RB, Magnuson K, et al. Systematic evidence mapping informs a class-based approach to assessing personal care products and pubertal timing. Environ Int. 2023;181:108307. <https://doi.org/10.1016/j.envint.2023.108307>.

1. Present address: Center for Human Health and the Environment, North Carolina State University, Raleigh, North Carolina 27695, USA [↑](#footnote-ref-2)
