# Supplementary material for: Identifying the known and unknown health hazard information for chemical disasters: a phased scoping review of the East Palestine, Ohio train derailment
Source: J Expo Sci Environ Epidemiol. 2025 Sep 12;35(6):888–906. doi: 10.1038/s41370-025-00803-0 (PMC12583197; doi:10.1038/s41370-025-00803-0)
Supplement: Supplementary file 2 — Supplementary information [file 41370_2025_803_MOESM2_ESM.pdf]

Identifying Health Hazard Data Gaps for East Palestine Train Derailment Chemicals

What Happened?

On February 3, 2023,
a Norfolk Southern Railway freight train carrying vinyl chloride and other hazardous chemicals derailed in **East Palestine, Ohio**.

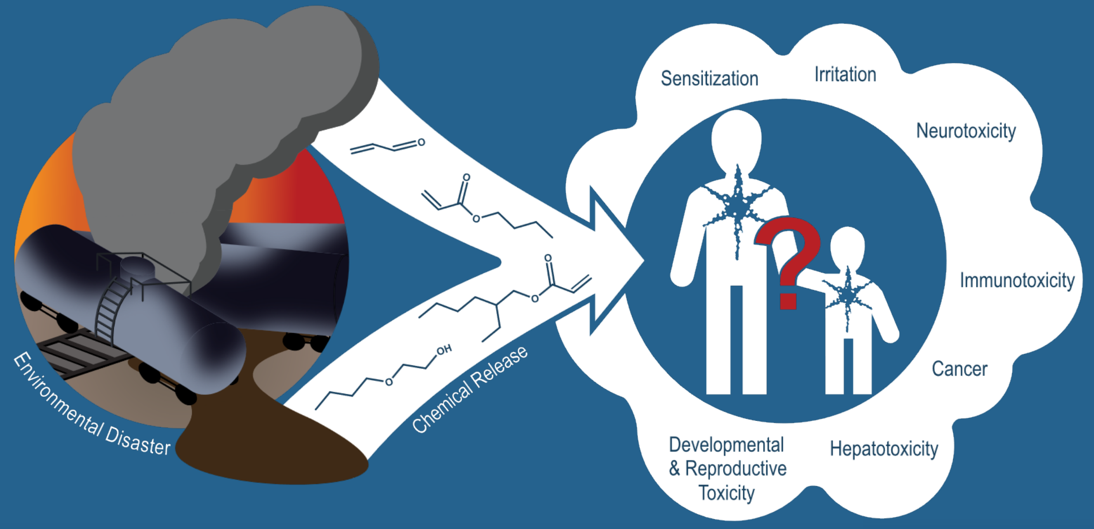

The derailment and a vent and burn that took place over the following days released hazardous chemicals into the environment.

What We Did

We reviewed the environmental health science literature to find information on potential health effects of the chemicals.

Phase 1

Scoping
Identify chemicals and extract authoritative review conclusions for selected health outcomes

Identify Research Gaps
Identify and prioritize chemical-outcome pair research gaps

Phase 2

Literature Reviews
Conduct additional searches for prioritized research gaps, then summarize collective findings

Report and Communicate Findings

What We Found

- All eight higher priority chemicals irritate the skin and eyes, and seven of the eight irritate the respiratory system.
- Two chemicals are **known** to cause cancer, and one chemical **probably** causes cancer. Four others may cause cancer, but more information is needed to draw definitive conclusions.
- Little data was found for noncancer health effects, and many data gaps remain. Two chemicals may cause immune or nervous effects, and two chemicals do or may cause damage to reproductive organs or the developing fetus.
- Little data was found for other organ systems, including the liver, kidney, and heart and blood vessels. Three chemicals were known hazards for other organs.
- Additional literature searches suggest that research is needed for most chemicals, but several studies were found for acrolein and neurological effects.

Reading the Charts

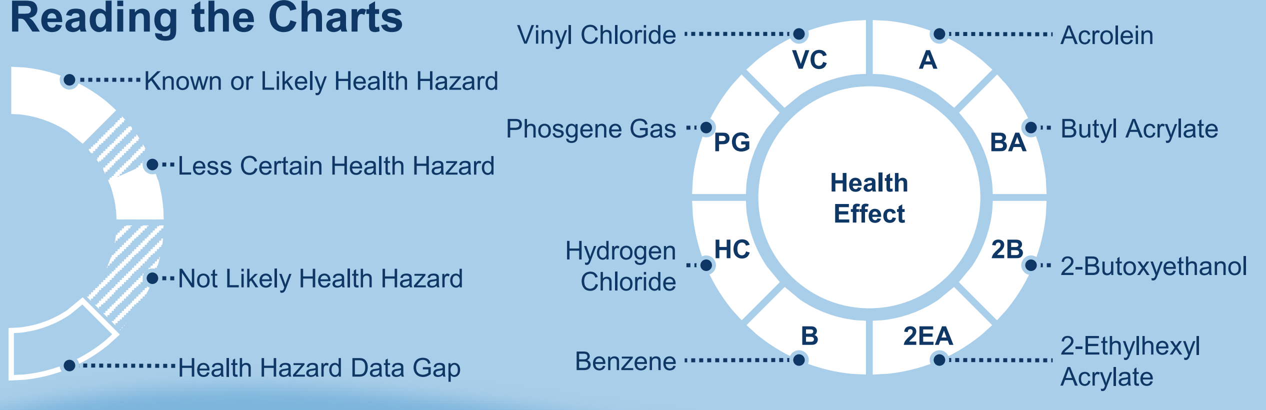

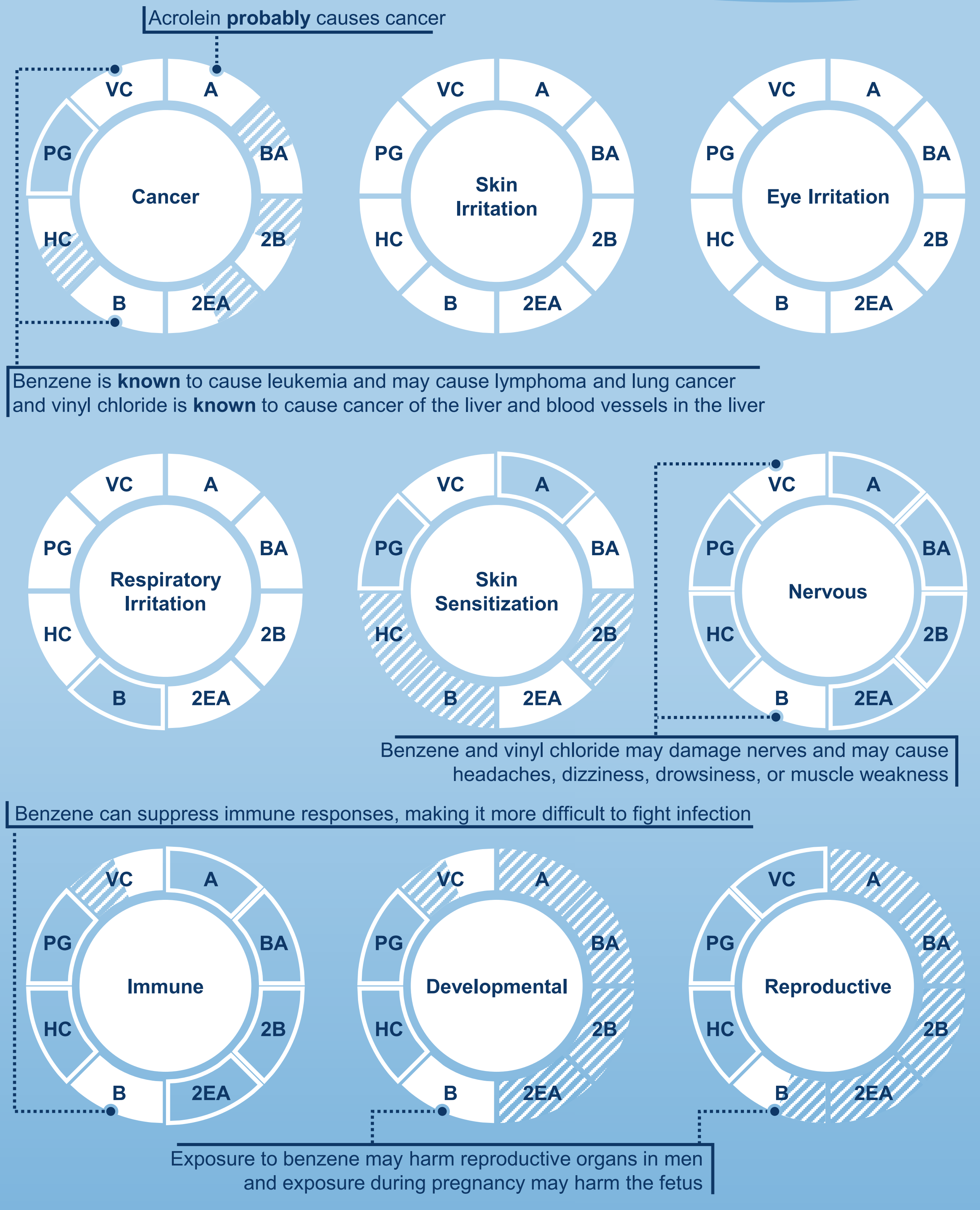

What Does this Mean?

- The community was exposed to many chemicals after the derailments, but we report health hazards from exposure to a single chemical.
- An individual's risk of health effects depends on the nature of the exposure and individual characteristics. For example, some people may have higher and longer exposures; exposure to many chemicals; genetic predispositions; and existing diseases or stress, which can all increase risk.
